# Tuning the Activity–Stability Balance of Photocatalytic Organic Materials for Oxidative Coupling Reactions

**DOI:** 10.1021/acsami.2c01646

**Published:** 2022-03-29

**Authors:** Alicia Jiménez-Almarza, Alberto López-Magano, Rubén Mas-Ballesté, José Alemán

**Affiliations:** † Department of Inorganic Chemistry (Module 7), Facultad de Ciencias, Universidad Autónoma de Madrid, 28049 Madrid, Spain; ‡ Institute for Advanced Research in Chemical Sciences (IAdChem), Universidad Autónoma de Madrid, 28049 Madrid, Spain; § Department of Organic Chemistry (Module 1), Facultad de Ciencias, Universidad Autónoma de Madrid, 28049 Madrid, Spain

**Keywords:** organic materials, photocatalysis, oxidative coupling, amines, imines

## Abstract

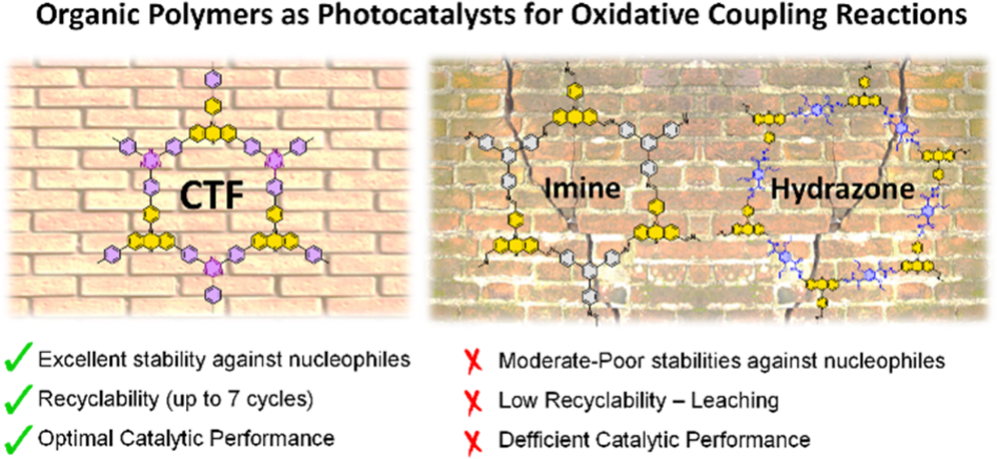

Three materials containing a photoactive unit, 10-phenyl phenothiazine (PTH), have been studied for the visible light-mediated oxidative coupling of amines. In particular, the materials considered are assembled through the condensation of extended polyimine, polyhydrazone, or polytriazine frameworks. These three materials present different stabilities in the presence of strong nucleophiles such as amines, which is a key factor for efficient catalytic performance. In the series of materials reported herein, the triazine-based material shows the optimal compromise between activity and stability when studied for the oxidative coupling of amines, achieving imine products. Accordingly, while significant leaching of molecular active fragments is ruled out for triazine-based polymers, other materials of the series show a significant chemical erosion as a result of the reaction with the amine substrates. Consequently, only a triazine-based material allows performing several catalytic cycles (up to seven) with yields higher than 80%. The applicability of this heterogeneous catalyst has been proven with a variety of substrates, confirming its stability and obtaining diverse imine coupling products with excellent yields.

## 1 Introduction

Photocatalysis is a particularly attractive methodology that can be applied in organic synthesis for the production of molecules of different complexities with industrial relevance in an efficient and environmentally benign way.^1−4^ To reach this goal, the use of photocatalysts capable of harvesting light energy is necessary to trigger, accelerate, or direct reactions that would have kinetic and/or thermodynamic unreachable barriers in the absence of light.^5^ The success of a specific process lies in the design of the appropriate photocatalyst, which should be efficient, active, and chemically robust. In addition to good catalytic activity, the easy separation and recyclability of catalytic materials appear as a major asset.^6,7^ Therefore, owing to their inherent advantages, the research for improved catalytic systems has fueled the investigation of new photoactive materials, which is nowadays a blossoming research field.^8^

A special family of heterogeneous photocatalysts is organic materials, such as porous organic polymers (POPs), covalent triazine frameworks (CTFs), covalent organic frameworks (COFs), and related covalent architectures.^9−11^ A likable feature that makes these materials interesting is the wide range of possibilities in the predetermined design of building blocks that leads to controlling the structure and chemical properties of the synthesized frameworks in a very precise manner.^6,12−14^ The choice of building blocks is crucial, not only to confer a good photocatalytic activity to the material but also to achieve a high degree of stability that allows material recyclability. Thus, misguided design can result in materials with excellent optical properties for a photocatalytic reaction with poor stability or can produce very robust materials but with poor photocatalytic characteristics. Therefore, it is necessary to consider an optimal balance in choosing an active building block and determining a robust assembling strategy to perform an efficient photocatalytic reaction.

**Figure 1:**
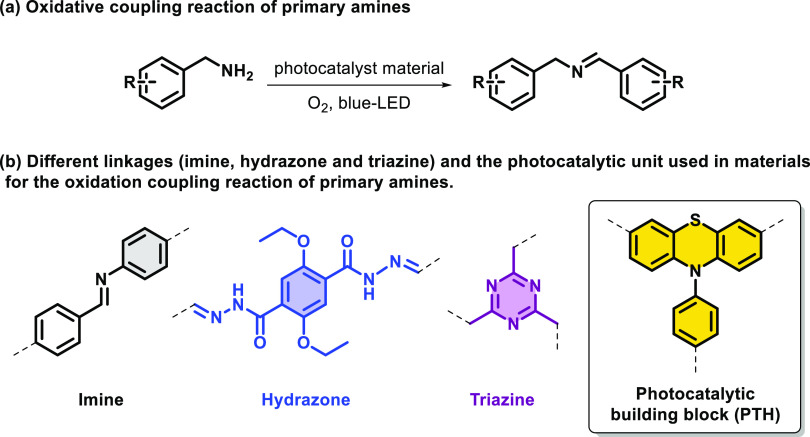
Reaction studied in this work (a) and the chosen photocatalytic unit incorporated into the studied materials through three different linkages (b).

Oxidative coupling reactions are an engaging photocatalytic type of transformation in which predesigned organic materials can be applied,^15−18^ allowing the use of O_2_ as an oxidant, which fulfills green chemistry requirements. For instance, the reaction between benzylic amines through oxidative coupling is an efficient and versatile method to obtain a variety of imines with different structures (Figure 1a).^19−21^ Coupling of two nucleophilic precursors is achieved in these processes because light-mediated oxidation triggers the conversion of one of the nucleophiles into the corresponding electrophile, giving rise to the coupled product.^22,23^ However, the nucleophilicity of amine reagents might be an important drawback for the use of some kind of extended organic materials. Therefore, undesired disassembly processes can be observed as a result of the attack of the substrate (a nucleophilic amine) on the material’s iminic backbone.^24^ Thus, although imines are one of the most used linkages to build polycondensated materials, they can hardly be employed in the oxidative coupling of amines.

The scarcity in the number of photocatalytic extended organic materials used in oxidative couplings in the literature prompted us to investigate the design principles that should be considered to achieve such transformation in an efficient way. Although the main goal of this work is to perform a comparative study on the balance between the stability and photocatalytic activity of materials with different linkages, we have included a benchmarking table involving (photo)catalytic oxidative coupling reactions using different porous materials (see the Supporting Information, Table S8). Therefore, we chose three organic materials assembled through different linkages (imine,^25^ hydrazone,^26^ and triazine,^10^Figure 1b), with the aim to study their activity/stability balance under photocatalytic conditions in the presence of strong nucleophilic amines. As the photocatalytic unit, we chose 10-phenyl phenothiazine (PTH), whose molecular version is a widely used organic photocatalyst applied in photoactivated dehalogenation or oxidation reactions (bottom-right, Figure 1).^27−29^ The results observed for oxidative couplings of amines, using these series of materials (imine, hydrazone, and triazine linkers), highlight the need for considering the factors governing the stability in the design of photocatalytic extended organic materials.

## 2 Experimental Section

10-Phenyl-10*H*-phenothiazine (**2**),^30^ 3,7-dibromo-10-(4-bromophenyl)-10*H*-phenothiazine (**3**),^31^ 10-(4-formylphenyl)-10*H*-phenothiazine-3,7-dicarbaldehyde (**4**),^31^ diethyl 2,5-diethoxyterephthalate (**6a**),^32^ 2,5-diethoxyterephthalohydrazide (**6b**),^32^ and imine-based material **7**(31) were synthesized according to previously reported procedures. For their characterization, see the Supporting Information. Their spectroscopic information match that described in the literature.

### 2.1 4,4′-(10-(4′-Cyano-[1,1′-biphenyl]-4-yl)-10*H*-phenothiazine-3,7-diyl)dibenzonitrile (**5**)

A solution of **3** (0.92 g, 1.8 mmol) and (4-cyanophenyl)boronic acid (0.80 g, 5.4 mmol) in tetrahydrofuran (THF) (42 mL) was added to a reflux tube with a magnetic stir bar, followed by the addition of 8.4 mL of an aqueous solution of K_2_CO_3_ (1 g, 7.2 mmol). The reaction mixture was stirred under an inert atmosphere for 10 min. Then, Pd(PPh_3_)_4_ (105 mg, 0.1 mmol) was added. The reaction was refluxed for 48 h under an inert atmosphere at 70 °C. After that, the mixture was cooled down to room temperature, THF was evaporated, and the crude was extracted with five portions of 50 mL of dichloromethane (DCM). The organic phases were separated, dried over Na_2_SO_4_, filtrated, and evaporated. The mixture was purified by flash column chromatography (*c*-Hex/DCM = 1:99 v/v) to afford **5** in 94% yield as a shiny yellow solid.^1^H NMR (300 MHz, CDCl_3_) δ 7.89 (d, *J* = 8.0 Hz, 2H), 7.80 (s, 4H), 7.68 (d, *J* = 8.1 Hz, 4H), 7.56 (dd, *J* = 7.8, 5.8 Hz, 6H), 7.27 (dd, *J* = 1.1 Hz, 2H), 7.11 (dd, *J* = 8.5, 1.3 Hz, 2H), 6.32 (d, *J* = 8.7 Hz, 2H) ppm.^13^C NMR (75 MHz, CDCl_3_) δ 143.9, 143.8, 140.8, 139.7, 138.7, 133.9, 132.9, 132.7, 131.4, 130.0, 127.8, 126.8, 125.9, 125.3, 120.7, 118.9, 116.5, 111.8, 110.7 ppm. Elemental analysis calculated for (C_39_H_22_N_4_S): C: 80.95%; H: 3.83%; N: 9.68%; S: 5.54%. Found: C: 79.55%; H: 4.17%; N: 9.18%; S: 5.24%.

### 2.2 Synthesis of Hydrazone-Based Material **8**

A 25 mL solvothermal reactor was charged with building block **4** (42.0 mg, 0.12 mmol) and building block **6b** (49.0 mg, 0.18 mmol). Then, a mixture of 0.6 mL of anhydrous dioxane, 5.4 mL of mesitylene, and 0.6 mL of 6 M aqueous acetic acid was added. The reactor was sealed and heated at 120 °C for 72 h. The resulting orange powder was isolated by filtration and washed with DMF, MeOH, and THF. The resulting solid was immersed in anhydrous THF for 24 h. Then, it was dried under vacuum for 12 h at room temperature and at 100 °C for 2 h, affording an orange powder, hydrazone-based material **8** (72% yield). Elemental analysis calculated for (C_78_H_68_N_14_O_12_S_2_)_1_(H_2_O)_7_: C: 59.15%; H: 5.22%; N: 12.38%; S: 4.05%. Found: C: 59.61%; H: 5.10%; N: 11.75%; S: 3.91%.

### 2.3 Synthesis of CTF **9**

A solution of the building block **5** (50 mg, 0.09 mmol) in 5 mL of DCM was added dropwise into an oven-dried sealed tube equipped with a magnetic stir bar that contained 0.25 mL of triflic acid. The reaction was refluxed for 24 h at 100 °C. Then, the mixture was cooled down to room temperature and quenched with EtOH (20 mL). After 30 min, the material was isolated by filtration and washed with DCM, EtOH, MeCN, and Et_3_N. The resulting powder was dried at room temperature under vacuum for 24 h to afford an orange powder, CTF **9** (94% yield). Elemental analysis calculated for (C_39_H_22_N_4_S)_10_(C_6_H_15_N)_1_(H_2_O)_40_: C: 71.97%; H: 4.80%; N: 8.69%; S: 4.85%. Found: C: 71.98%; H: 4.83%; N: 8.52%; S: 5.13%.

### 2.4 Oxidative Coupling of Primary Amines under the Catalysis of Materials **7**, **8**, or **9**

Two milligrams of the corresponding material (**7**, **8**, or **9**) and 2 mL of acetonitrile as the solvent were added into an oven-dried 10 mL vial equipped with a magnetic stir bar. Then, the corresponding amine **10** (0.2 mmol) was added. The vial was closed with a poly(tetrafluoroethylene) (PTFE)/rubber septum, and oxygen was bubbled in the reaction mixture for 5 min. The reaction mixture was stirred under blue light-emitting diode (LED) irradiation for 14 h with an O_2_ balloon at 25 °C. The yield was determined by ^1^H NMR using the quantitative standard. Then, the crude was filtered through a membrane filter and evaporated under reduced pressure, affording pure imines **11**.

## 3 Results and Discussion

### 3.1 Synthesis and Characterization of the Materials

**Scheme 1:**
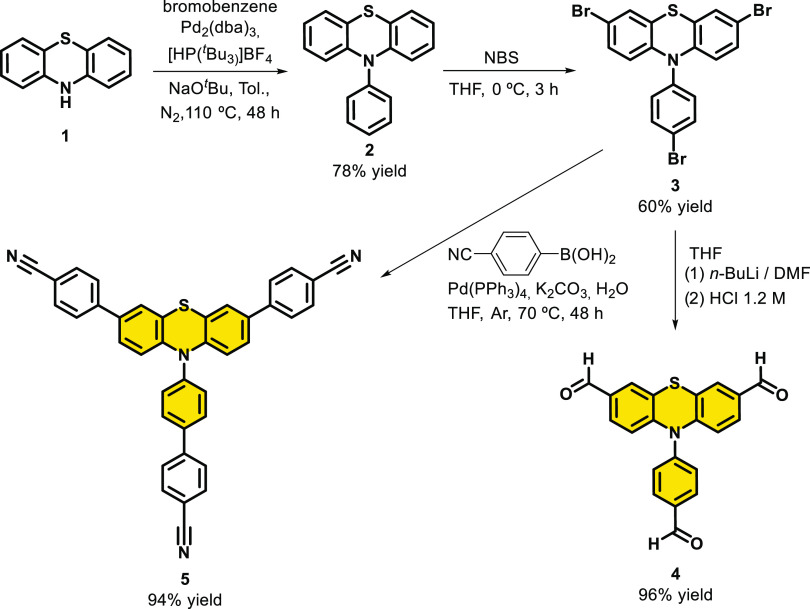
Synthesis of Building Blocks **4** and **5**

**Scheme 2:**
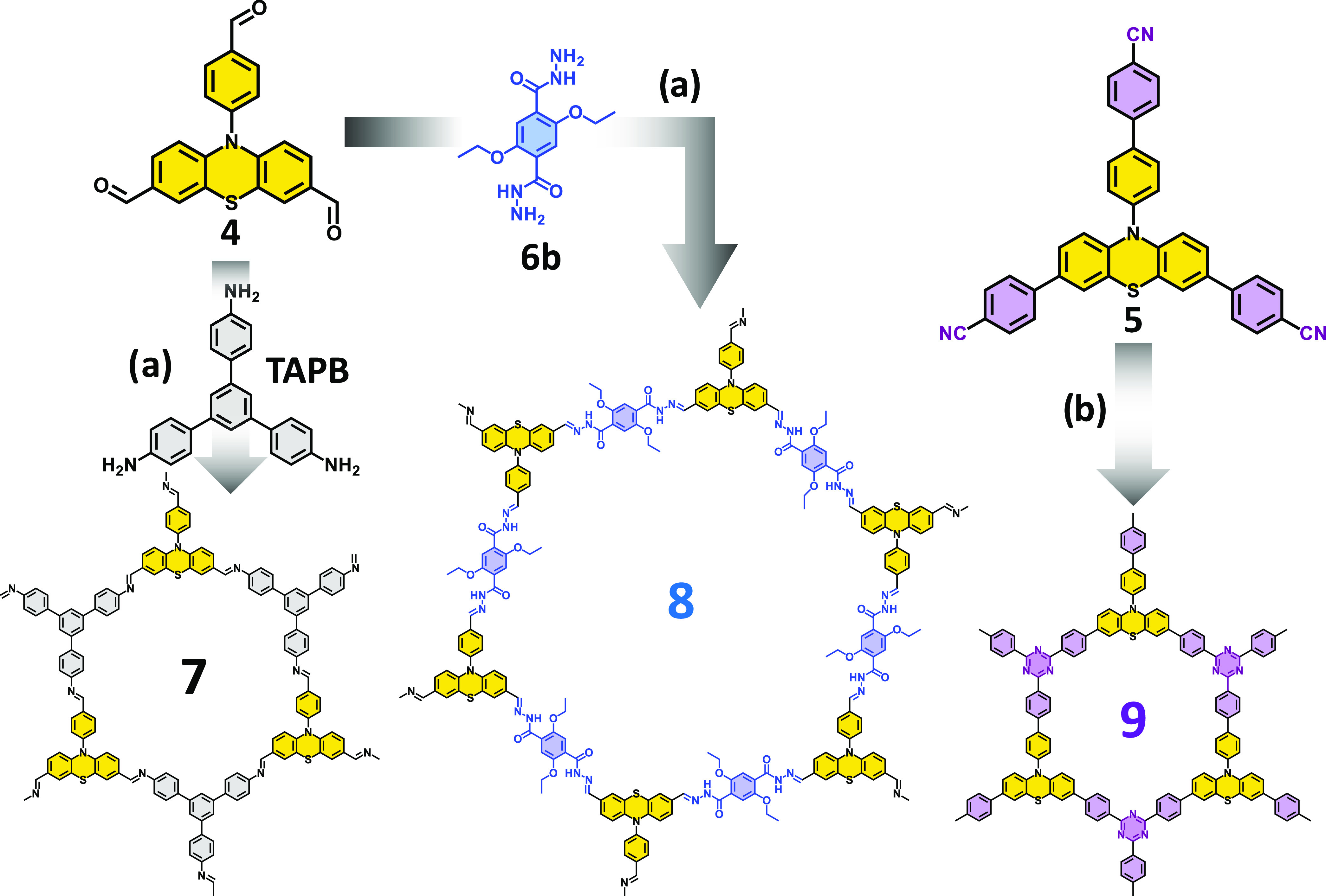
Synthesis of Materials **7**, **8**, and **9** Conditions for (a) 6 mL of dioxane/mesitylene (1:9) and 0.6 mL of acetic acid (6 M) at 120 °C during 72 h; conditions for (b) 0.25 mL of trifluoromethanesulfonic acid and 5 mL of DCM at 100 °C during 24 h.

The general synthetic strategy followed in this work is presented in Scheme 1 (building blocks) and Scheme 2 (material synthesis). From the commercially available heterocycle **1**, a Buchwald–Hartwig reaction (Csp^2^–N bond) was carried out to form the *N*-arylated product **2**. Then, bromination was performed using NBS to afford the intermediate **3** in 60% yield. Building blocks **4** and **5** were synthesized from the brominated intermediate **3**. Thus, aldehyde **4** was obtained when a solution of intermediate **3** was treated with *n*-butyl-lithium (*n*-BuLi, 1.6 M in THF), followed by dry DMF at −78 °C, yielding the orange building block (**4**) in 96% yield after flash chromatography purification. The synthesis of **5** was carried out through a Suzuki reaction between (4-cyanophenyl)boronic acid and **3**. The reaction took place during 48 h at reflux in THF under an inert atmosphere in the presence of K_2_CO_3_ and [Pd(PPh_3_)_4_]. Afterward, the yellow building block (**5**) was obtained in 94% yield after purification by flash chromatography. Products **2**–**5** were characterized by ^1^H and ^13^C NMR and elemental analysis (see the Supporting Information).

Once we synthesized the building blocks, materials **7**–**9** were obtained using two different conditions (see Scheme 2). Material **7** resulted from the condensation of reagent **4** (which contains the PTH fragment) and the **TAPB** building block, which is commercially available. We also synthesized the hydrazone-based material **8**, which was formed by the condensation of reagent **4** and compound 2,5-diethoxyterephthalohydrazide^32^ (**6b**). For both materials **7** and **8**, reaction conditions were applied on a dioxane/mesitylene 1:9 mixture in the presence of aqueous acetic acid during 72 h at 120 °C. In addition, material **9** was generated by the cyclotrimerization of 4,4′-(10-(4′-cyano-[1,1′-biphenyl]-4-yl)-10*H*-phenothiazine-3,7-diyl)dibenzonitrile (**5**) using triflic acid at 100 °C for 24 h. Then, the reaction was quenched with EtOH and filtrated. The resulting solid was washed with Et_3_N to neutralize any resting amounts of triflic acid, which usually results in the protonation of the PTH fragment, altering its optical properties.

**Figure 2:**
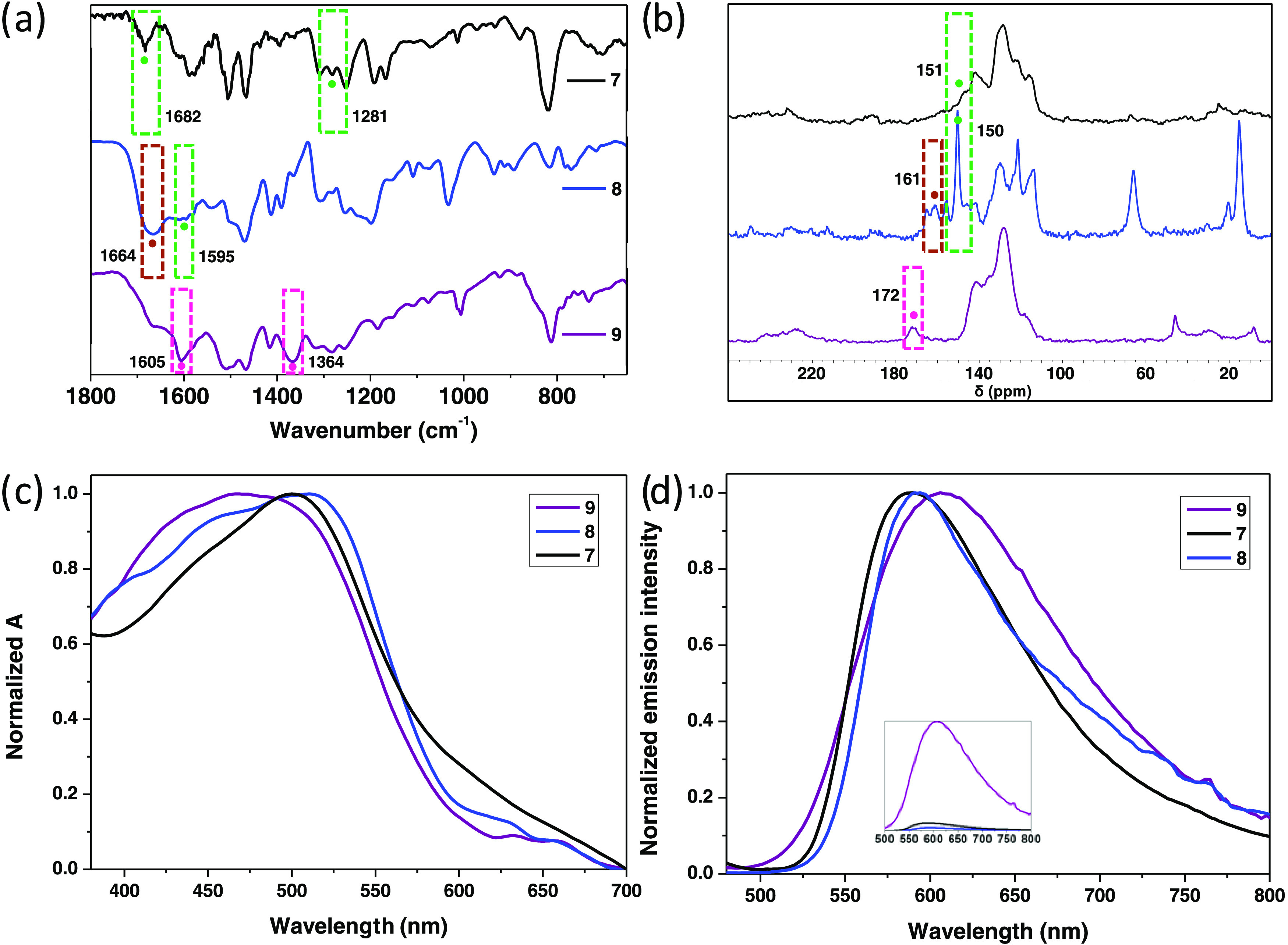
(a) FT-IR spectra, (b) CP-MAS-^13^C NMR spectra, (c) diffuse reflectance spectroscopy (DRS) spectra, and (d) emission spectra (right, λ_exc_ = 450 nm) of materials **7** (black data), **8** (blue data), and **9** (purple data). Signals due to C=N imine moieties (green mark), C=O groups (brown mark), and C=N of triazine fragments (pink mark).

The formation of the expected structures was initially confirmed by Fourier-transform infrared spectroscopy (FT-IR, see Figure 2a). The formation of the polyiminic material **7** was confirmed by the appearance of two typical vibration signals at 1682 and 1281 cm^–1^, assigned to the C=N and C–C=N–C stretching of imine fragments. Material **8** shows signals at 1664 cm^–1^ (C=O stretching) and 1595 cm^–1^ (C=N stretching) characteristic of hydrazine groups.^32^ Furthermore, for **9**, the presence of triazine fragments is revealed by the observation of signals at 1605 and 1364 cm^–1^.^33^ The relative content of S, N, C, and H determined by elemental analysis matched with the general formula C_45_H_28_N_4_S·6H_2_O (for **7**), C_78_H_68_N_14_S_2_·7H_2_O (for **8**), and C_39_H_22_N_4_S·4H_2_O·0.1C_6_H_15_N (for **9**) (see Section 2). These data are in agreement with the formation of the expected frameworks and also indicate the tendency of such materials to interact with small solvent molecules through noncovalent interactions. Moreover, solid-state ^13^C NMR experiments using the cross-polarization technique combined with magic angle spinning (CP-MAS NMR) exhibited the characteristic peaks expected for imine, hydrazone, and triazine groups in materials **7**, **8**, and **9**, respectively (see Figure 2b). In particular, while for material **7**, the iminic carbon is observed at 151 ppm,^34^ for material **8**, the C=N carbon appears at 150 ppm and the C=O carbon at 161 ppm.^32^ Triazine carbon atoms in CTF **9** appear at 172 ppm.^33^ The thermogravimetric analysis of the three polymers showed good thermal stabilities up to 350 °C in all of the cases (Figures S25–S27 in the Supporting Information)

Optical characteristics of the three materials were examined by absorption diffuse reflectance spectroscopy (DRS) and emission (excitation at 450 nm) measurements (see Figure 2c,d). It is worth noting that while **7** and **8** show similar features in both absorption and emission spectra, **9** absorbs at higher energies (maximum at 501 nm for **7**, 512 nm for **8**, and 469 nm for **9**) and emits at lower energies (maximum at 588 nm for **7**, 592 nm for **8**, and 607 nm for **9**). Absorption data show comparable intensities for the three materials studied; therefore, for clarity purposes, normalized spectra are shown in Figure 2c. In contrast, CTF **9** shows the emission of much higher intensity than those observed for **7** and **8**.

**Figure 3:**
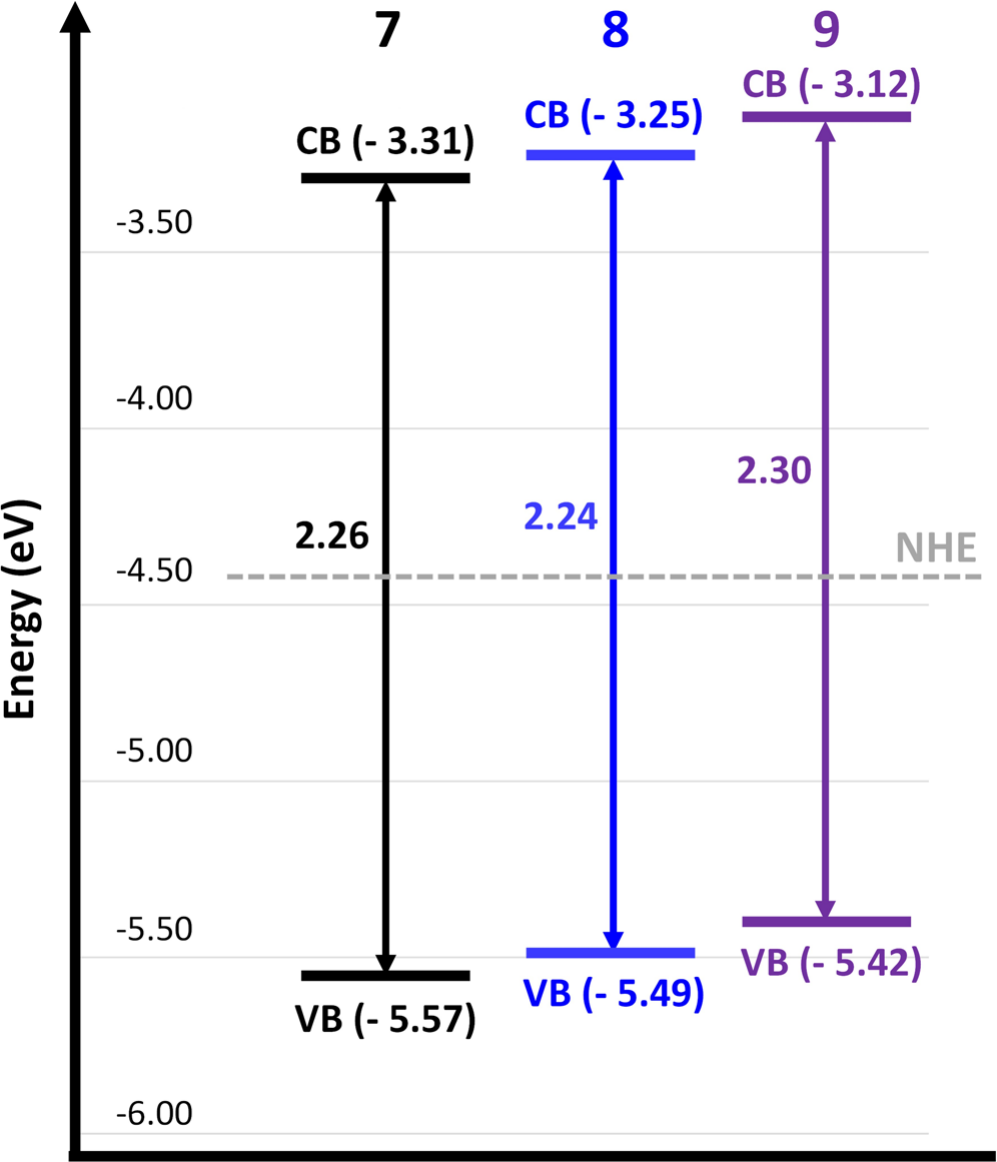
Energy levels of materials **7**, **8**, and **9**. Valence band (VB) values were obtained from the first oxidation signal that appeared on the cyclic voltammetry curve. Band gap values were calculated from the application of Kubelka–Munk theory to DRS experiments.

From diffuse reflectance data, band gaps were calculated through Kubelka–Munk plots (2.26 eV for material **7**, 2.24 eV for **8**, 2.30 eV for **9**; see Figures S13–S15 of Supporting Information). These data together with electrochemical measurements allowed us to determine the energy levels. Interestingly, only slight differences between materials **7**, **8**, and **9** were observed by cyclic voltammetry (Figures S28–S30 in the Supporting Information). Therefore, oxidation signals from electrochemical measurements indicate that the valence bands of **7**, **8**, and **9** were located at −5.57, −5.49, and −5.42 eV, respectively (see Figure 3). Overall, the combination of absorption and electrochemistry experiments shows very similar energy values of valence and conduction bands of the three materials, which can be indicative that such photophysical features are due to the same PTH fragment. Accordingly, similar photocatalytic activities are expected for **7**, **8**, and **9**. Indeed, as shown below, the photocatalytic oxidative coupling of amines is similarly achieved in the three cases.

**Figure 4:**
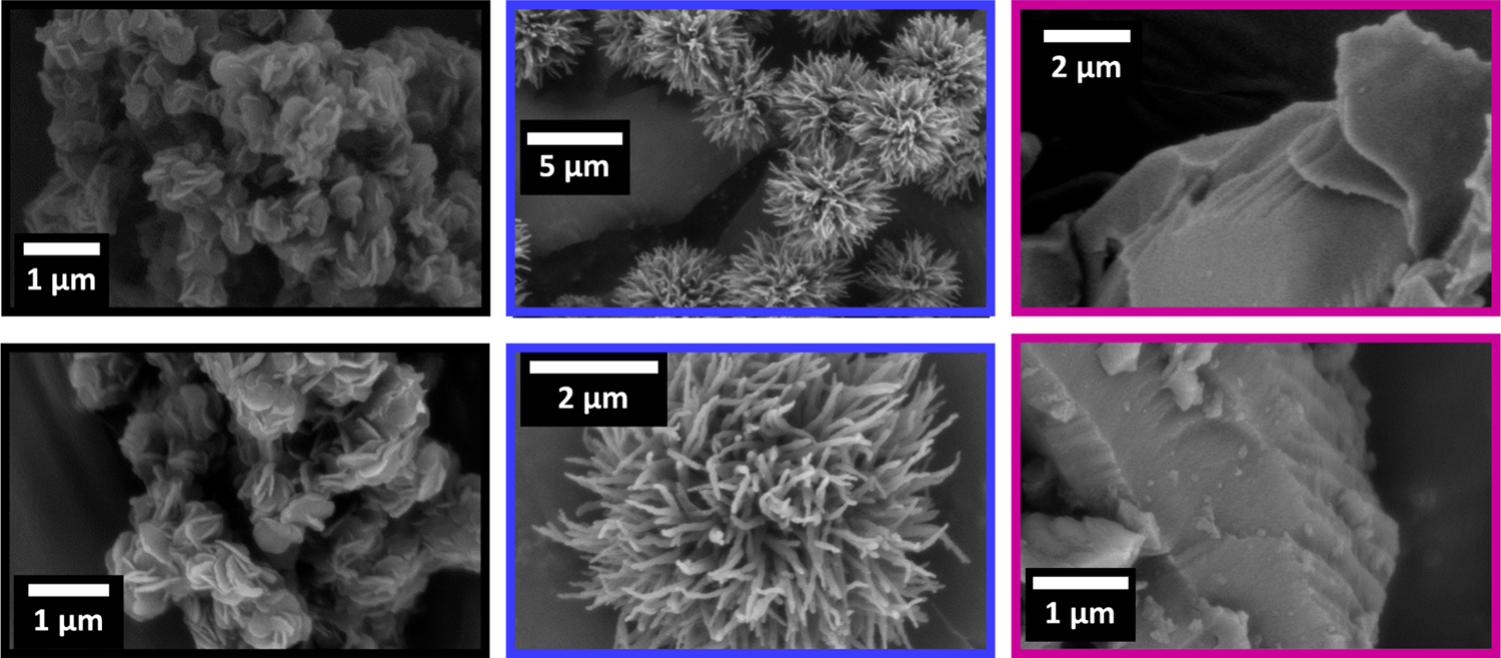
SEM images of materials **7** (black), **8** (blue), and CTF **9** (purple).

The microstructure of these materials was examined by scanning electron microscopy (SEM) (Figure 4). For material **7**, as reported before,^34^ the layered hexagonal symmetry was observed as hexagonal platelets. In contrast, material **8** showed fibers, suggesting that the layered structures collapsed into tubular architectures. In addition, **9** showed a two-dimensional (2D) layered microstructure with lateral dimensions larger than those observed for **7**. Furthermore, powder X-ray diffraction (PXRD) and N_2_ adsorption measurements indicated that materials **7**, **8**, and **9** are amorphous and present low porosity (see the Supporting Information, Figures S19–S21). The amorphous and low porous nature of material **7** is in accordance with previously reported data.^34^ Moreover, the low porosity and amorphous nature of material **8** can be easily related to the observed microstructure, which significantly deviates from a layered material. Observations made for CTF **9** are in good agreement with previously observed data for several covalent triazine frameworks.^10^

### 3.2 Photocatalytic Activity: Oxidative Coupling of Primary Amines

**Figure 5:**
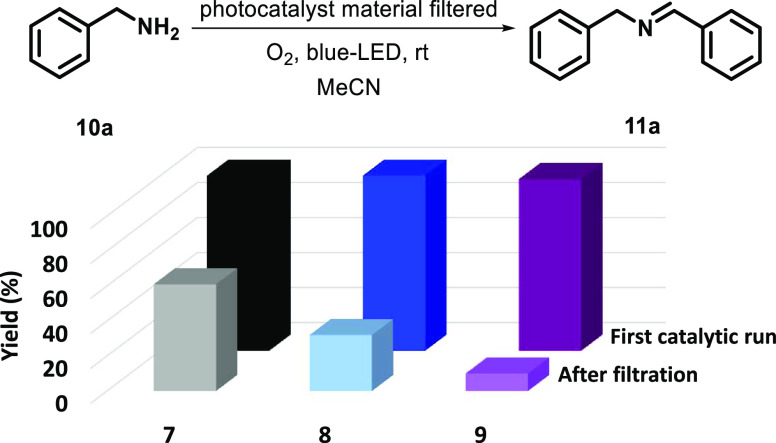
First catalytic run: imine product yield after 14 h under blue LED irradiation before filtering off the photocatalytic materials **7** (black data), **8** (blue data), or **9** (purple data). After filtration: imine product yield after 14 h under blue LED irradiation after filtration of the photocatalytic materials **7**, **8**, or **9** for the second run. All reactions were performed using **10a** (0.2 mmol) and 2 mg of the photocatalytic material (only in the first run because it was filtered before the second run) in 2 mL of acetonitrile. Yield (%) was determined with an internal standard (see the Supporting Information).

The photocatalytic application of the series of three organic materials obtained (**7**–**9**) was examined by studying the oxidative coupling of primary amines. Initially, the formation of the imine product **11a** from the coupling of benzylamine **10a** was analyzed according to the reaction shown in Figure 5, performed under blue light irradiation at 25 °C in acetonitrile (see above for details). The corresponding blank experiments gave null conversions (see the Supporting Information, Table S1). It is worth mentioning that the catalytic activity of the three materials was very high (close to 100% conversion). Then, the possibility of leaching of molecular active species into the reaction medium during the photocatalytic process should be checked. With the aim of analyzing this issue, after the first catalytic run, the reaction mixture was filtrated to eliminate the powdered material. Afterwards, to the solutions resulting from filtration, an additional 0.2 mmol of benzylamine (**10a**) was added. The mixture was allowed to react for another 14 h under blue light irradiation, and the formation of the product was measured by ^1^H NMR. According to the data shown in Figure 5, while material **7** showed the formation of significant amounts of the imine product after removing the catalytic material (61% imine yield), this effect was negligible for **9** (<10% of the imine product **11a**). Material **8** showed an intermediate value for the amount of product observed after the filtration process (32%). This initial observation clearly indicates the distinct stabilities of the materials under the reaction conditions. Although yields could seem satisfactory, the leaching test demonstrated that a significant amount of the product was formed through homogeneous catalysis when materials **7** and **8** were used. Overall, these results suggested that CTF **9** was the most stable material of the series, hydrazone-based material **8** showed intermediate stability, and imine-based material **7** demonstrated a poor sturdiness against nucleophiles.

**Figure 6:**
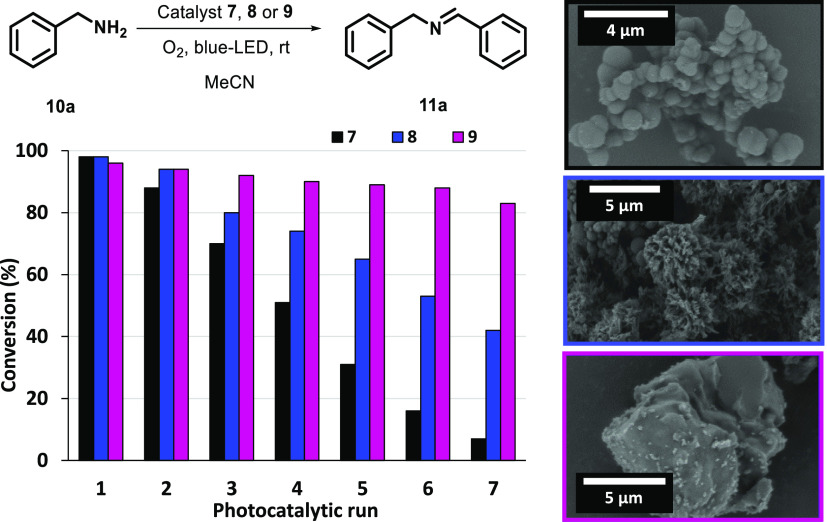
Left: photocatalytic runs with material **7** (black data), material **8** (blue data), and CTF **9** (purple data) in the oxidative coupling of amine **10a**. All cycles were carried out using **10a** (0.2 mmol), and the initial amount of photocatalyst material recovered by centrifugation (3 mg in the first run) in 2 mL of acetonitrile under blue LED irradiation. Right: SEM images of the materials after three photocatalytic runs.

The same stability trend was confirmed by performing a series of successive catalytic experiments in which, after each run, the material was recovered by centrifugation and reused in a new catalytic cycle. Following this procedure, seven photocatalytic runs were carried out under blue LED irradiation at 25 °C for the three materials of the series studied in this work. As shown in Figure 6, the degradation of imine-based material **7** considerably reduced product formation in each run, preventing its recyclability with only 7% yield obtained after the 7th catalytic run. In the case of **9**, the product conversion was above 80% until the 7th cycle, while the hydrazone-based material **8** showed an intermediate behavior. According to the different stabilities for materials **7**, **8**, and **9** found in the experiments shown above, SEM images clearly show the different structural erosions suffered by these photocatalysts after being recycled three times (Figure 6, right). Interestingly, while the layered appearance of **9** remained intact in the SEM images after three catalytic runs, the fibrous microstructure of **8** was partially altered and the platelet features of **7** were completely degraded (compare Figures 6 and 4).

**Scheme 3:**
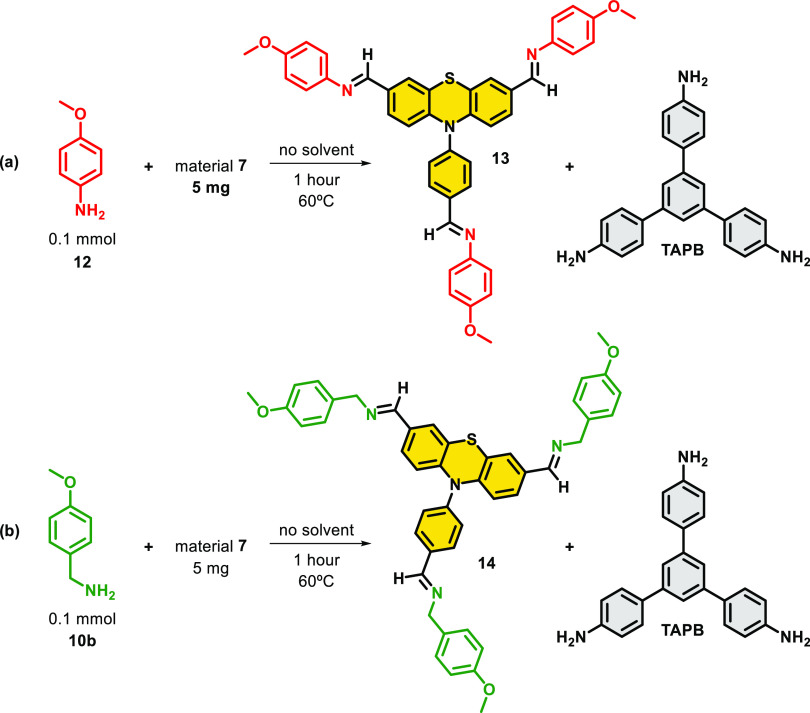
Chemical Evolution of Material **7** as a Result of the Reaction with (a) Methoxyaniline (**12**) or (b) (4-Methoxyphenyl)methanamine (**10b**) for 1 h at 60 °C

To elucidate the chemical process that triggers the erosion of material **7**, we examined the evolution of this material in the presence of nucleophilic amines. As model nucleophilic amines, methoxyaniline (**12**) or (4-methoxyphenyl)methanamine (**10b**) were employed. In these experiments, material **7** (5 mg) and 0.1 mmol of **12** or **10b** were stirred for 1 h at 60 °C in the absence of a solvent (Scheme 3, see the Supporting Information, Section 8 for details). Then, CDCl_3_ was added, and the solution was analyzed by ^1^H NMR. The obtained data allowed us to identify and quantify the degradation products shown in Scheme 3 as a result of the nucleophilic attack of the amine to the imine groups of the material. Analysis of the ^1^H NMR spectra revealed that while 20% of **7** reacted with **12** (Scheme 3a), when the same material was exposed to **10b**, 11% of **7** was deteriorated (Scheme 3b). The same procedure was used to evaluate the degradation pathways of **8** and **9**. However, owing to their increased stabilities (**8** and **9**), under these specific conditions, we were not able to quantify the possible chemical erosion by NMR. To do this, we performed an additional set of experiments using inductively coupled plasma atomic emission spectroscopy (ICP-OES) on samples generated after 24 h of digestion with nucleophilic amine **10b**. The analytical signal for quantification of material degradation was the S content. Interestingly, according to this analysis (see Table S6 of Supporting Information), 10% of material **8** was leached into the reaction medium under these new digestion conditions. In contrast, the irreversible and robust nature of triazine linkage on CTF **9** avoids disassembly processes, being the chemical erosion lower than 1%. In good agreement with the enhanced degradation of material 7, these digestion experiments revealed 30% leaching. Accordingly, these observations agree with the observed recyclability and stability of these materials (see Figures 5 and 6).

**Scheme 4:**
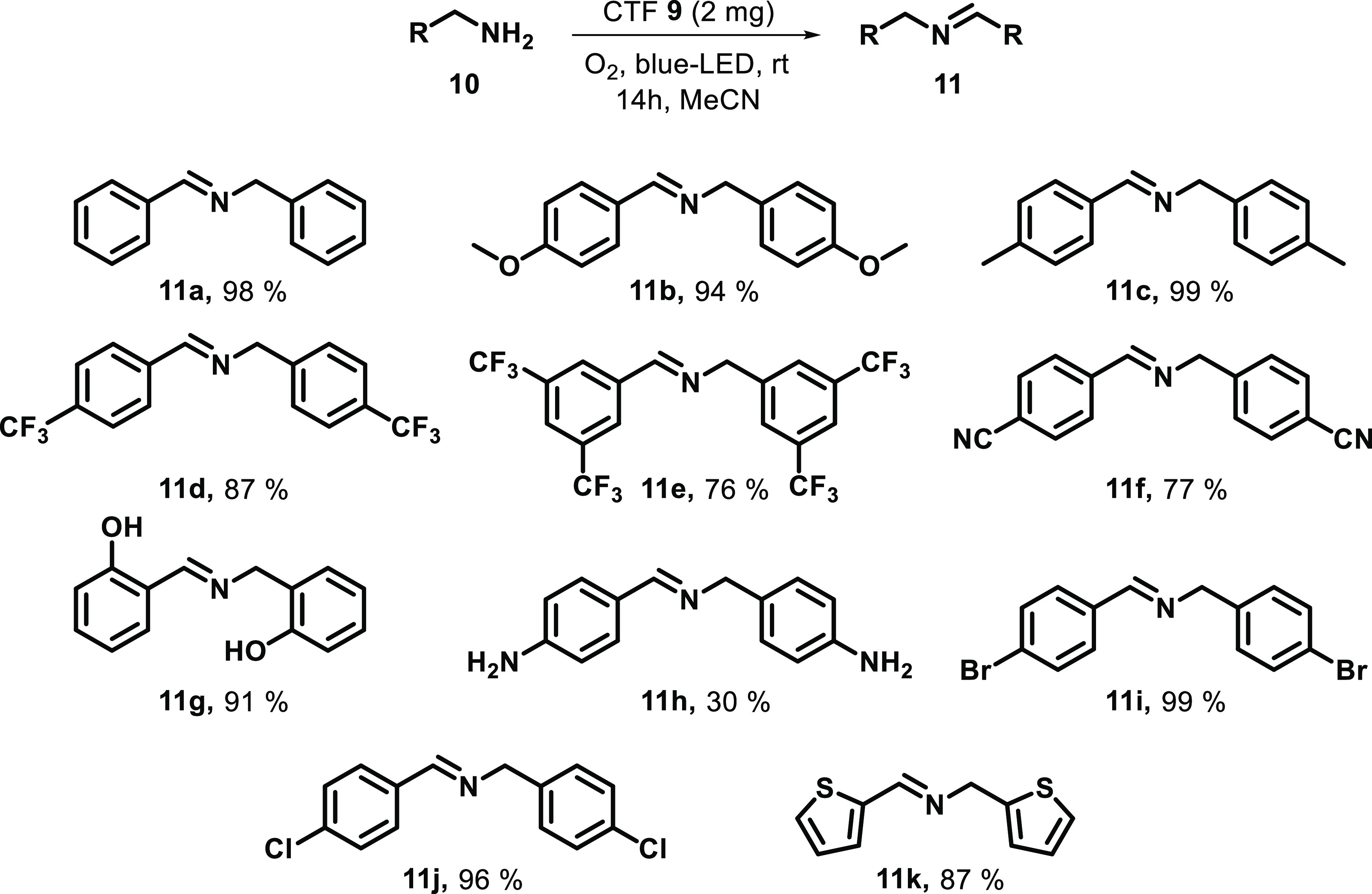
Scope of Oxidative Coupling of Amines All reactions were carried out using **10** (0.2 mmol) and 2 mg of CTF **9** as a photocatalyst in 2 mL of acetonitrile under blue LED irradiation. The yields were determined by ^1^H NMR with 1,3,5-trimethoxybenzene (0.1 mmol) as the quantitative standard.

Once it was determined that the optimal material to perform the photocatalytic oxidative coupling of amines was **9**, the scope of this transformation was evaluated. Therefore, the reaction was optimized (see Table S2 of Supporting Information) to perform the oxidative coupling of eleven primary amines with different electron-donating and electron-withdrawing groups (Scheme 4). The reaction allowed the use of different electron-donating groups, such as MeO (**10b**) and Me (**10c**), as well as electron-withdrawing groups, such as CF_3_ (**10d** and **10e**) or CN (**10f**), with excellent yields in all of the cases (87–99%). Interestingly, the use of different groups such as OH (**10g**) or NH_2_ (**10h**) in the aromatic ring was achieved, but the final obtained yield from **10h** was lower (30%), probably due to the formation of oligomeric species. Pleasingly, the material permitted the use of different halogens, which are sensitive to the reduction, such as Br or Cl, obtaining compounds **11i** and **11j** in good yield. In addition, the use of a heteroaromatic ring (thienyl group) was also tolerated, affording compound **11k** in 87% yield.

**Figure 7:**
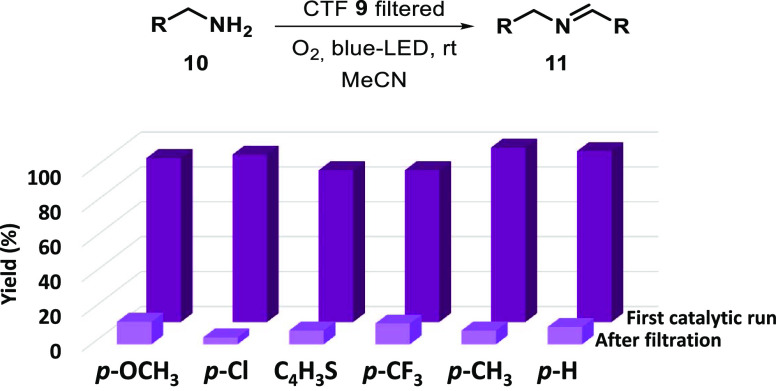
First catalytic run: imine product yield after 14 h under blue LED irradiation before reusing the photocatalytic CTF **9**. After filtration: imine product yield after 14 h under blue LED irradiation after reusing the photocatalytic CTF **9** with each substrate for the second run. All reactions were carried out using **10a**, **10b**, **10c**, **10d**, **10j**, or **10k** (0.2 mmol) and 2 mg of the CTF **9** (only in the first run because it was filtered before the second run) in 2 mL of acetonitrile.

The plausible leaching phenomena were studied with five different amines (**10**) under the catalysis of **9** (Figure 7), containing both electron donor (**10b**, **10c**, **10k**) and electron acceptor groups (**10d**, **10j**). The amount of product generated in the solution after filtration of the material was determined to be very low (4–13%). These data corroborate that the stability of material **9** was maintained regardless of the nucleophilicity of the amine used as a reagent.

**Figure 8:**
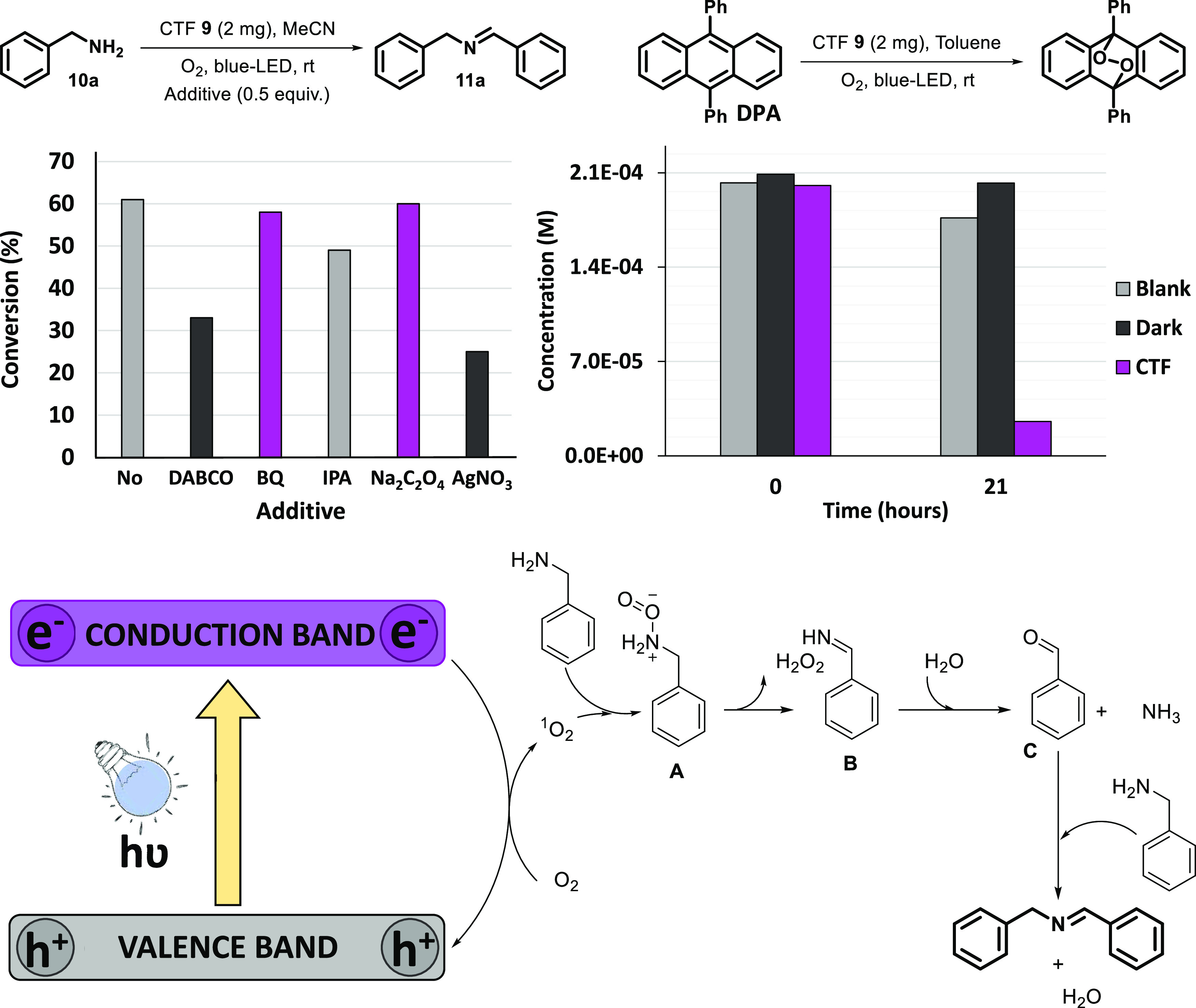
Mechanistic experiments and proposal for the photooxidative coupling of amines with CTF **9**.

Finally, the mechanism of oxidative coupling of amines under the catalysis of material **9** was examined. Generally, two different pathways are possible for this photocatalytic coupling: energy transfer or electron transfer.^6^ A simple way to distinguish between these two mechanisms is the use of selective scavengers (see Table S3 of Supporting Information): DABCO (inhibiting energy transfer processes involving singlet oxygen) and benzoquinone (inhibiting reactions mediated by electron transfer processes, in which the superoxide radical anion is mainly responsible for the transformation).^35^ When the oxidative coupling was carried out in the presence of these additives (Figure 8, top-left), we found that benzoquinone had no effect on the final imine product yield, while the addition of DABCO significantly reduced the reaction outcome. Then, the predominant reactive species probably is singlet oxygen generated from the photoactivation by energy transfer under these reaction conditions. Moreover, an additional experiment was performed to corroborate the formation of singlet oxygen using 9,10-diphenylanthracene (DPA).^36^ The disappearance of DPA was followed by UV–vis spectroscopy measurements, exhibiting a strong decrease in its concentration after 21 h under blue LED irradiation (Figure 8, top-right). The addition of isopropyl alcohol or sodium oxalate as quenchers for hydroxyl radicals or photogenerated holes, respectively, did not affect the catalytic outcome. However, the use of AgNO_3_ as an electron scavenger^37^ decreased the reaction conversion to 25%. Therefore, although the involvement of the superoxo and hydroxyl radicals is avoided, the material could also give rise to electron transfer processes with powerful electron acceptors such as Ag(I). Based on previous reports^6^ and our proofs, we propose the mechanism as an energy transfer process, as shown in Figure 8 (bottom). The excited state of material **9** can generate ^1^O_2_, reacting with **10** to afford a *peroxo* intermediate **A**, which evolves to the corresponding monomeric imine species **B** and hydrogen peroxide (detected by the titanium oxalate method,^38^ see Figure S1 of Supporting Information). Such imine intermediate **B** hydrolyzed to form the aldehyde **C** and ammonia as a byproduct. Finally, the generated aldehyde was condensed with a second equivalent of primary amine to provide the final coupling product **11**.

## 4 Conclusions

In this work, we have studied the stability of photocatalytic organic materials in the presence of strong nucleophiles, such as primary amines, which were employed in the oxidative coupling for the synthesis of imines. To address this issue, we analyzed the distinct chemical erosion that occurred in different materials containing the same photocatalytic phenyl phenothiazine fragment. In particular, the results reported in this study illustrate the very distinct trends shown by imine-, hydrazone-, and triazine-based extended frameworks. Polyimine and polyhydrazone materials are easily degraded by a reaction with the alkyl-amines, hampering, therefore, their use as heterogeneous photocatalysts. However, the triazine framework showed optimal chemical stability under the reaction conditions, together with good catalytic activity. The appropriate design of the PTH-containing triazine framework allowed its use in the photocatalytic oxidative coupling of a variety of amines with good yields of the imine products, keeping a high degree of recyclability of the catalytic material. Comparing the results available in the literature, the performance of our CTF material can be considered in the state of the art. However, the direct comparison should be made with care because many variables considered differ from one work to another (light source, temperature, atmosphere, catalytic loading). In addition, as shown in this work, some results could be reinterpreted as a consequence of leaching and material degradation of poorly stable linkages in the presence of nucleophilic reagents. These findings offer new tools for the design and evaluation of organic photocatalytic materials, avoiding disassembly processes and spurious catalytic results coming from leached homogeneous active fragments.

## Supporting Information Available

Additional experimental details, materials, and methods; spectroscopic characterization of all of the compounds, and benchmarking of catalytic results (PDF)
