# Supplementary material for: Tuning the Activity–Stability Balance of Photocatalytic Organic Materials for Oxidative Coupling Reactions
Source: ACS Appl Mater Interfaces. 2022 Mar 29;14(14):16258–68. doi: 10.1021/acsami.2c01646 (PMC9011354; doi:10.1021/acsami.2c01646)
Supplement: Supplementary file 1 — am2c01646_si_001.pdf [file am2c01646_si_001.pdf]

Supporting Information

Tuning the Activity-Stability Balance of Photocatalytic Organic Materials for Oxidative Coupling Reactions

Alicia Jiménez-Almarza,[a] Alberto López-Magano,[a] Rubén Mas-Ballesté,[a,b,*] José Alemán[b,c,*]

[a] Department of Inorganic Chemistry (Module 7), Facultad de Ciencias, Universidad Autónoma de Madrid, 28049-Madrid, Spain.

[b] Institute for Advanced Research in Chemical Sciences (IAdChem), Universidad Autónoma de Madrid, 28049-Madrid, Spain.

[c] Department of Organic Chemistry (Module 1), Facultad de Ciencias, Universidad Autónoma de Madrid, 28049-Madrid, Spain.

e-mail: jose.aleman@uam.es; ruben.mas@uam.es

Table of Contents

1. Materials and general methods 3

2. Characterization of building blocks 2 – 6. 5

3. Optical properties of 4 and 5 11

4. Characterization of products of oxidative coupling of amines. 12

5. Mechanistic experiments 27

6. Leaching test 29

7. Recyclability test 31

8. Calculation of chemical erosion 33

9. FT-IR Spectroscopy: Attenuated Total Reflectance (ATR) 38

10. Diffuse Reflectance Spectroscopy and Fluorescence 40

11. ^{13}C Nuclear Magnetic Resonance of Solids 42

12. BET Isotherms 45

13. Scanning Electron Microscopy 48

14. Thermogravimetric analysis 49

15. Electrochemistry 50

16. Determination of Energy Levels 52

17. Benchmarking of catalytic results 53

18. References 54

1. Materials and general methods

All reagents and solvents were purchased from commercial sources and used without further purification. Building blocks employed were purchased from commercial sources except for 10-(4-formylphenyl)-10*H*-phenothiazine-3,7-dicarbaldehyde (**4**) and 4,4'-(10-(4'-cyano-[1,1'-biphenyl]-4-yl)-10*H*-phenothiazine-3,7-diyl)dibenzonitrile (**5**) that they were synthesized in three steps, each one. In the case of 2,5-diethoxyterephthalohydrazide (**6b**), two steps were needed for its synthesis.

Light irradiation in the oxidative coupling of primary amines was carried out using a 15 W blue LED photoreactor thermostated at 25 ˚C. A spectro-radiometer equipment *Stellarnet* model *Blue-Wave UV-NB50* was employed to measure the emission of the blue LED used (range 300-600 ʎ, integration time CR2-AP + 200 ms, intensity 21.7217 W/m^2).

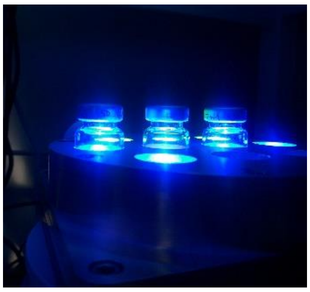

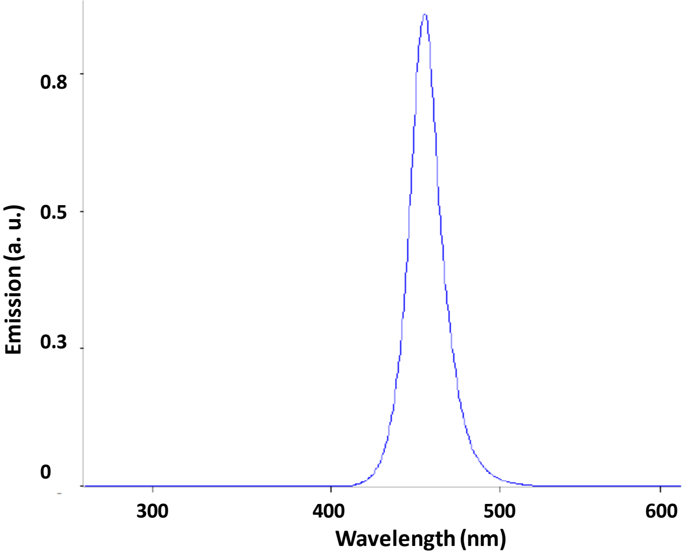

Nuclear Magnetic Resonance (NMR) spectra were acquired on a *Bruker AV-300 spectrometer*, running at 300 MHz for ^{1}H and at 75 MHz for ^{13}C. Chemical shift (δ) are reported in ppm relative to residual solvent signals ($CDCl_3$: 7.26 ppm for ^{1}H-NMR, 77.0 ppm for ^{13}C-NMR; DMSO: 2.50 ppm for ^{1}H-NMR, 39.5 ppm for ^{13}C-NMR). ^{13}C solid State Nuclear Magnetic Resonance were acquired on a *Bruker AV-400 spectrometer* coupled to a multinuclear probe (^{15}N-^{31}P) CPMAS with triple channel (BL4 X/Y/^{1}H) for a 4 mm rotor at room temperature, using 1k scans and 12 kHz of turning speed. The ^{1}H excitation pulse used is $\pi/2$ * 2.75 μs and the contact pulse is 3 ms.

Elemental Chemical Analysis were obtained by *Servicio Interdepartamental de Apoyo a la Investigación* in UAM, in an elemental analyzer *LECO CHNS-932* model number 601-800-500.

IR spectra were obtained in a *Perkin-Elmer 283* equipped with *ATR MIRacle Single Reflection Horizontal*.

Diffuse Reflectance was measured in a UV/Vis spectrophotometer by UNICAM. Emission intensities were recorded using the *FS5 Spectrofluorometer* by Edinburgh Instrument with an operating software by Fluoracle®. The fluorescence emission of materials was measured irradiating the sample at 450 nm.

Volumetric N_2 sorption isotherms were collected at 77 K (N_2) using an ASAP 2460 HD (Micromeritics). Temperature was controlled by using a liquid nitrogen bath.

Scanning Electron Microscopy (SEM) images were carried out on a *JEOL JSM-7600F Field Emission Scanning Electron Microscope* with a field emission cannon and an elemental analysis system EDS and electron “in lens”. The images were obtained in vacuum after being metallized in a *Sputter Quórum Q150T-S* with gold coating.

Thermogravimetric analyses (TGA) were performed on a TGA Q500 Thermobalance from TA instruments, heating the sample from 25 ^{o}C to 900 ^{o}C at 10 ^{o}C/min under air atmosphere.

2. Characterization of building blocks 2 – 6.

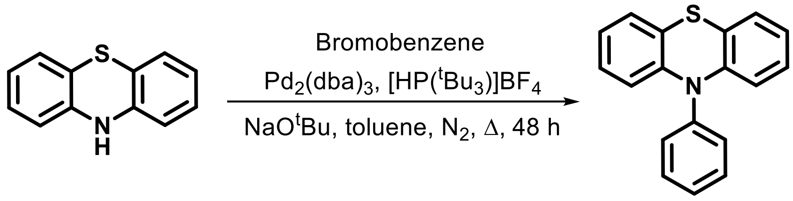

1 **2**

The NMR spectra of **2** were compared with previous reported works.[1]

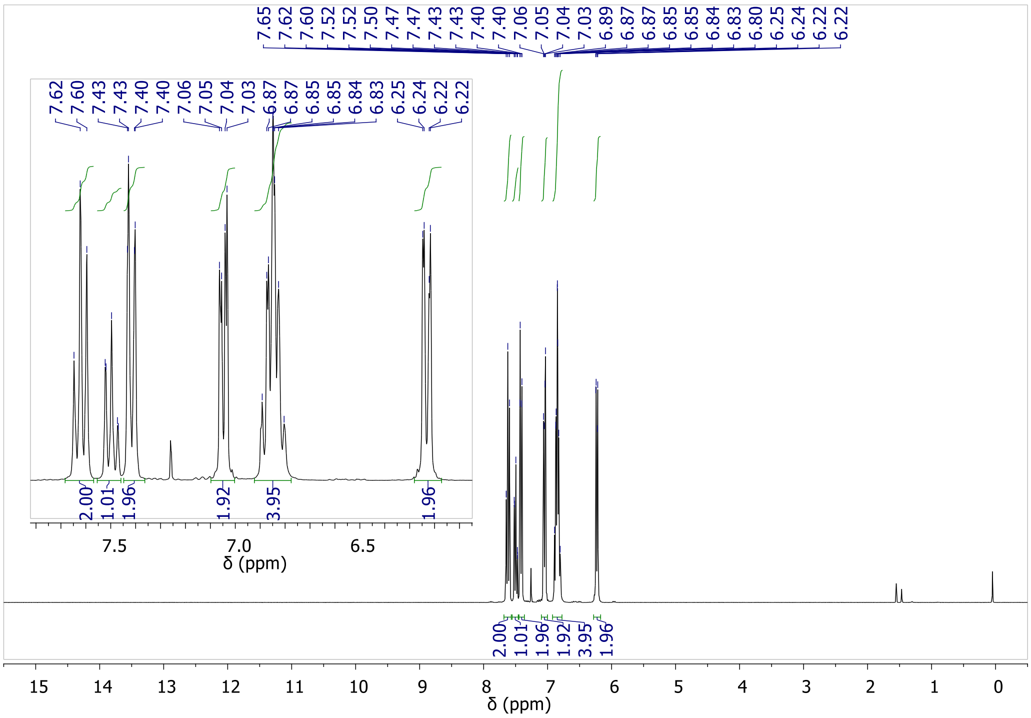

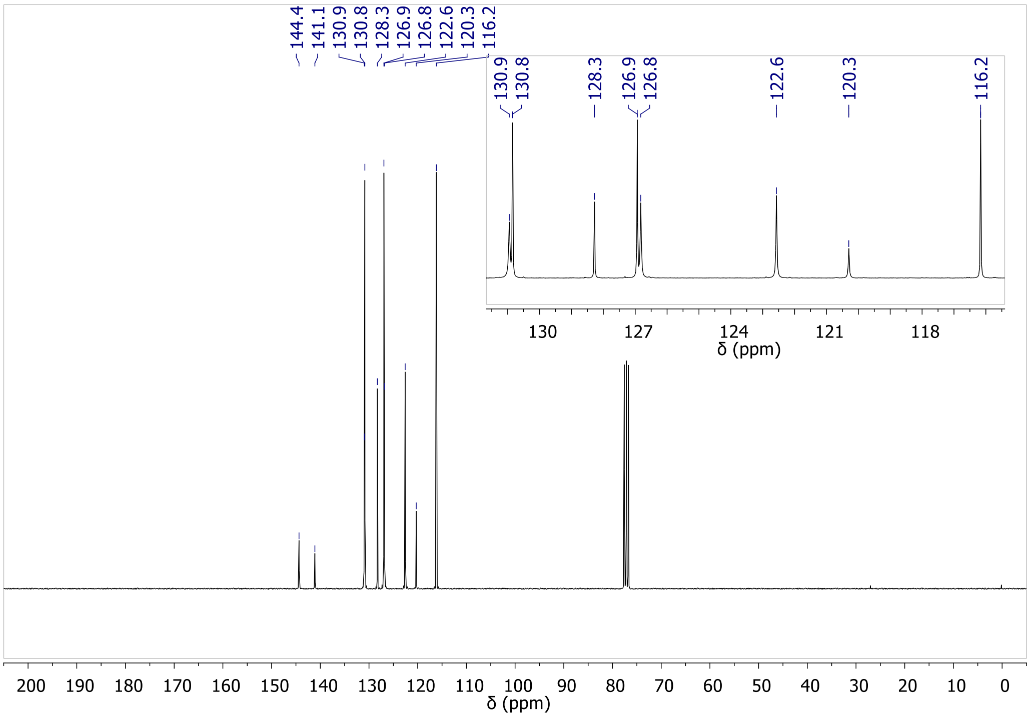

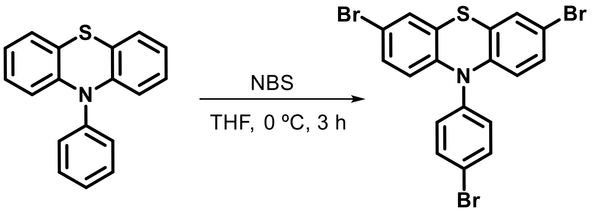

2 **3**

The NMR spectra of **3** were compared with previous reported works.[1]

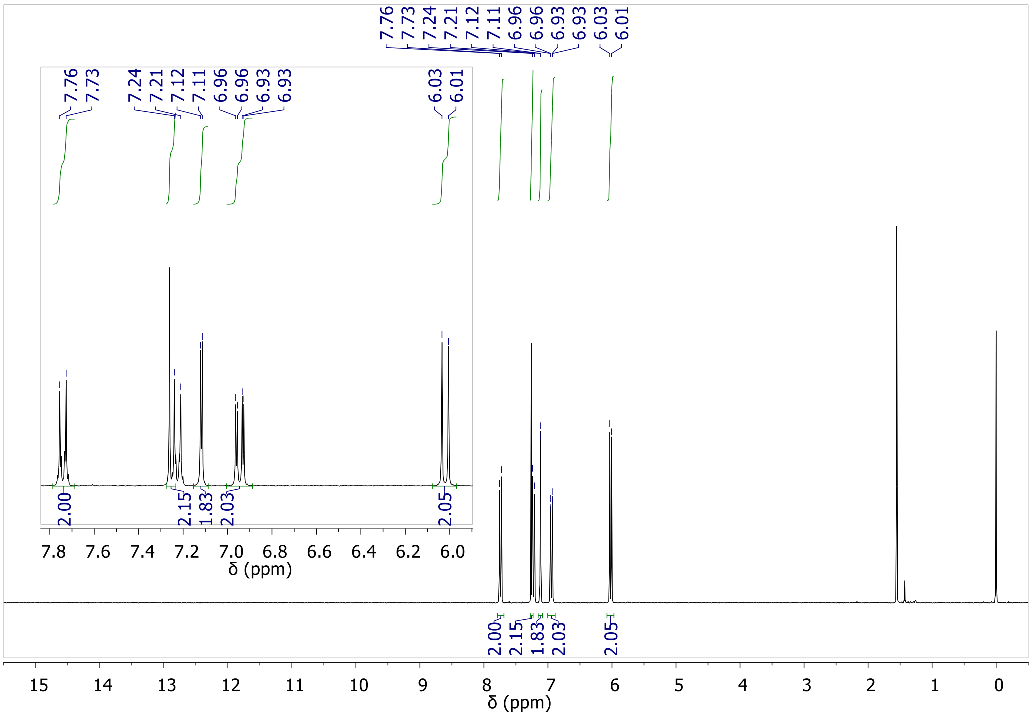

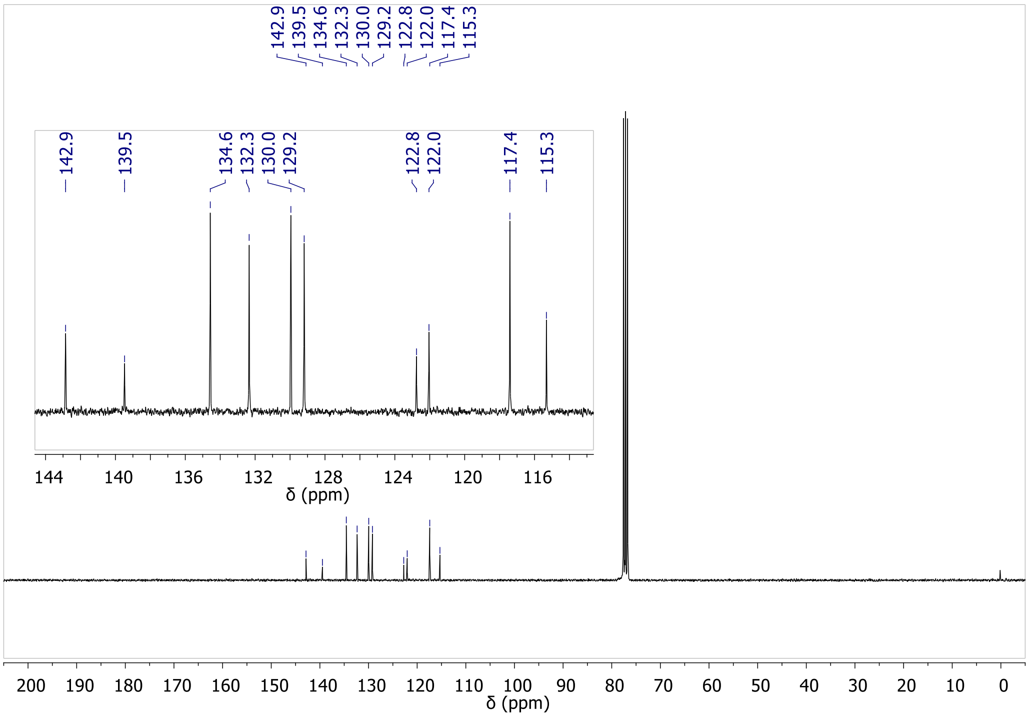

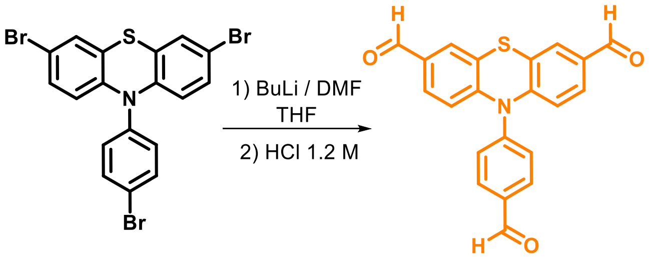

The NMR spectra of **4** were compared with previous reported works.[1]

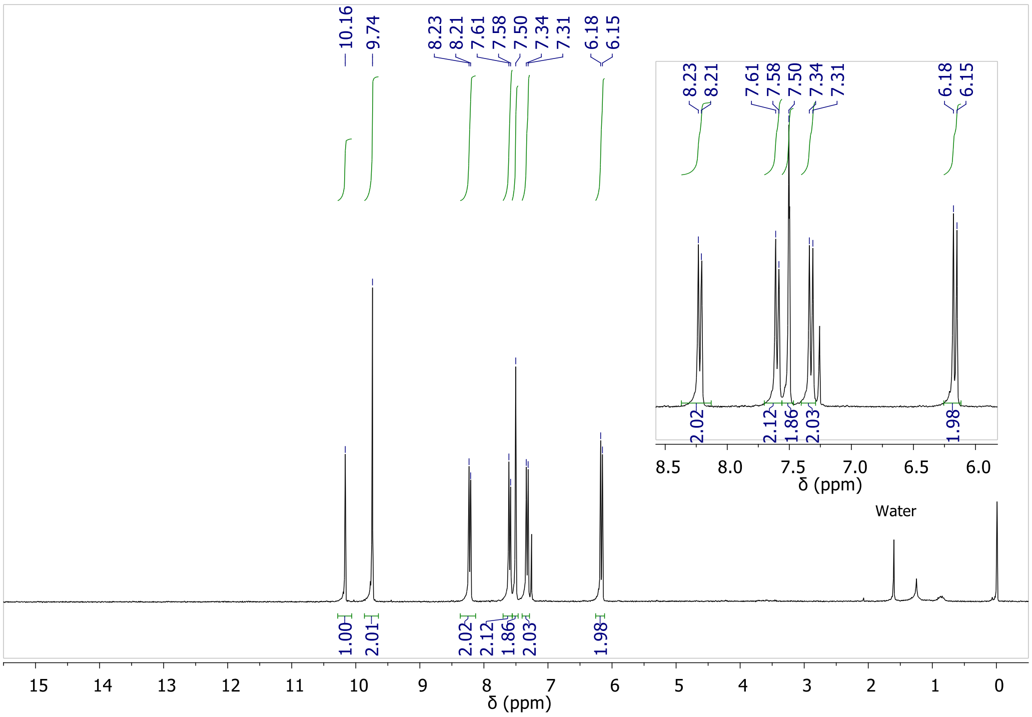

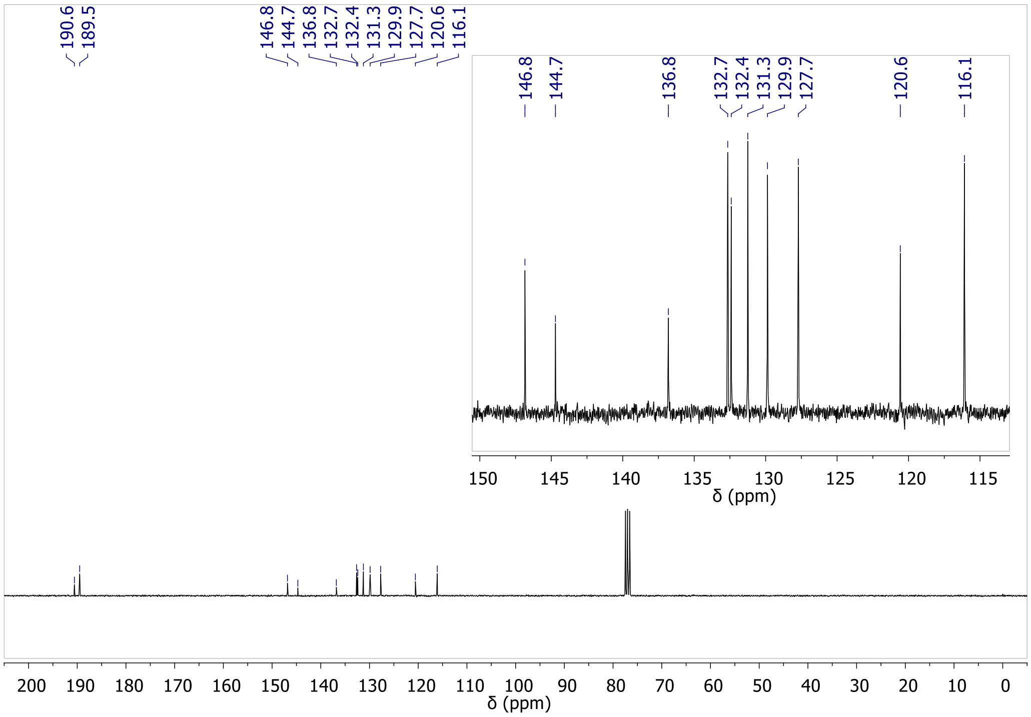

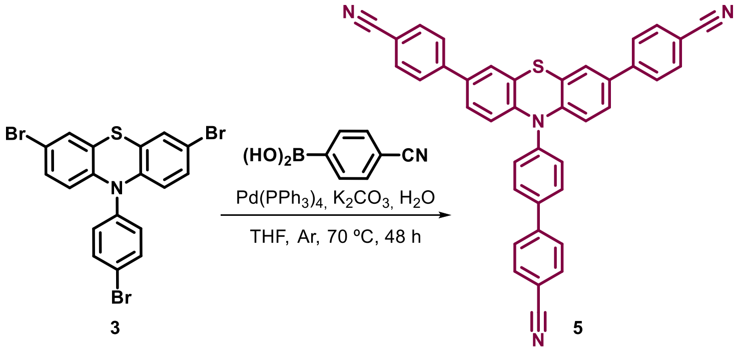
Br
S
Br
N
Br
(HO)$_2$B–C$_6$H$_4$–CN
Pd(PPh$_3$)$_4$, K$_2$CO$_3$, H$_2$O
THF, Ar, 70 ºC, 48 h
N
S
N
N
N

3 5

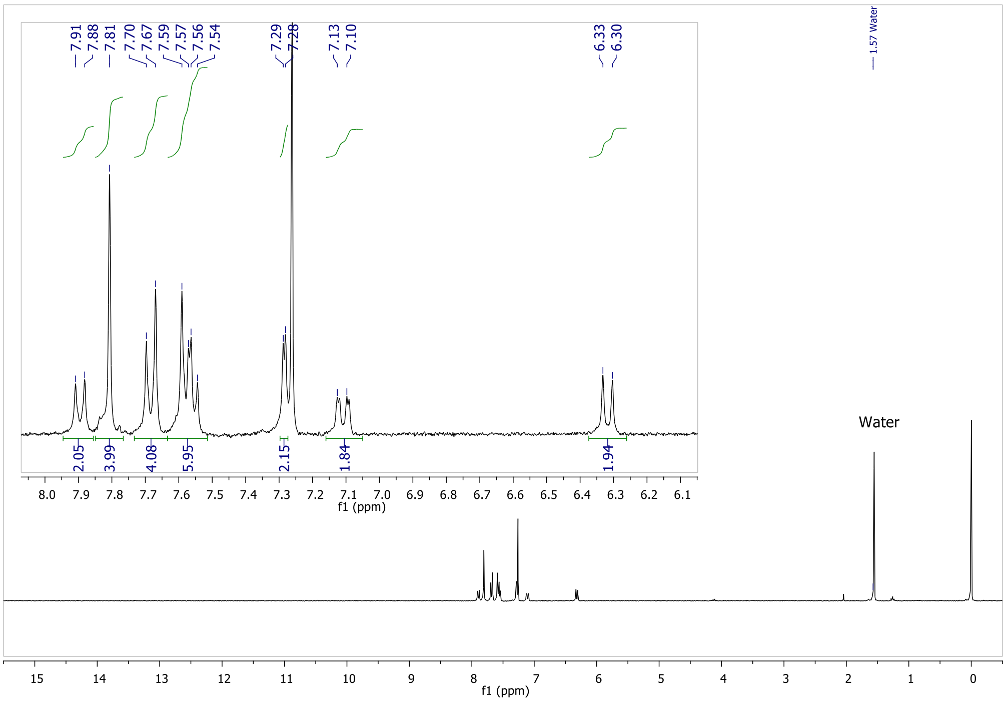
7.91
7.88
7.81
7.70
7.67
7.59
7.57
7.56
7.54
7.29
7.28
7.13
7.10
6.33
6.30
1.57 Water
2.05
3.99
4.08
5.95
2.15
1.84
1.94
Water
f1 (ppm)
f1 (ppm)

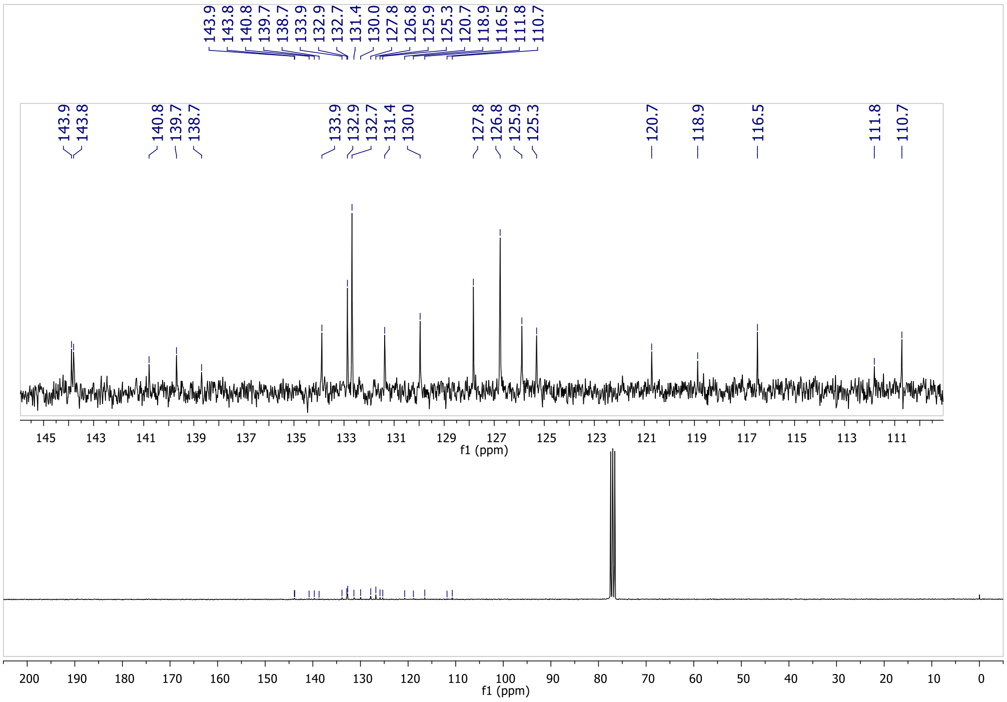
143.9
143.8
140.8
139.7
138.7
133.9
132.9
132.7
131.4
130.0
127.8
126.8
125.9
125.3
120.7
118.9
116.5
111.8
110.7
f1 (ppm)
f1 (ppm)

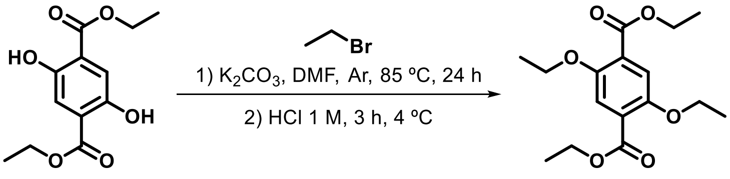

6 **6a**

The NMR spectra of **6a** were compared with previous reported works.[2]

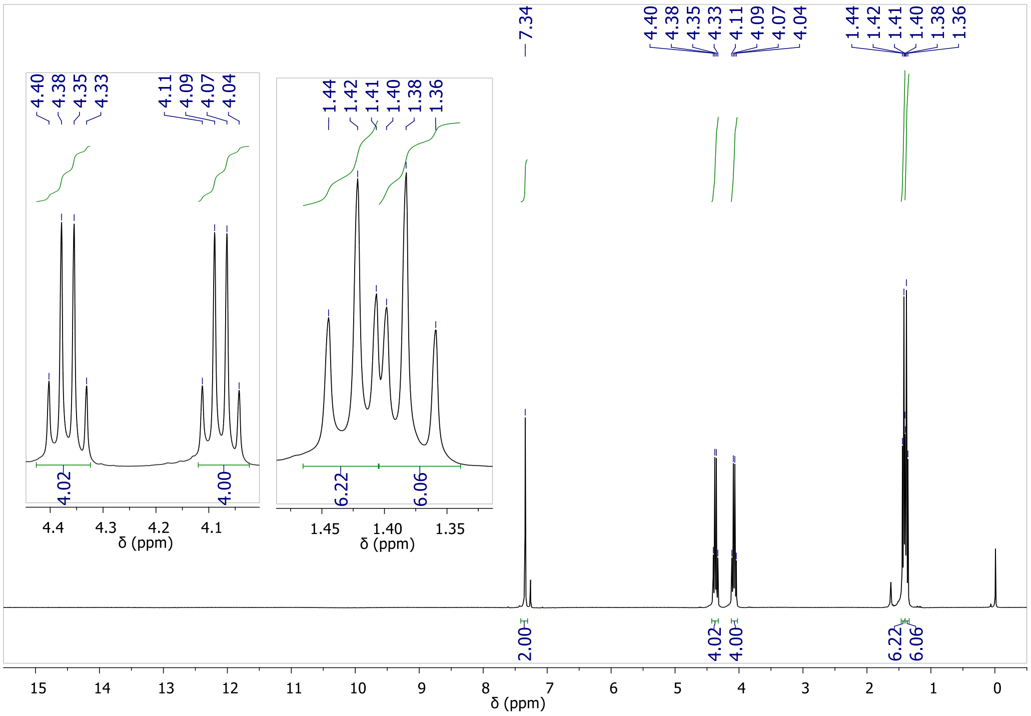

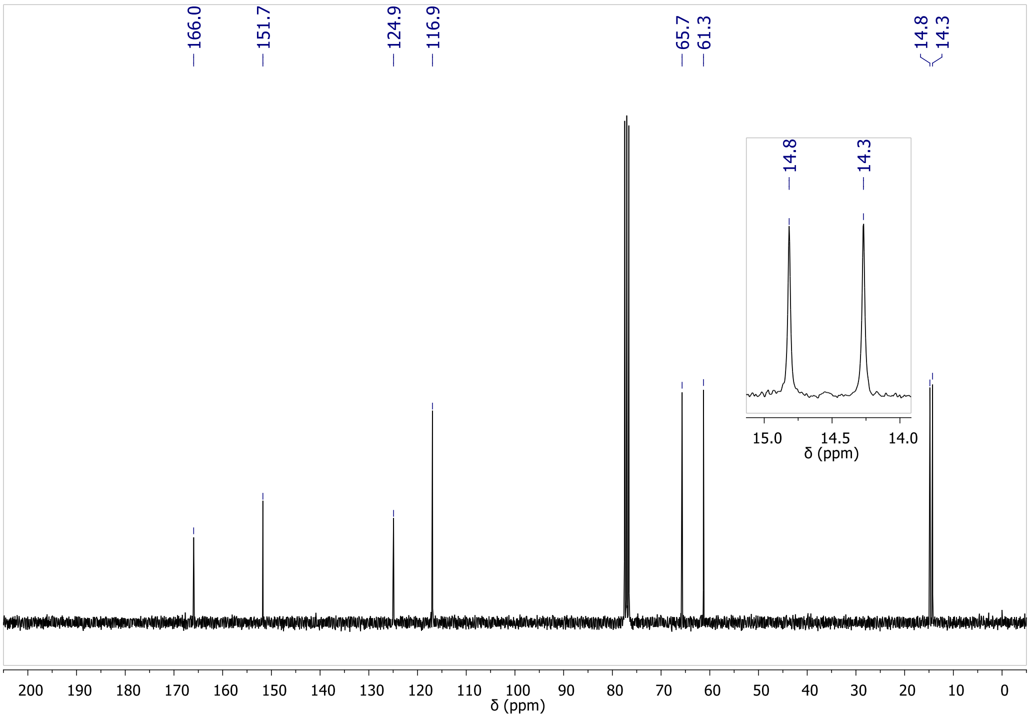

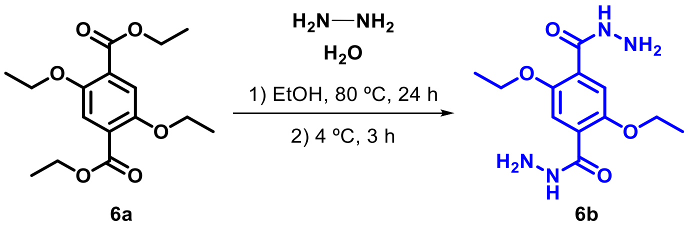

The NMR spectra of **6b** were compared with previous reported works.[2]

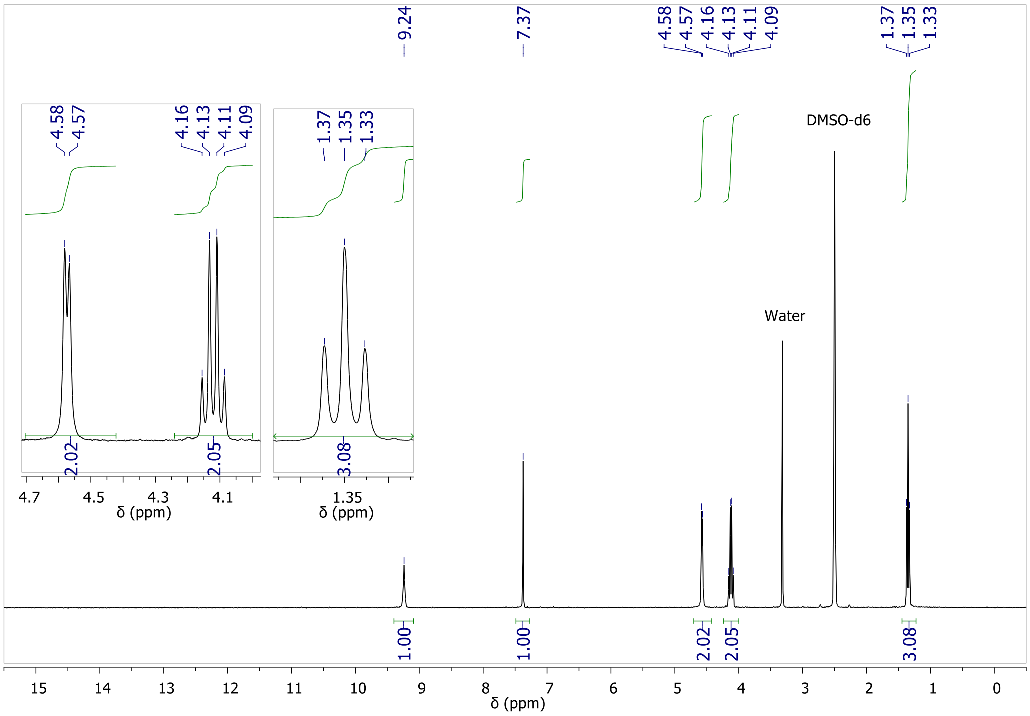

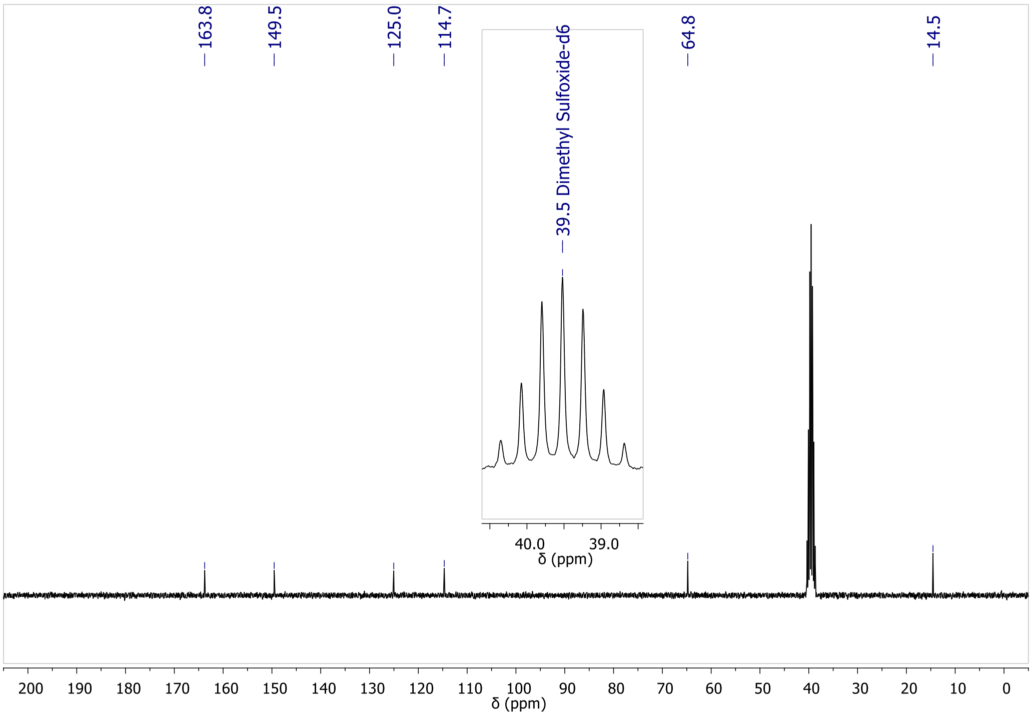

3. Optical properties of 4 and 5

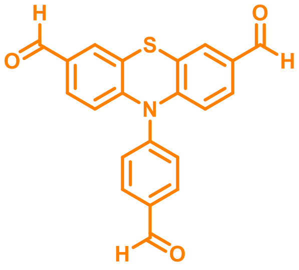

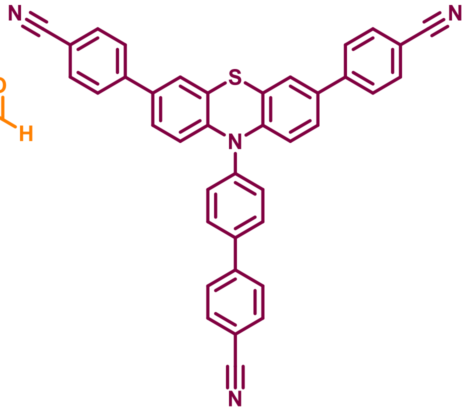

4 **5**

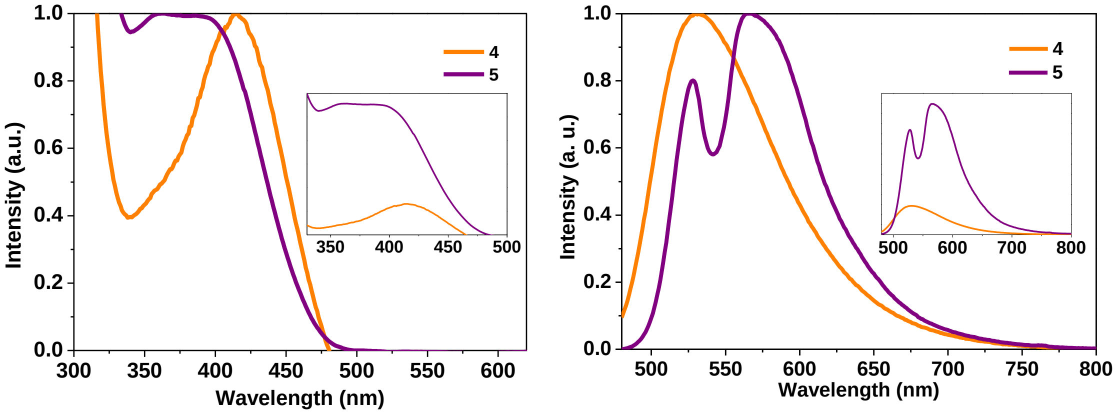

**4:**	415 nm	**4:**	531 nm
**5:**	359, 396 nm	**5:**	528, 565 nm

A solution in acetonitrile (25 mL) of **4** and **5** (0.01 mM) was prepared from a solution in acetonitrile (100 mL) of **4** and **5** (0.1 mM). The absorption (left) and emission (right) spectra were obtained by the UV-Vis spectrophotometer and the spectrofluorometer irradiating at 450 nm, respectively.

4. Characterization of products of oxidative coupling of amines.

Reaction optimization:

An oven-dried 10 mL vial equipped with a magnetic stir bar was charged with x mg of material (**7**, **8** or **9**), 1,3,5-trimethoxybenzene (8.4 mg, 0.05 mmol) as standard for quantitative NMR and 2 mL of solvent. Then, the amine **10a** (10.9 µL, 0.1 mmol) was added. The vial was closed with a PTFE / rubber septum and oxygen was bubbled in the reaction mixture for 5 minutes. The reaction mixture with an O_2 balloon was stirred under light irradiation at 25 ˚C. The reaction yield was determined by ^{1}H-NMR using the quantitative standard.

Table S1. Optimization of the oxidative coupling of amines (**10**) to imines (**11**) under light irradiation and CTF **9**[a]

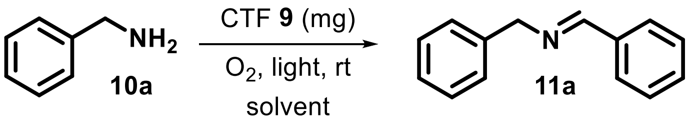

Entry	Solvent (mL)	Light (nm)	Photocatalyst (mg)	Time (h)	**10a** (mmol)	Yield (%)[b]
1	MeCN	450	1	2	0.1	40
2	EtOH	450	1	2	0.1	10
3	DCM	450	1	2	0.1	25
4	Toluene	450	1	2	0.1	3
5	DMSO	450	1	2	0.1	0
6	MeCN	450	1	2	0.1	30[c]
7	MeCN	450	1	2	0.1	4[d]
8	MeCN	450	1	8	0.1	85
9	MeCN	450	1	24	0.1	100
10	MeCN	450	No	24	0.1	0
11	MeCN	450	1	24	0.2	77
12	MeCN	540	1	2	0.1	4
13	MeCN	365	1	2	0.1	59
14	MeCN	White	1	2	0.1	10
15	MeCN	450	0.5	4.5	0.1	27
16	MeCN	450	1	4.5	0.1	64
17	MeCN	450	2	4.5	0.1	100
18	MeCN	450	2	2	0.1	67
19	MeCN	365	2	2	0.1	75
20	MeCN	450	3	2	0.1	73
21	MeCN	450	2	1	0.1	37
22	MeCN	450	2	3	0.1	97
23	MeCN	450	2	4	0.1	100
24	MeCN	450	2	4	0.2	82
25	MeCN	450	2	24	0.5	65
26	MeCN	450	2	14	0.2	100
27	MeCN	450	2	14	0.2	41[c]
28	MeCN	450	2	14	0.2	0[d]
29	MeCN	450	No	14	0.2	0
30	MeCN	Dark	2	14	0.2	0

[a] All reactions were carried out using **10a** and CTF **9** as photocatalyst in 2 mL of solvent under light irradiation. [b] Determined by ^{1}H-NMR with 1,3,5-trimethoxybenzene as quantitative standard. [c] Reaction carried out open to air. [d] Reaction carried out under inert atmosphere. The vial was closed with a rubber septum and the reaction mixture was degassed by three cycles vacuum / argon of "freeze-pump-thaw".

Table S2. Oxidative coupling of amines (**10**) to imines (**11**) under blue-LED irradiation and CTF **9**[a]

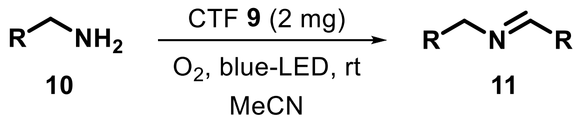

Entry	R	Photocatalyst (mg)	Time (h)	Imine	Yield (%)[b]
1	C_6H_5	**9**	14	**11a**	98
2	C_6H_5	-	14	**11a**	0
3	*p*-OCH_3-C_6H_4	**9**	14	**11b**	94
4	*p*-OCH_3-C_6H_4	-	14	**11b**	0
5	*p*-CH_3-C_6H_4	**9**	14	**11c**	100
6	*p*-CH_3-C_6H_4	-	14	**11c**	0
7	*p*-CF_3-C_6H_4	**9**	14	**11d**	87
8	*p*-CF_3-C_6H_4	-	14	**11d**	0
9	(*m*-CF_3)$_2$-C_6H_3	**9**	14	**11e**	76
10	(*m*-CF_3)$_2$-C_6H_3	-	14	**11e**	0
11	*p*-CN-C_6H_4	9	14	**11f**	77
12	*p*-CN-C_6H_4	-	14	**11f**	0
13	*o*-OH-C_6H_4	**9**	14	**11g**	91
14	*o*-OH-C_6H_4	-	14	**11g**	0
15	*p*-NH_2-C_6H_4	**9**	14	**11h**	30
16	*p*-NH_2-C_6H_4	-	14	**11h**	0
17	*p*-Br- C_6H_4	**9**	14	**11i**	99
18	*p*-Br- C_6H_4	-	14	**11i**	0
19	*p*-Cl- C_6H_4	**9**	14	**11j**	96
20	*p*-Cl- C_6H_4	-	14	**11j**	0
21	2-Thienyl	**9**	14	**11k**	87
22	2-Thienyl	-	14	**11k**	0

[a] All reactions were carried out using **10** (0.2 mmol) and 2 mg of CTF **9** as photocatalyst in 2 mL of acetonitrile under blue-LED irradiation during 14 h. [b] Determined by ^{1}H-NMR with 1,3,5-trimethoxybenzene (0.1 mmol) as quantitative standard.

N-benzyl-1-phenylmethanimine (11a) [3–6]

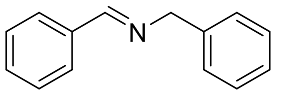

No purification was needed (the crude mixture was filtered off, and the solvent was evaporated); 98 % yield; yellow oil. ^{1}H-NMR (300 MHz, $CDCl_3$) δ 8.41 (s, 1H), 7.79 (dd, *J* = 6.5, 3.2 Hz, 2H), 7.42 (dd, *J* = 5.0, 1.8 Hz, 3H), 7.35 (d, *J* = 4.4 Hz, 4H), 7.28 (dd, *J* = 4.9, 3.9 Hz, 1H), 4.84 (s, 2H) ppm. ^{13}C-NMR (75 MHz, $CDCl_3$) δ 162.0, 139.3, 136.2, 130.8, 128.6, 128.5, 128.3, 128.0, 127.0, 65.1 ppm.

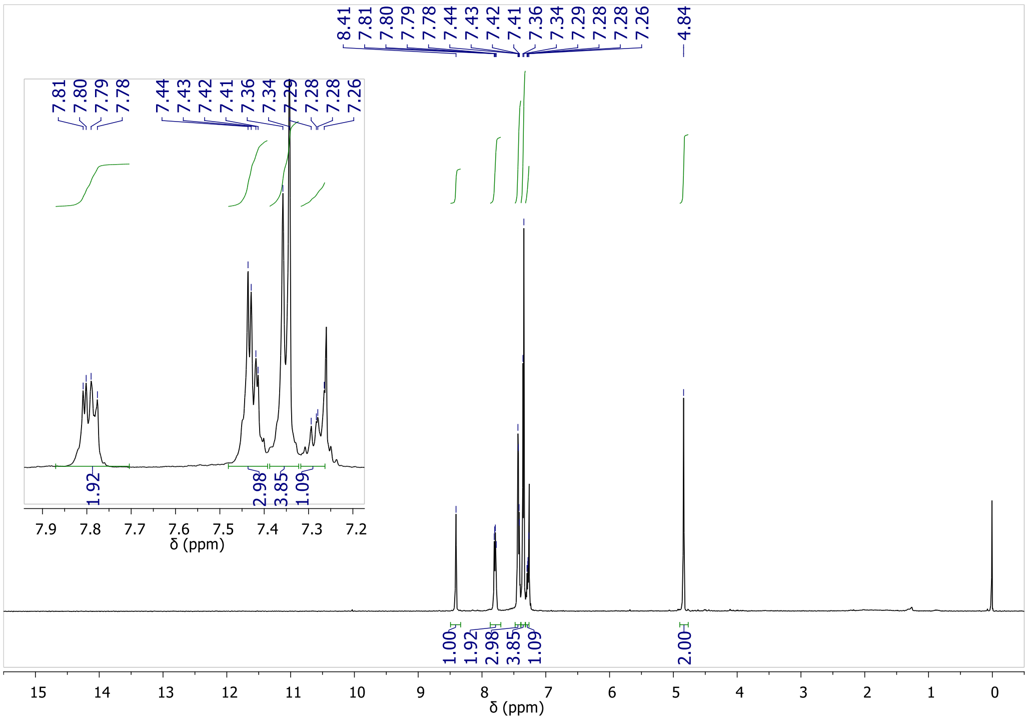

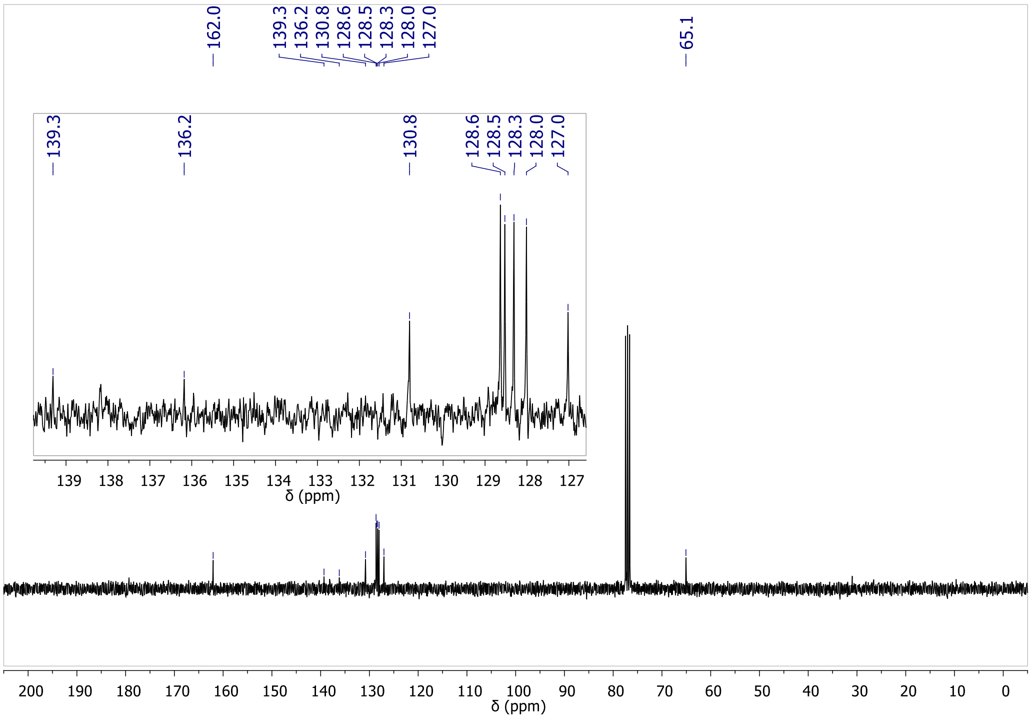

_N_-(4-methoxybenzyl)-1-(4-methoxyphenyl)methanimine (11b) [3–6]

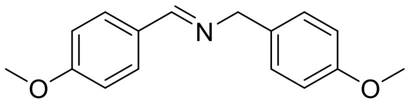

No purification was needed (the crude mixture was filtered off, and the solvent was evaporated); 94 % yield; yellow oil. ^{1}H-NMR (300 MHz, $CDCl_3$) δ 8.24 (s, 1H), 7.63 (d, *J* = 8.8 Hz, 2H), 7.16 (d, *J* = 8.6 Hz, 2H), 6.85 (d, *J* = 8.8 Hz, 2H), 6.79 (d, *J* = 8.7 Hz, 2H), 4.63 (s, 2H), 3.76 (s, 3H), 3.72 (s, 3H) ppm. ^{13}C-NMR (75 MHz, $CDCl_3$) δ 161.2, 132.0, 131.5, 129.9, 129.2, 129.0, 114.3, 114.0, 113.9, 64.3, 55.4, 55.3 ppm.

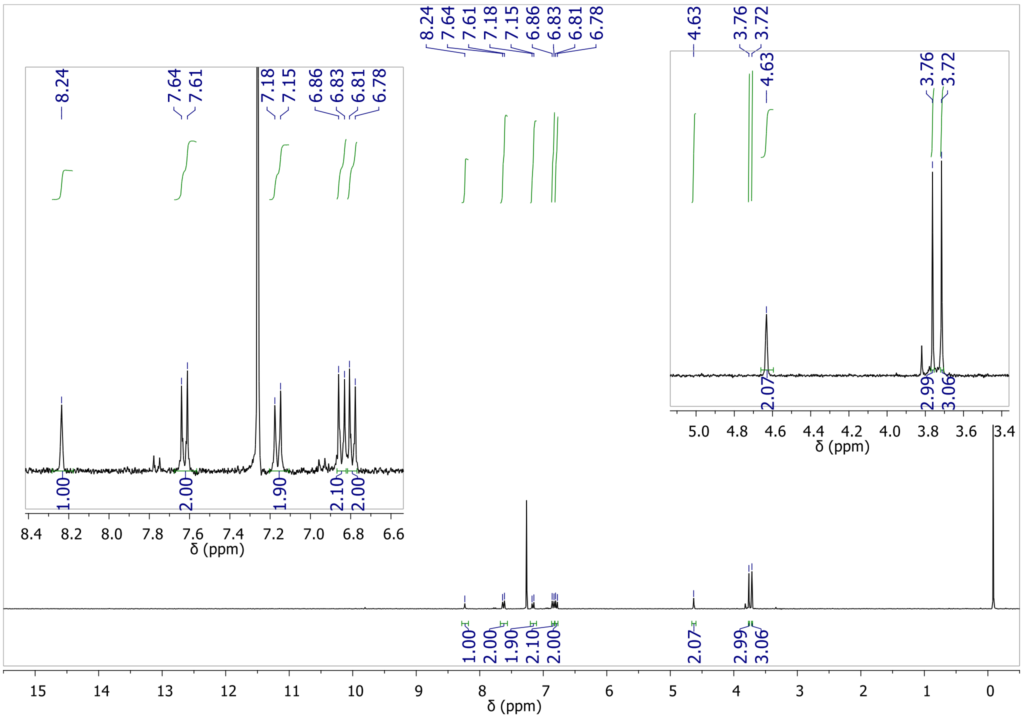

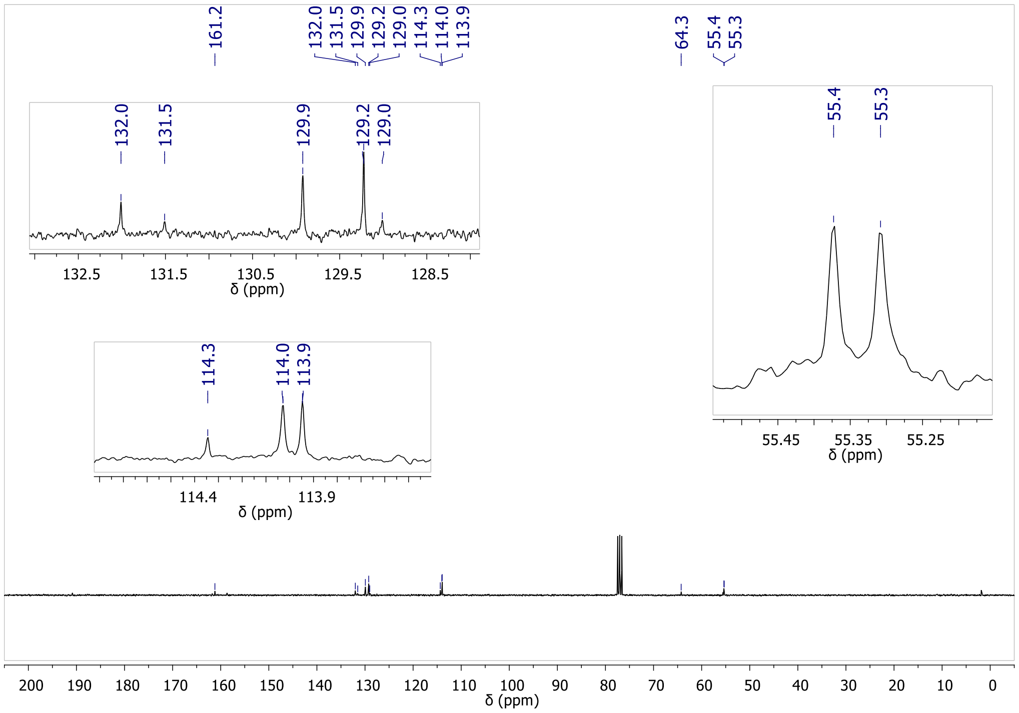

N-(4-methylbenzyl)-1-(p-tolyl)methanimine (11c) [3–6]

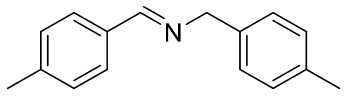

No purification was needed (the crude mixture was filtered off, and the solvent was evaporated); 100 % yield; yellow solid. ^{1}H-NMR (300 MHz, $CDCl_3$) δ 8.34 (s, 1H), 7.66 (d, *J* = 8.1 Hz, 2H), 7.22 (d, *J* = 6.3 Hz, 4H), 7.14 (d, *J* = 8.2 Hz, 2H), 4.77 (s, 2H), 2.38 (s, 3H), 2.33 (s, 3H) ppm. ^{13}C-NMR (75 MHz, $CDCl_3$) δ 161.7, 141.0, 136.5, 136.4, 133.6, 129.3, 129.2, 128.3, 128.0, 64.8, 21.5, 21.1 ppm.

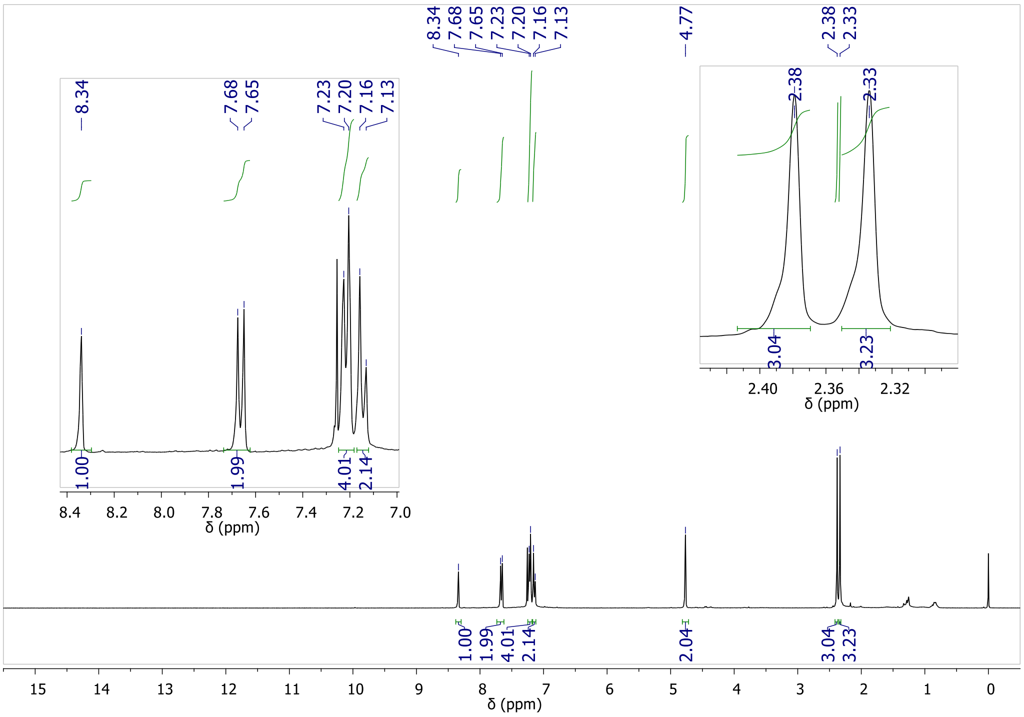

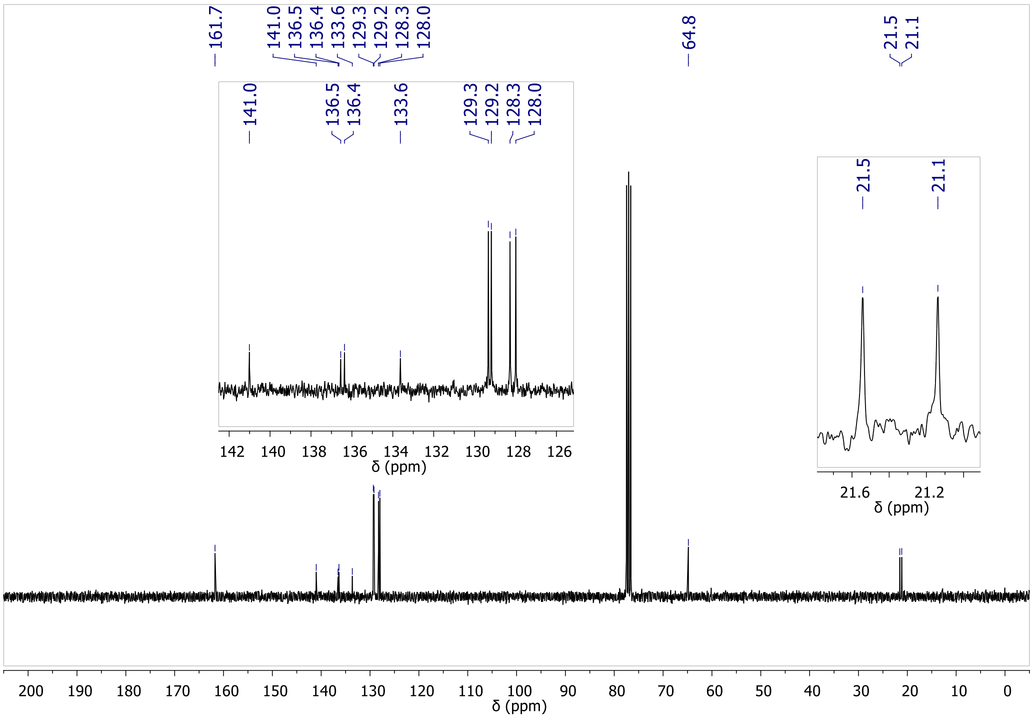

N-(4-(trifluoromethyl)benzyl)-1-(4-(trifluoromethyl)phenyl)methanimine (11d) [3–6]

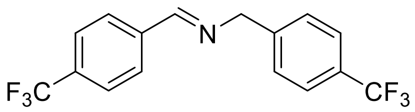

No purification was needed (the crude mixture was filtered off, and the solvent was evaporated); 87 % yield; white solid. ^{1}H-NMR (300 MHz, $CDCl_3$) δ 8.47 (s, 1H), 7.91 (d, *J* = 8.1 Hz, 2H), 7,69 (d, *J* = 8.2 Hz, 2H), 7.62 (d, *J* = 8.2 Hz, 2H), 7.48 (d, *J* = 8.1 Hz, 2H), 4.90 (s, 2H) ppm. ^{13}C-NMR (75 MHz, $CDCl_3$) δ 161.4, 143.0, 139.0, 132.7 (q, *J* = 32.6 Hz), 129.6 (q, *J* = 32.6 Hz), 128.7, 128.3, 125.8 (q, *J* = 3.8 Hz), 125.6 (q, *J* = 3.8 Hz), 122.4 (q, *J* = 25.2 Hz), 118.8 (q, *J* = 25.2 Hz), 64.5 ppm. ^{19}F-NMR (282 MHz, $CDCl_3$) δ -62.47, -62.85.

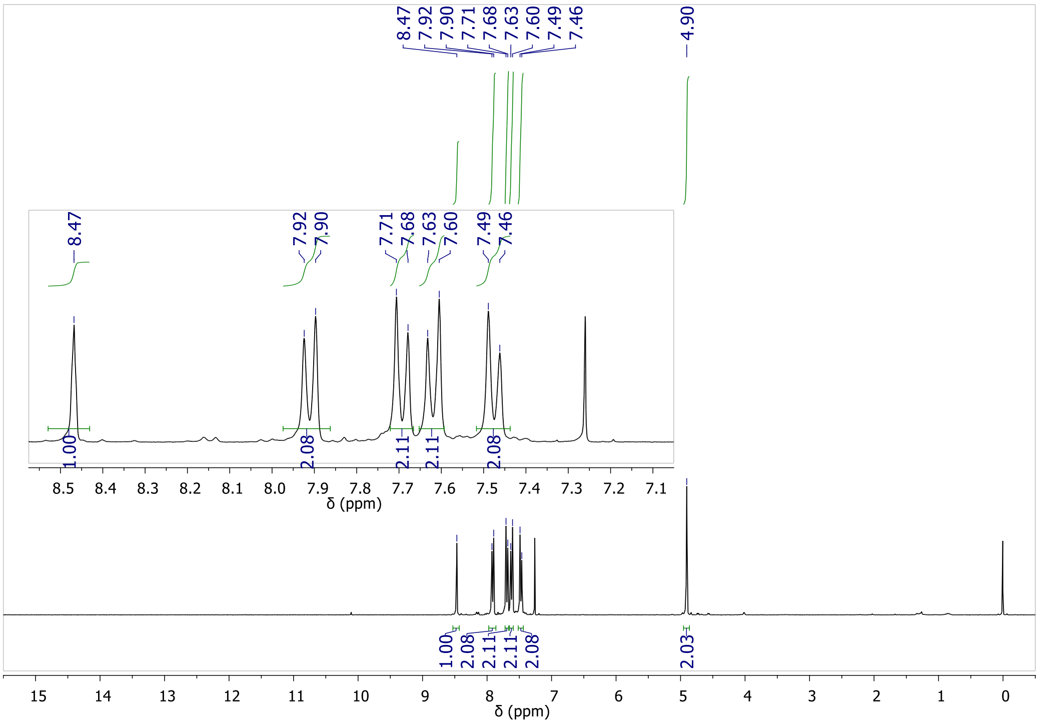

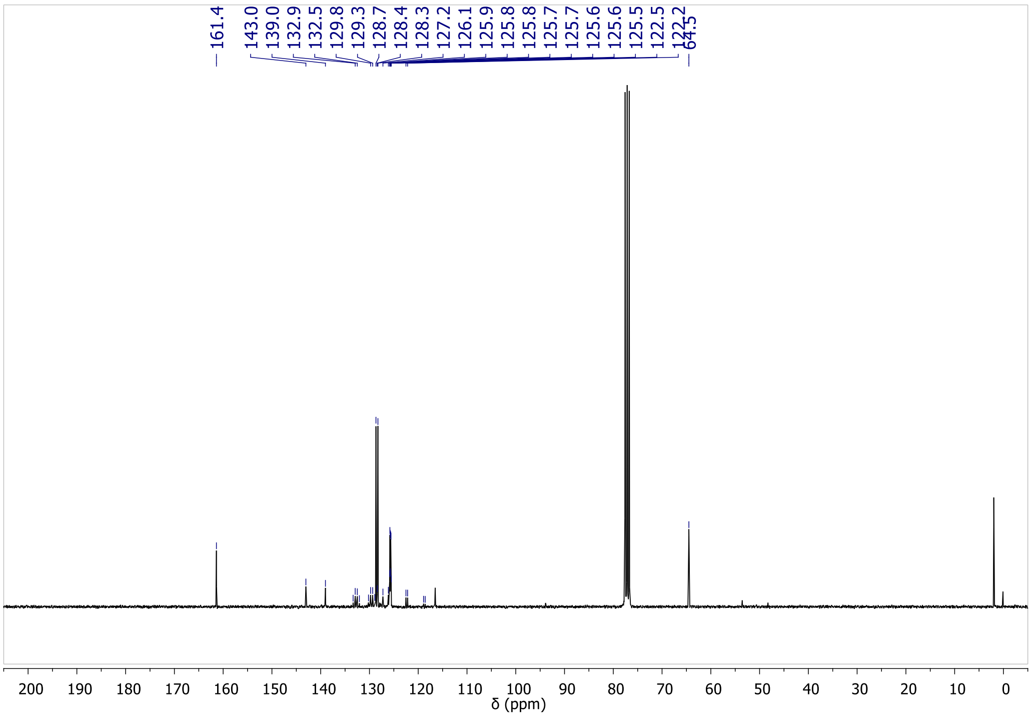

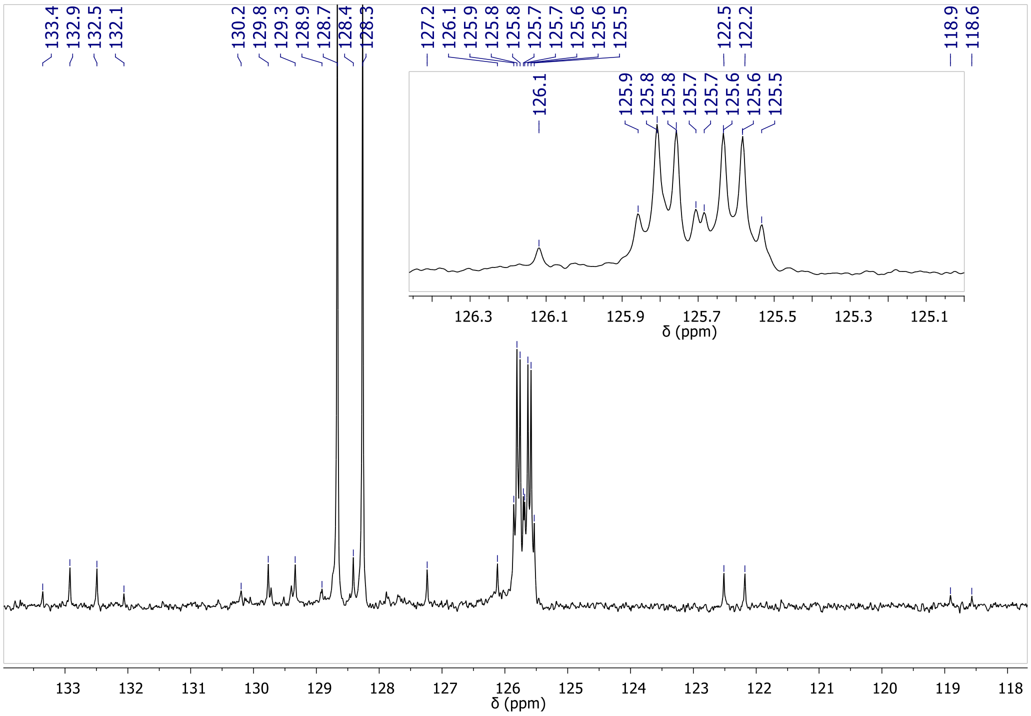
133.4
132.9
132.5
132.1
130.2
129.8
129.3
128.9
128.7
128.4
128.3
127.2
126.1
125.9
125.8
125.8
125.7
125.7
125.6
125.6
125.5
122.5
122.2
118.9
118.6
δ (ppm)

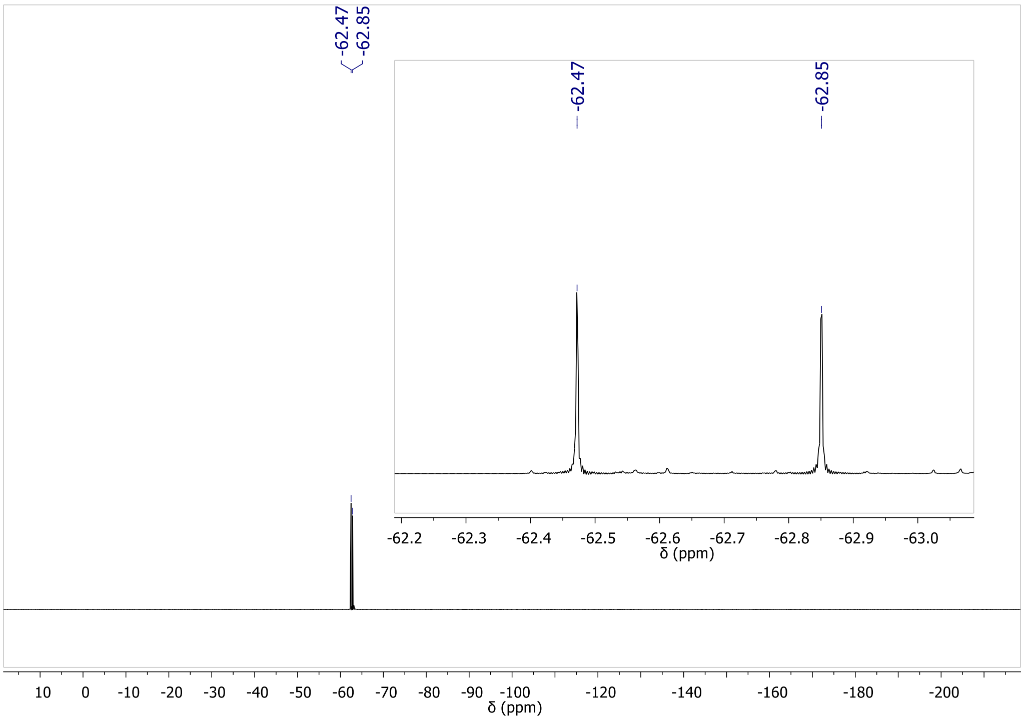
-62.47
-62.85
δ (ppm)

N-(3,5-bis(trifluoromethyl)benzyl)-1-(3,5-bis(trifluoromethyl)phenyl)methanimine (11e) [3–6]

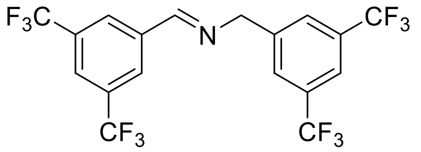

No purification was needed (the crude mixture was filtered off, and the solvent was evaporated); 76 % yield; white solid. ^{1}H-NMR (300 MHz, $CDCl_3$) δ 8.55 (s, 1H), 8.25 (s, 2H), 7.97 (s, 1H), 7.83 (s, 3H), 4.97 (s, 2H) ppm. ^{13}C-NMR (75 MHz, $CDCl_3$) δ 160.2, 141.3, 137.6, 132.8-131.0 (m), 128.3-128.2 (m), 128.2-128.1 (m), 125.1, 124.9, 124.4-124.3 (m), 121.5, 121.3-121.1 (m), 63.6 ppm. ^{19}F-NMR (282 MHz, $CDCl_3$) δ -62.86, -63.01.

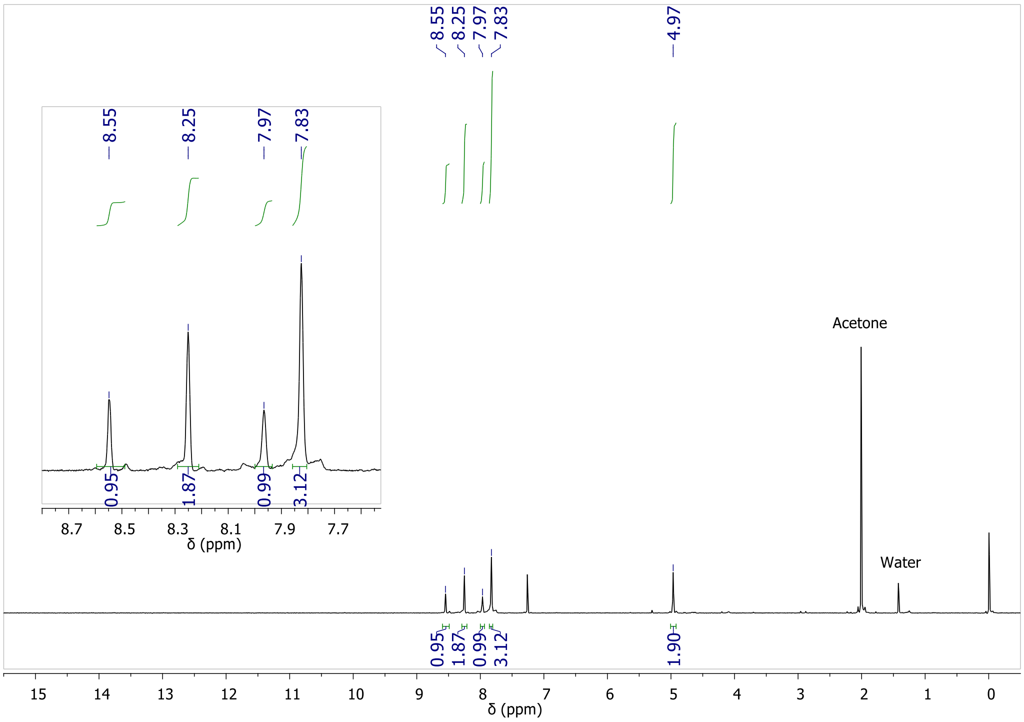

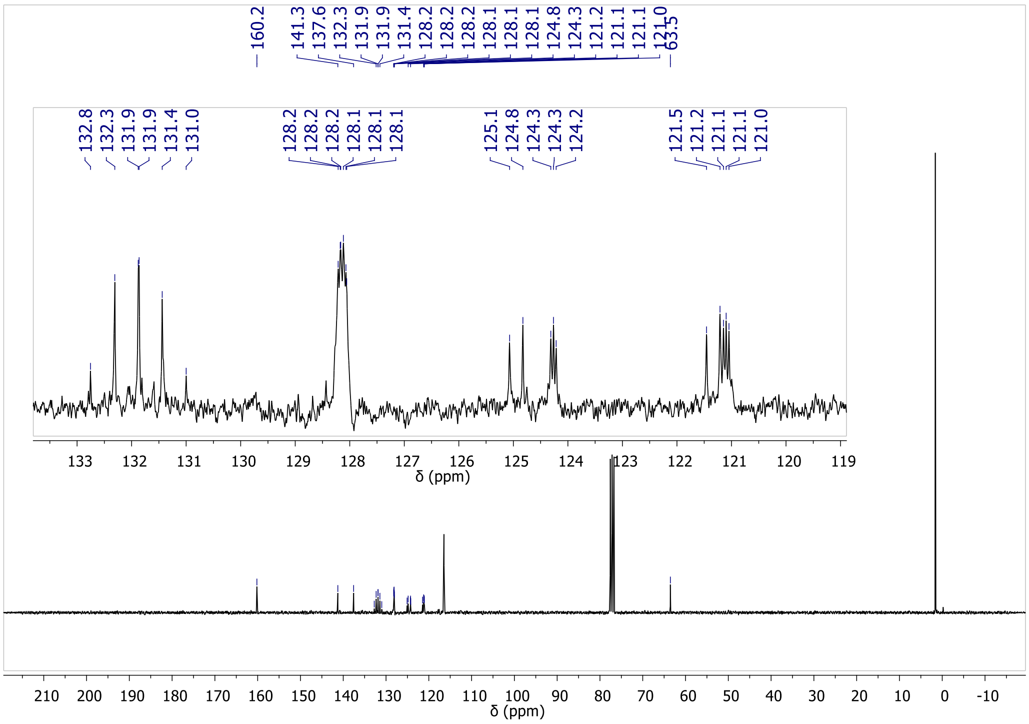

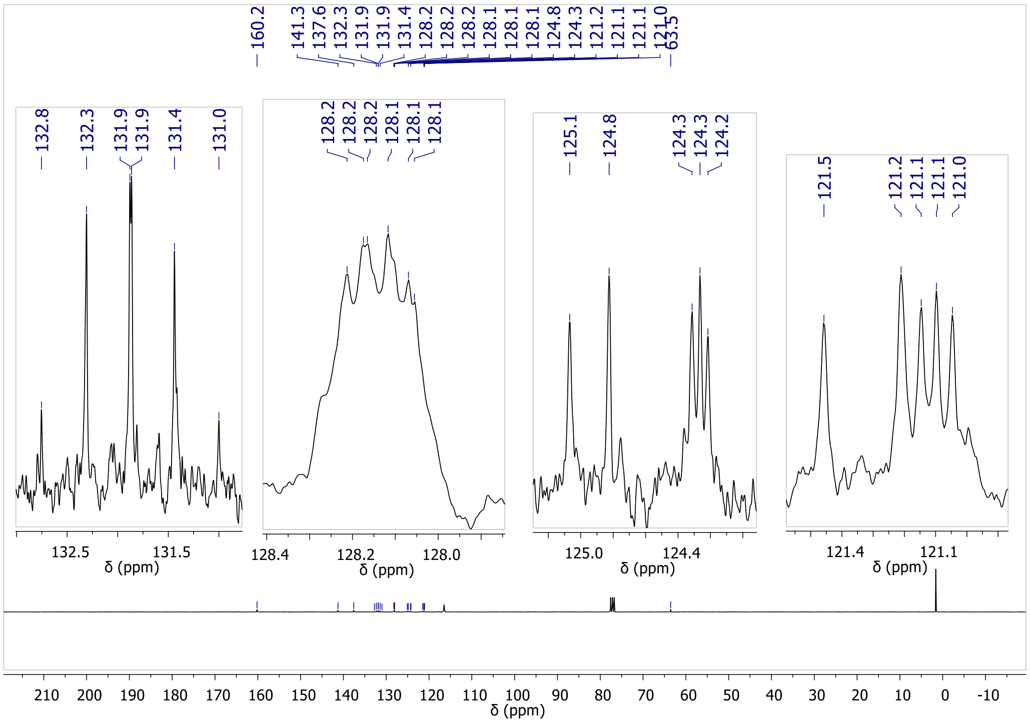

160.2
141.3
137.6
132.3
131.9
131.9
131.4
128.2
128.2
128.2
128.1
128.1
128.1
124.8
124.3
121.2
121.1
121.1
121.0
63.5
δ (ppm)

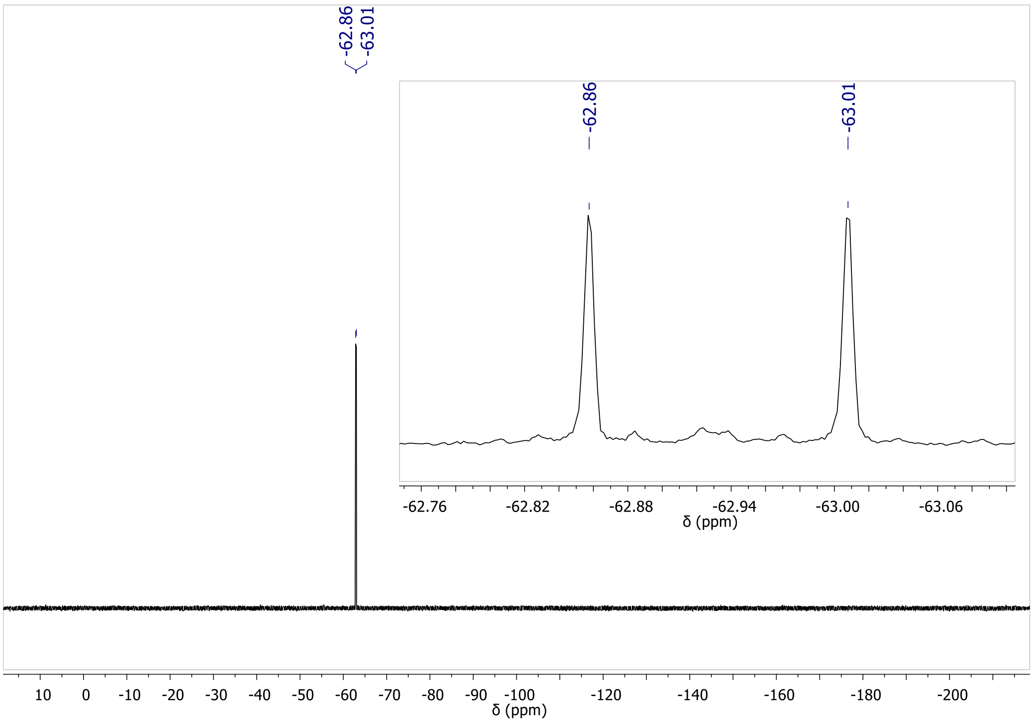

-62.86
-63.01
δ (ppm)

4-(((4-cyanobenzyl)imino)methyl)benzonitrile (11f) [3–6]

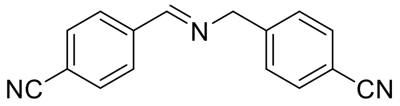

No purification was needed (the crude mixture was filtered off, and the solvent was evaporated); 77 % yield; white solid. ^{1}H-NMR (300 MHz, $CDCl_3$) δ 8.38 (s, 1H), 7.81 (d, *J* = 8.4 Hz, 2H), 7.64 (d, *J* = 8.3 Hz, 2H), 7.56 (d, *J* = 8.4 Hz, 2H), 7.39 (d, *J* = 8.5 Hz, 2H), 4.80 (s, 2H) ppm. ^{13}C-NMR (75 MHz, $CDCl_3$) δ 161.2, 144.6, 139.7, 135.9, 132.4, 128.9, 128.6, 128.5, 128.2, 123.7, 113.9, 63.9 ppm.

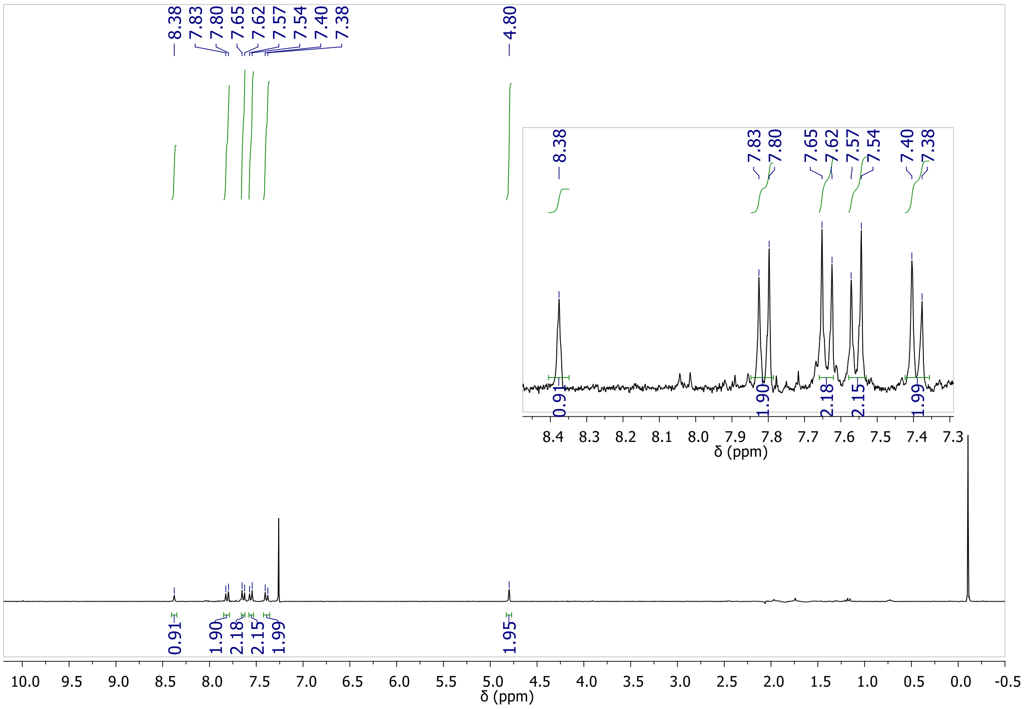

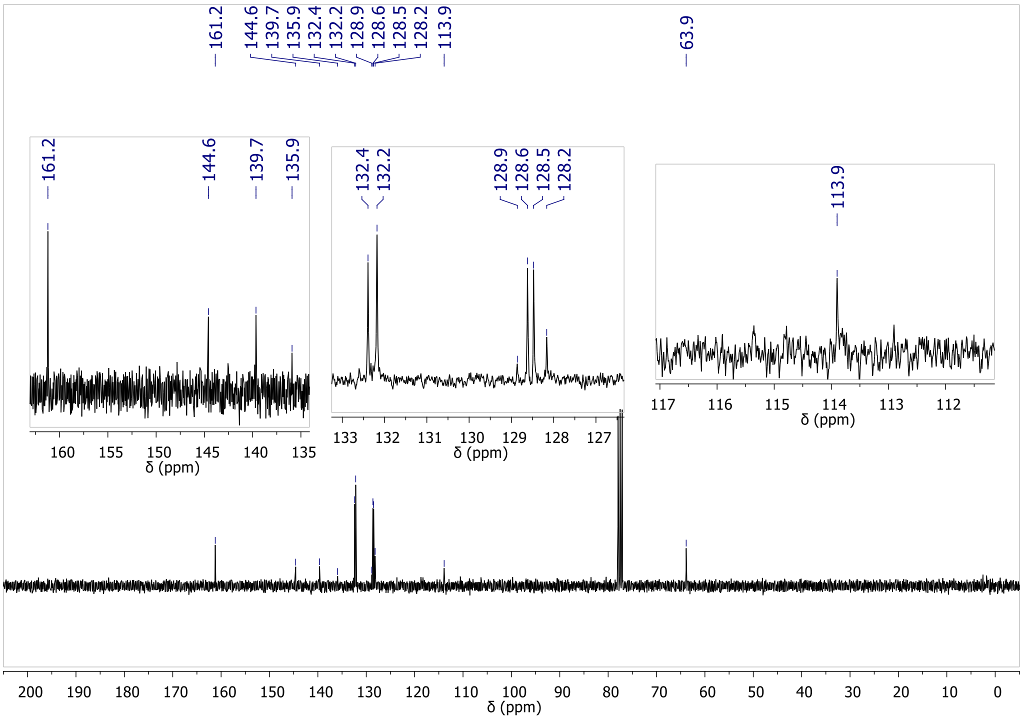

2-(((2-hydroxybenzyl)imino)methyl)phenol (11g) [3–6]

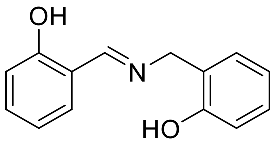

No purification was needed (the crude mixture was filtered off, and the solvent was evaporated); 91 % yield; beige solid. ^{1}H-NMR (300 MHz, *CDCl$_3$*) δ 8.42 (s, 1H), 7.34-7.16 (m, 4H), 6.96-6.80 (m, 4H), 4.83 (s, 2H) ppm. ^{13}C-NMR (75 MHz, $CDCl_3$) δ 162.2, 138.5, 134.6, 133.2, 132.8, 132.1, 130.0, 129.1, 128.7, 127.8, 127.7, 125.4, 123.8, 64.5 ppm.

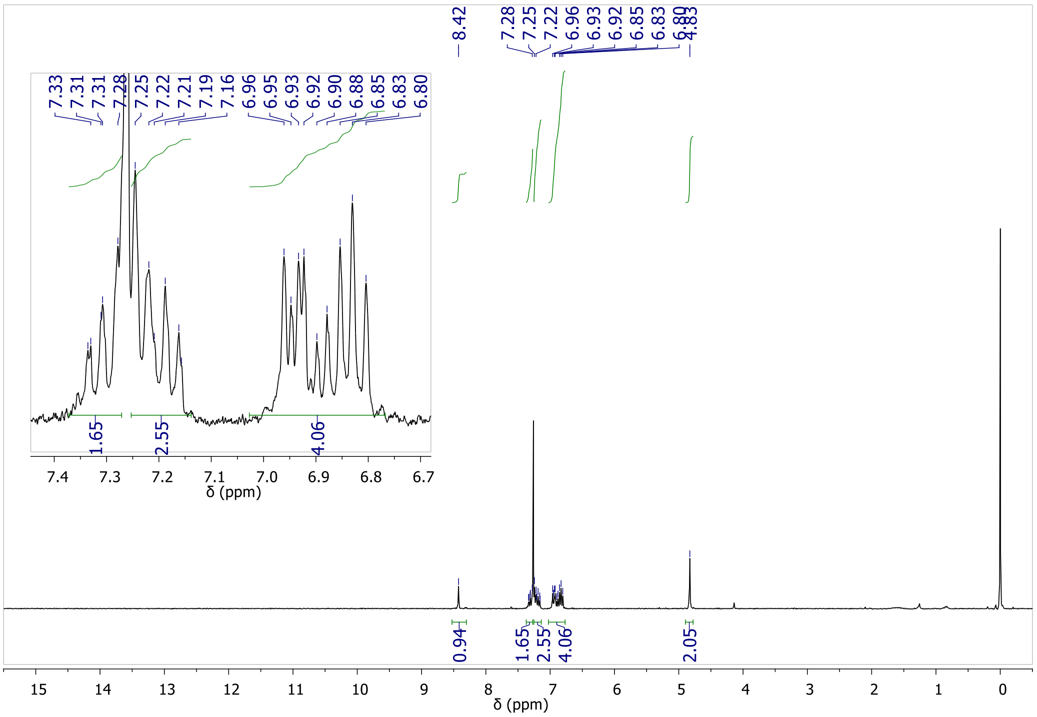

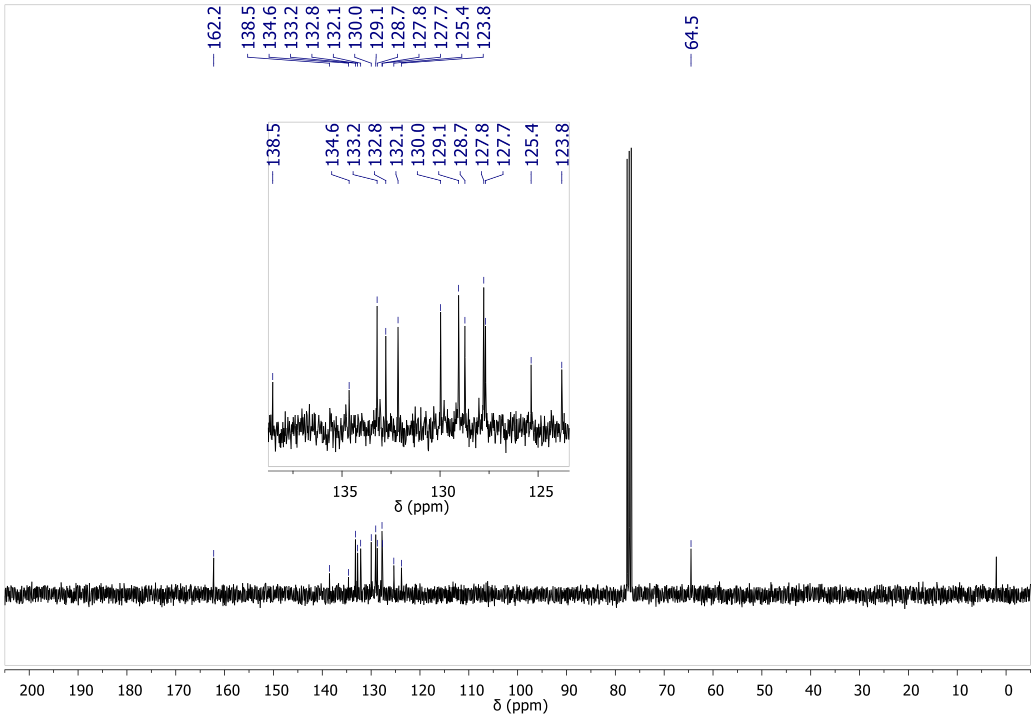

4-(((4-aminobenzyl)imino)methyl)aniline (11h) [3–6]

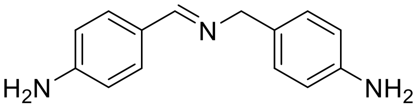

No purification was needed (the crude mixture was filtered off, and the solvent was evaporated); 30 % yield; yellow oil. ^{1}H-NMR (300 MHz, $CDCl_3$) δ 8.20 (s, 1H), 7.57 (d, *J* = 8.5 Hz, 2H), 7.10 (d, *J* = 8.5 Hz, 2H), 6.66 (dd, *J* = 8.4, 1.8 Hz, 4H), 4.65 (s, 2H) ppm. ^{13}C-NMR (75 MHz, $CDCl_3$) δ 172.3, 138.5, 130.5, 129.9, 129.1, 115.2, 114.6, 113.0, 110.2, 22.6 ppm.

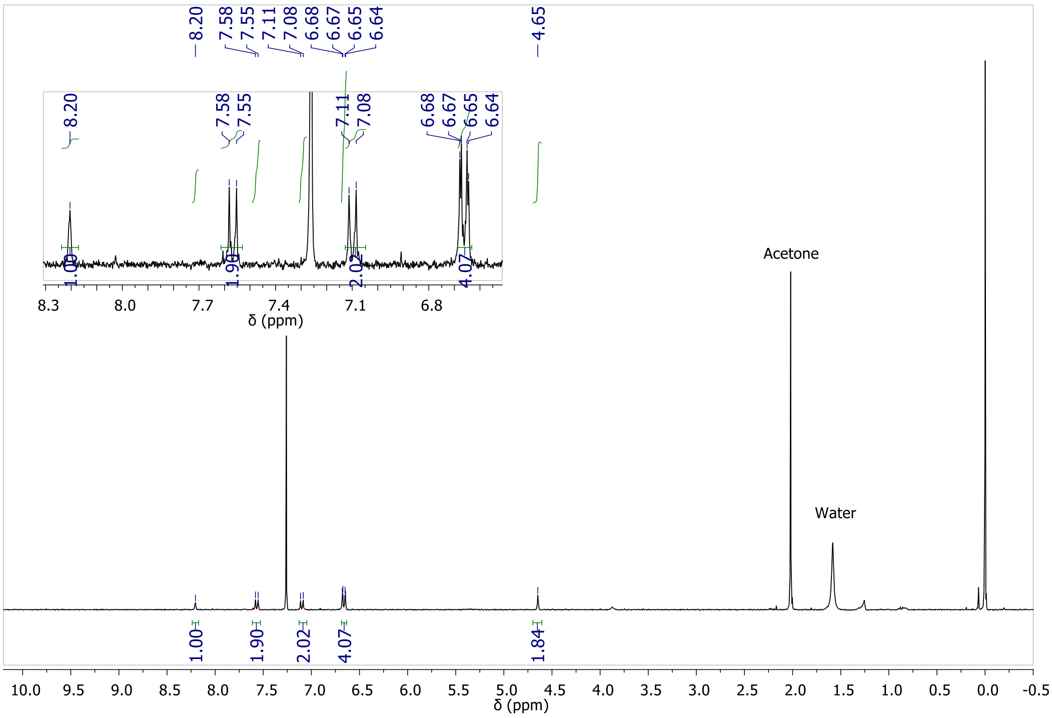

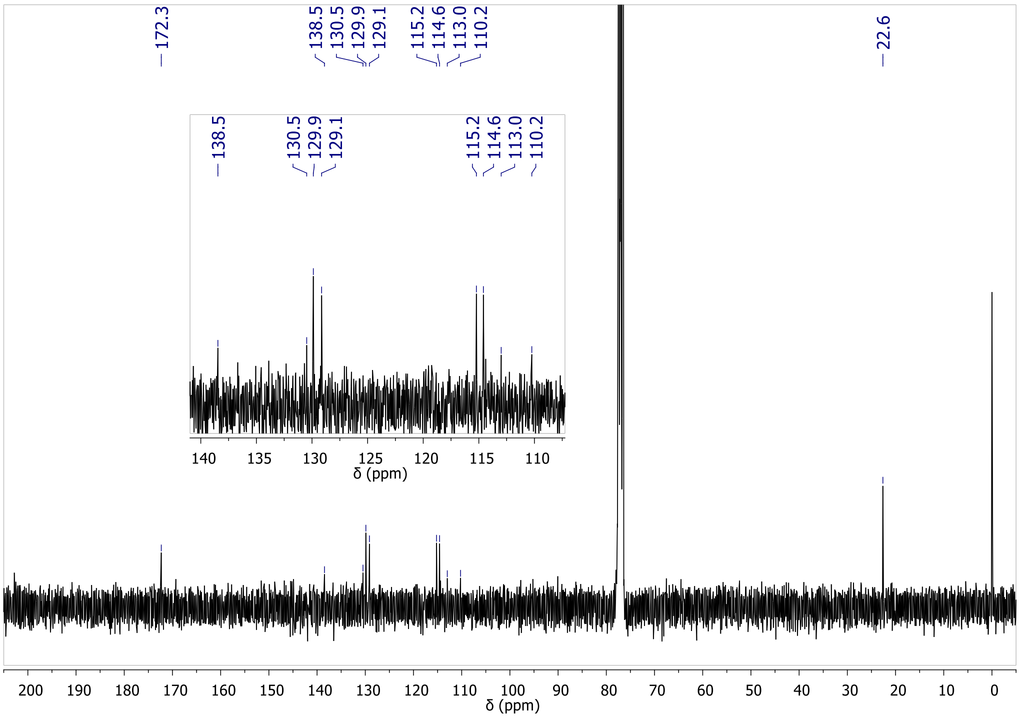

N-(4-bromobenzyl)-1-(4-bromophenyl)methanimine (11i) [3–6]

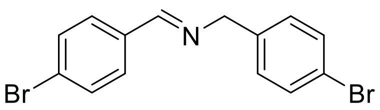

No purification was needed (the crude mixture was filtered off, and the solvent was evaporated); 99 % yield; white solid. ^{1}H-NMR (300 MHz, $CDCl_3$) δ 8.32 (s, 1H), 7.64 (dd, *J* = 8.5, 1.8 Hz, 2H), 7.55 (dd, *J* = 8.5, 1.9 Hz, 2H), 7.46 (dd, *J* = 8.5, 2.0 Hz, 2H), 7.20 (dd, *J* = 8.2, 1.5 Hz, 2H), 4.74 (s, 2H) ppm. ^{13}C-NMR (75 MHz, $CDCl_3$) δ 161.1, 138.1, 134.8, 131.9, 131.6, 129.7, 129.7, 125.4, 121.0, 64.2 ppm.

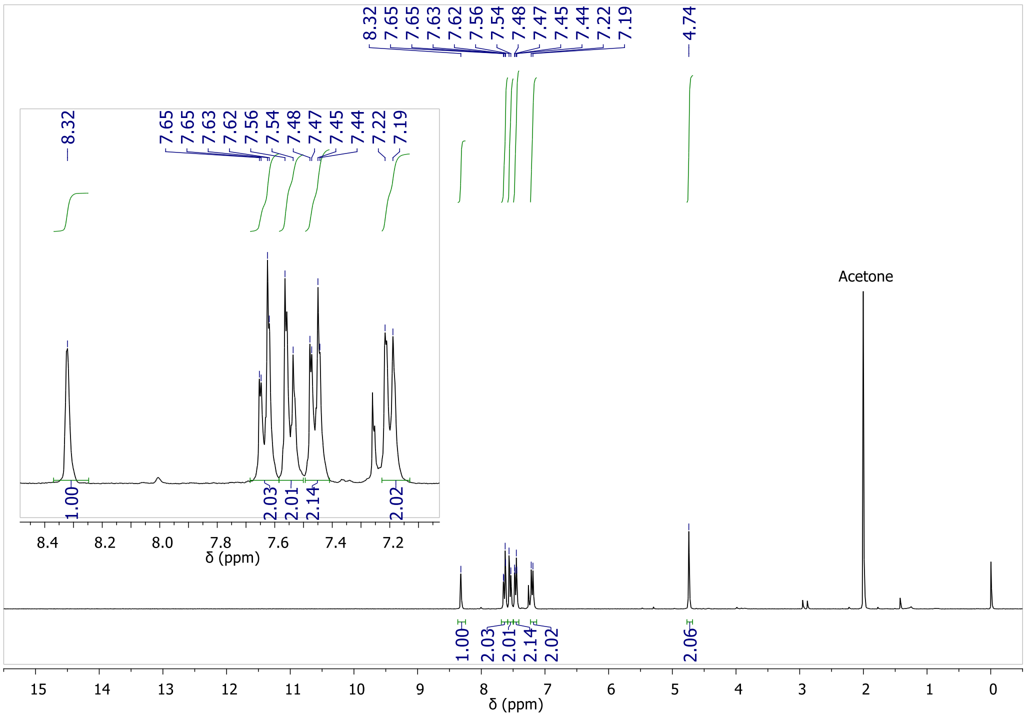

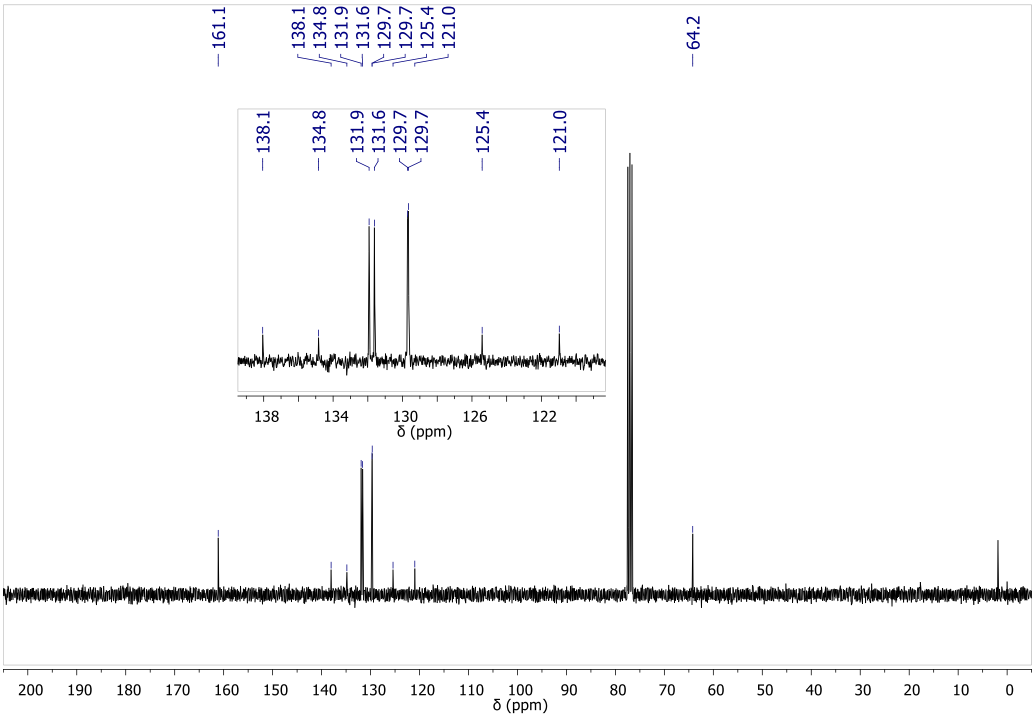

***N*-(4-chlorobenzyl)-1-(4-chlorophenyl)methanimine (11j)** [3–6]

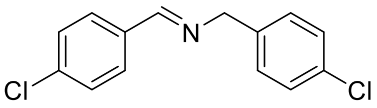

No purification was needed (the crude mixture was filtered off, and the solvent was evaporated); 96 % yield; white solid. ^{1}H-NMR (300 MHz, $CDCl_3$) δ 8.35 (s, 1H), 7.71 (d, *J* = 8.5 Hz, 2H), 7.40 (d, *J* = 8.5 Hz, 2H), 7.32 (d, *J* = 8.5 Hz, 2H), 7.26 (d, *J* = 8.5 Hz, 2H), 4.77 (s, 2H) ppm. ^{13}C-NMR (75 MHz, $CDCl_3$) δ 160.9, 137.6, 137.0, 134.4, 132.9, 129.5, 129.3, 129.0, 128.7, 64.2 ppm.

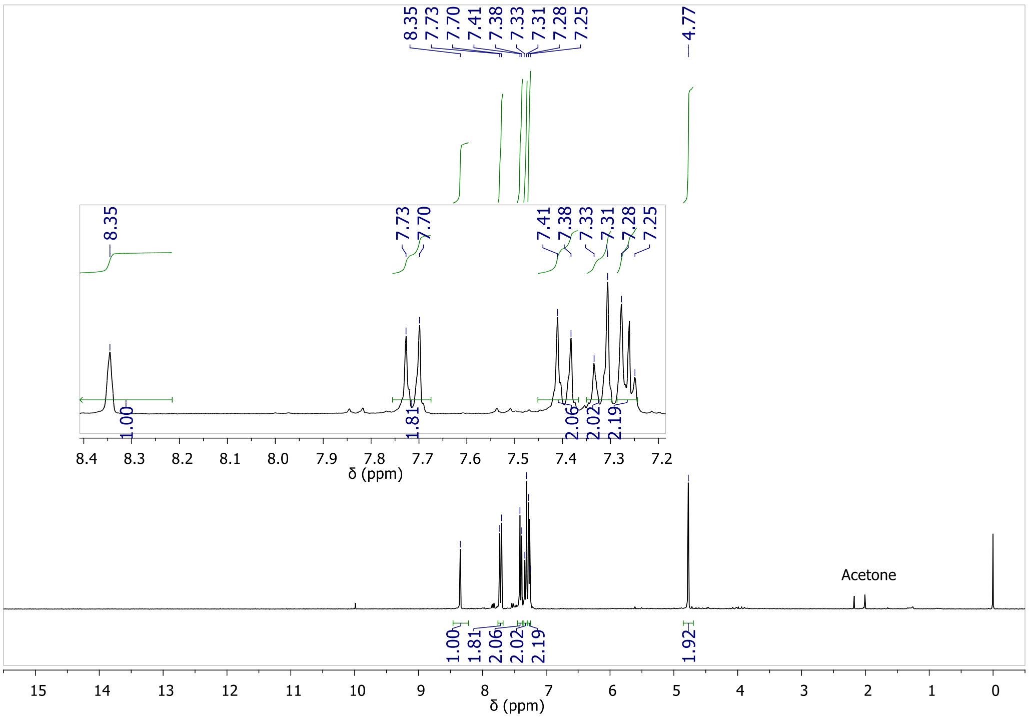

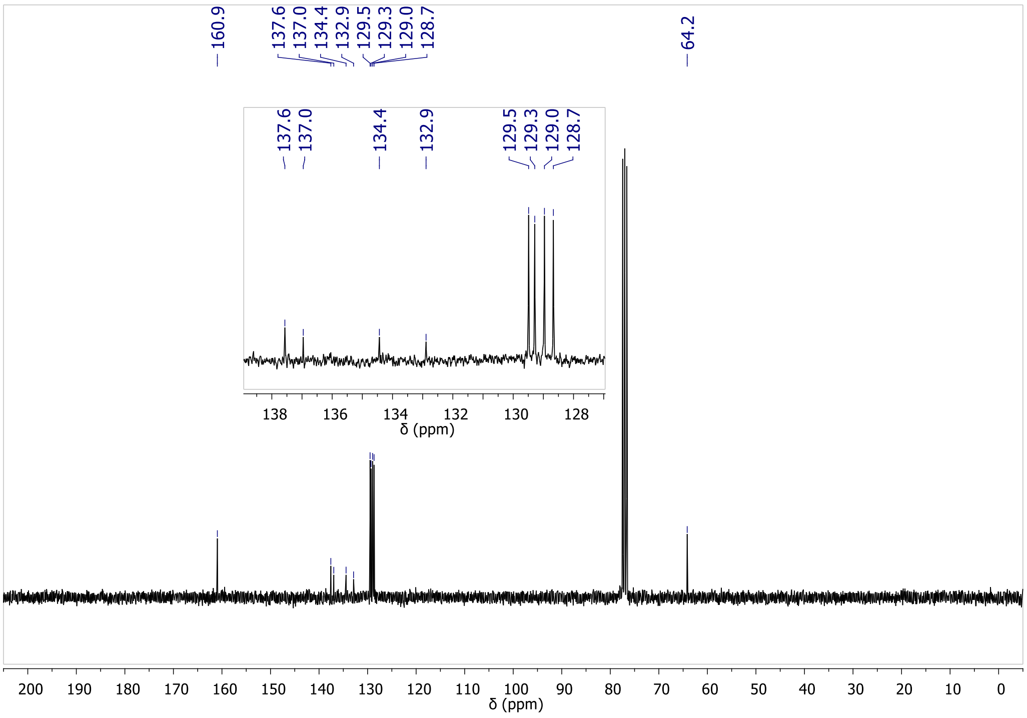

1-(thiophen-2-yl)-*N*-(thiophen-2-ylmethyl)methanimine (11k) [3–6]

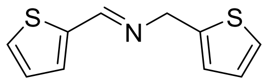

No purification was needed (the crude mixture was filtered off, and the solvent was evaporated); 87 % yield; beige solid. ^{1}H-NMR (300 MHz, $CDCl_3$) δ 8.42 (s, 1H), 7.42 (d, *J* = 5.0 Hz, 1H), 7.33 (d, *J* = 3.6 Hz, 1H), 7.24 (dd, *J* = 4.8, 1.5 Hz, 1H), 7.07 (dd, *J* = 5.0, 3.7 Hz, 1H), 7.01-6.98 (m, 2H), 4.95 (s, 2H) ppm. ^{13}C-NMR (75 MHz, $CDCl_3$) δ 155.5, 142.1, 141.5, 131.0, 129.4, 127.4, 126.9, 125.4, 124.9, 58.5.

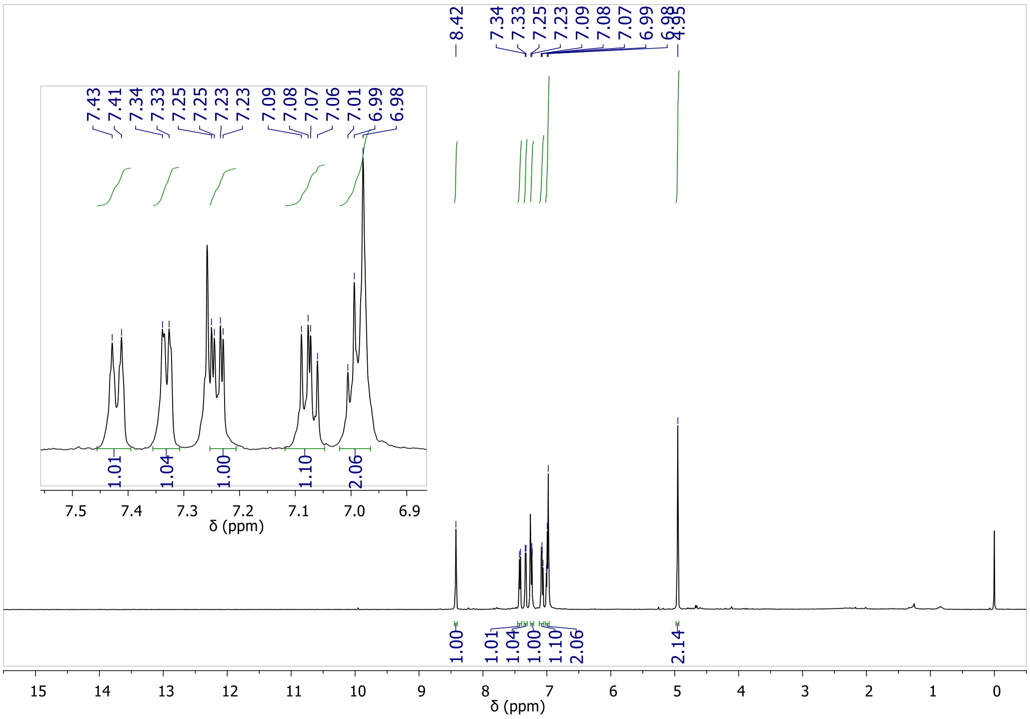

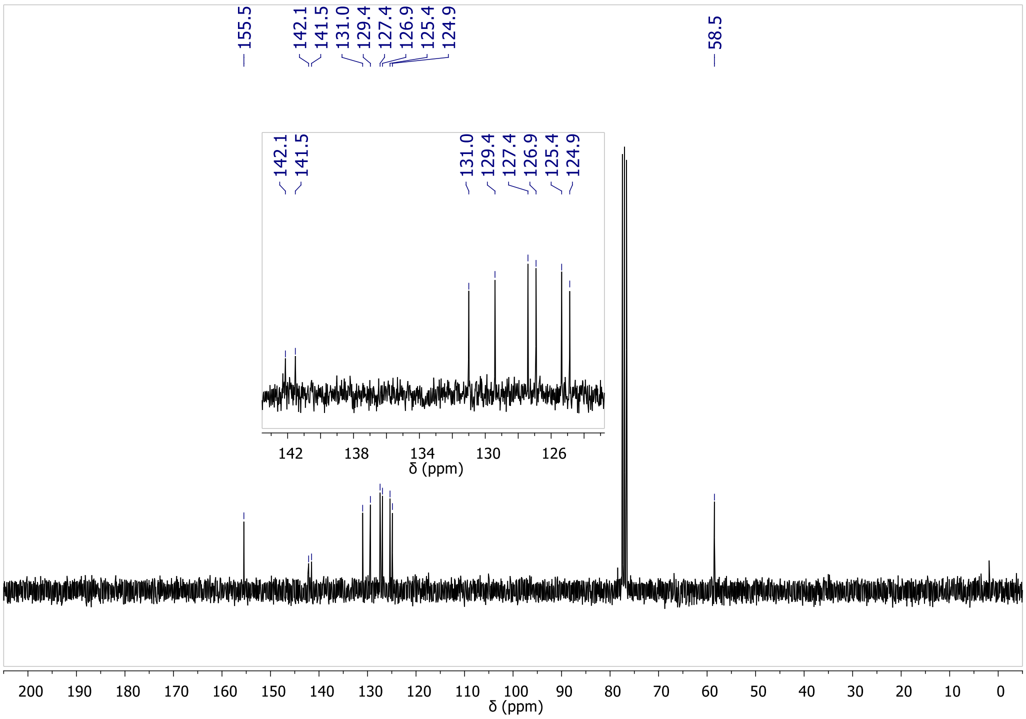

5. Mechanistic experiments

An oven-dried 10 mL vial equipped with a magnetic stir bar was charged with 2 mg of CTF **9**, 1,3,5-trimethoxybenzene (16.8 mg, 0.1 mmol) as standard for quantitative NMR and 2 mL of acetonitrile. Then, the amine **10a** (21.8 µL, 0.2 mmol) and 0.05 equiv. of the corresponding additive (0.1 mmol) were added. The vial was closed with a PTFE / rubber septum and oxygen was bubbled in the reaction mixture for 5 minutes. The reaction mixture with an O_2 balloon was stirred under blue-LED irradiation at 25 ˚C for 3 h.

Table S3. Mechanistic experiments in the presence of enhancers or scavengers and CTF **9**.

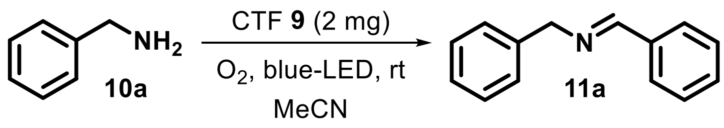

Entry	Additive (equiv.)	Time (h)[a]	Yield (%)[a]
1	-	3	61
2	DABCO (0.5)	3	33
3	Benzoquinone (0.5)	3	58
4	iPrOH (0.5)	3	49
5	$Na_2C_2O_4$ (0.5)	3	60
6	$AgNO_3$ (0.5)	3	25

[a] Determined by ^{1}H-NMR with 1,3,5-trimethoxybenzene as quantitative standard.

For the detection of hydrogen peroxide, titanium (IV) oxalate method was employed.[7,8] After a 14 h catalytic run, the reaction was filtered, MeCN was evaporated, and the crude was extracted with 1 mL of DCM and 1 mL of distilled water. The organic phase was separated, and 0.5 equivalents of $Ti(C_2O_4)_2$ (22.4 mg, 0.1 mmol) were added into the aqueous phase. Absorbance at λ = 407 nm confirms the existence of H_2O_2.

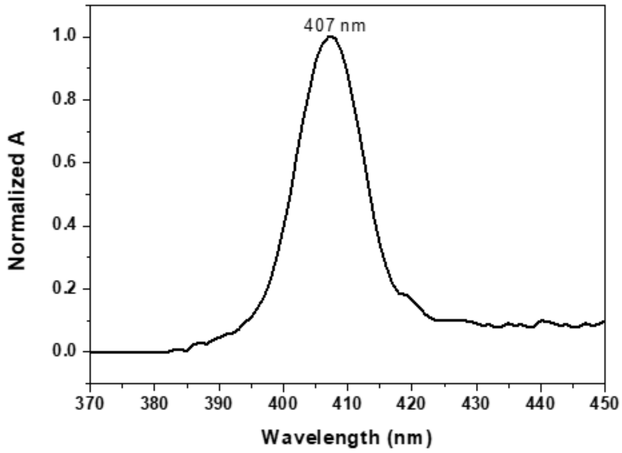

Figure S1: Detection of H_2O_2 by titanium oxalate method.

An additional experiment was performed to corroborate the formation of singlet oxygen under blue LED irradiation using 9,10-diphenylanthracene as known scavenger.[9] A 10 mL screw vial equipped with a magnetic stir bar was charged with 2 mg of CTF **9** and 9,10-diphenylanthracene ($2 \cdot 10^{-1}$ mM) in toluene (2 mL). Then, the mixture was sonicated to disperse the CTF. The closed vial was placed under blue LED irradiation and the reaction mixture was stirred at room

temperature. The reaction was followed by UV-Vis spectrophotometer for 21 h (**CTF**). Two blanks were carried out at once. One without CTF was carried out under blue LED irradiation (**blank**), and the other one with CTF was carried out in the dark (**dark**). After 21 h of irradiation, the final concentration of 9,10-diphenylanthracene in **dark** was exactly the same, $2 \cdot 10^{-1}$ mM, in **blank** was slightly lower, $1.8 \cdot 10^{-1}$ mM, and in **CTF** was practically priceless, $2.5 \cdot 10^{-2}$ mM. This shows, again, that our CTF is capable of producing singlet oxygen.

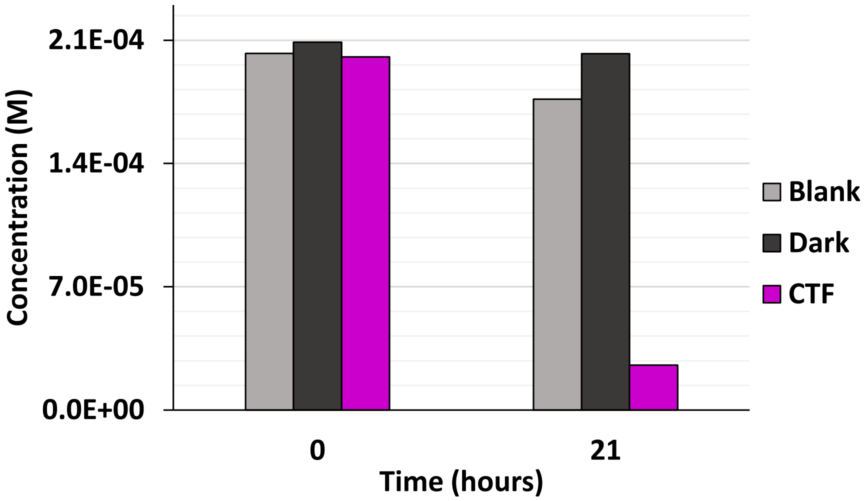

Figure S2. Concentration of 9,10-diphenylantracene after being irradiated with blue LED irradiation for 21 h in the case of **blank** and **CTF** and after being in the dark for 21 h in the case of **dark**.

6. Leaching test

An oven-dried 10 mL vial equipped with a magnetic stir bar was charged with 2 mg of the photocatalyst material (imine-based material **7**, hydrazone-based material **8** or CTF **9**), 1,3,5-trimethoxybenzene (16.8 mg, 0.1 mmol) as standard for quantitative NMR and 2 mL of acetonitrile as solvent. Then, the amine **10a** (21.8 µL, 0.2 mmol) was added. The vial was closed with a PTFE / rubber septum and oxygen was bubbled in the reaction mixture for 5 minutes. The reaction mixture was stirred under blue-LED irradiation with an O_2 balloon at 25 ˚C for 14 h with full conversion to the imine product **11a**. After that, the vial was opened and the crude mixture was filtered through membrane filter to retire the photocatalyst material. More amine **10a** (21.8 µL, 0.2 mmol) was added again for a next run without the photocatalyst. The vial was closed and the reaction mixture was bubbled again with oxygen following the same procedure than before. After 14 h, the yield was determined by ^{1}H-NMR from the aliquot taking at time zero and at end time.

Table S4. Leaching text with imine-based material **7**, hydrazone-based material **8** and CTF **9.**

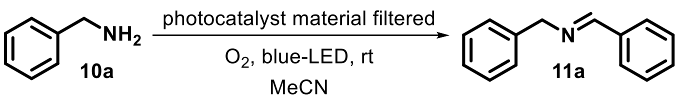

Entry	Photocatalyst material filtered[a]	Time (h)[a]	Yield (%)
1	Imine-based material **7**	16	61
2	Hydrazone-based material **8**	16	32
3	CTF **9**	16	10

[a] Time of a new run. After 14 h under blue-LED irradiation the crude mixture was filtered to retire the photocatalyst material and more amine **10a** was added to carry out a new run.

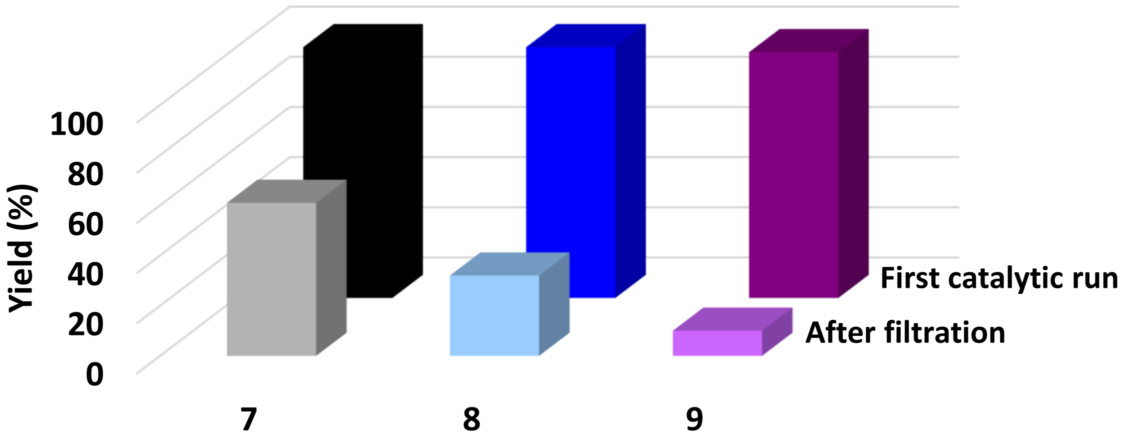

Figure S3. First catalytic run, yield of product after 14 h before filtering the photocatalytic material **7**, **8** or **9**. After filtration, yield of product after 14 h after filtering the photocatalytic material **7**, **8** or **9**.

An oven-dried 10 mL vial equipped with a magnetic stir bar was charged with 2 mg of CTF **9**, 1,3,5-trimethoxybenzene (16.8 mg, 0.1 mmol) as standard for quantitative NMR and 2 mL of acetonitrile as solvent. Then, the amine **10b**, **10c**, **10d**, **10j** or **10k** (0.2 mmol) was added. The vial was closed with a PTFE / rubber septum and oxygen was bubbled in the reaction mixture for 5 minutes. The reaction mixture was stirred under blue-LED irradiation with an O_2 balloon at 25 ˚C for 14 h with almost full conversion to the imine product **11b**, **11c**, **11d**, **11j** or **11k**,

respectively. After that, the vial was opened and the crude mixture was filtered through membrane filter to retire the photocatalyst material CTF **9**. More amine **10b**, **10c**, **10d**, **10j** or **10k** (0.2 mmol) was added again for a next run without the photocatalyst. The vial was closed and the reaction mixture was bubbled again with oxygen following the same procedure than before. After 14 h, the yield was determined by ^{1}H-NMR from the aliquot taking at time zero and at end time.

Table S5. Leaching text with CTF **9**.

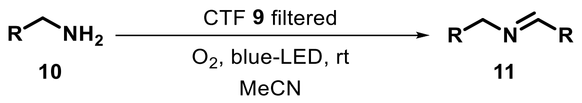

Entry	R	Imine	Time (h)[a]	Yield (%)
1	*p*-OCH_3-C_6H_4	**11b**	14	13
2	*p*-CH_3-C_6H_4	**11c**	14	8
3	*p*-CF_3-C_6H_4	**11d**	14	12
4	*p*-Cl-C_6H_4	**11j**	14	4
5	2-Thienyl	**11k**	14	8

[a] Time of a new run. After 14 h under blue-LED irradiation the crude mixture was filtered to retire the CTF **9** and more amine **10** was added to carry out a new run.

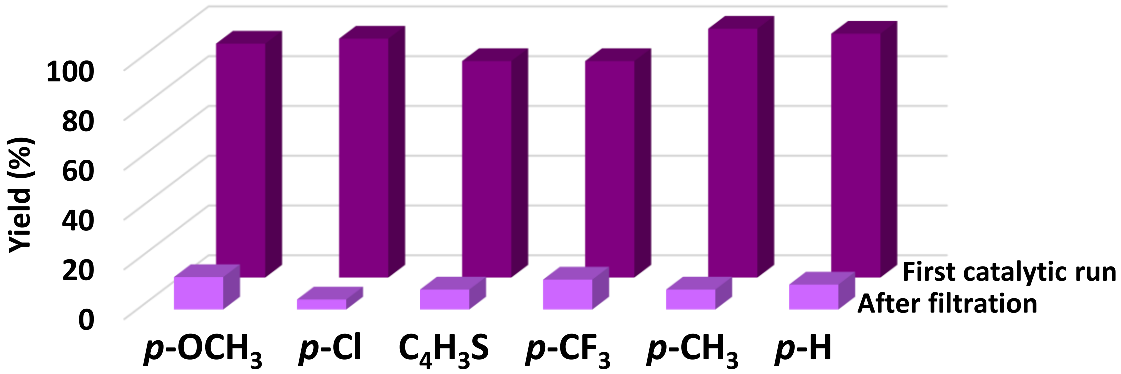

Figure S4. First catalytic run, yield of product after 14 h before filtering the photocatalytic material **9** with each substrate. After filtration, yield of product after 14 h after filtering the photocatalytic material **9** with each substrate.

7. Recyclability test

An oven-dried 10 mL vial equipped with a magnetic stir bar was charged with 3 mg of the photocatalyst material (imine-based material **7**, hydrazone-based material **8** or CTF **9**), amine **10a** (21.8 µL, 0.2 mmol) and 2 mL of acetonitrile as solvent. The vial was closed with a PTFE / rubber septum and oxygen was bubbled in the reaction mixture for 5 minutes. The reaction mixture was stirred under blue-LED irradiation for 14 h with an O_2 balloon at 25 ˚C. The final conversion was determined by ^{1}H-NMR. After each run, the crude mixture was centrifuged recovering the photocatalyst material to carry out a new photocatalytic cycle of 4 h. Seven consecutive cycles were carried out following the same procedure.

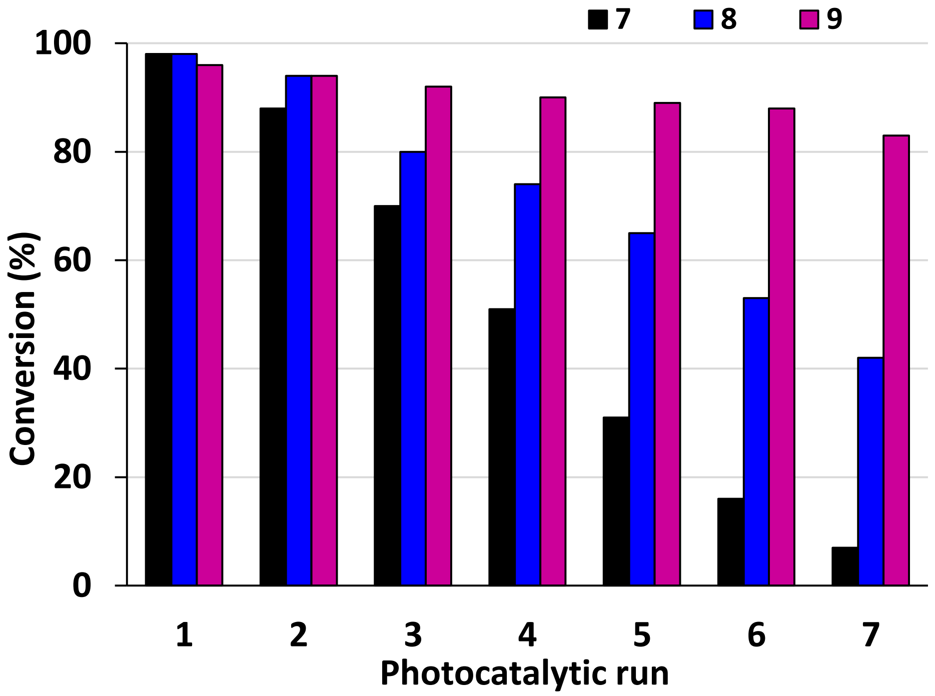

Figure S5. Photocatalytic runs with imine-based material **7**, hydrazone-based material **8** and CTF **9** in the oxidative coupling of amine **10a.**

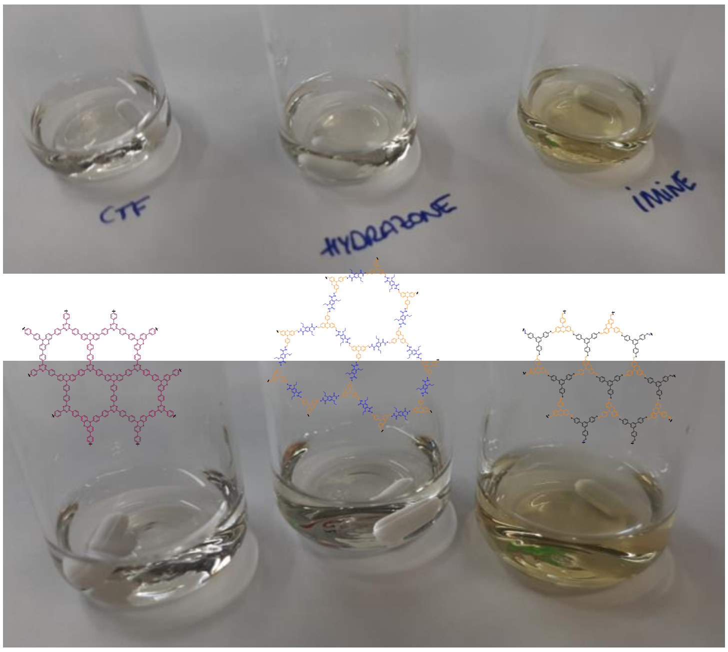

Figure S6. Reaction crude after filtering the photocatalytic materials.

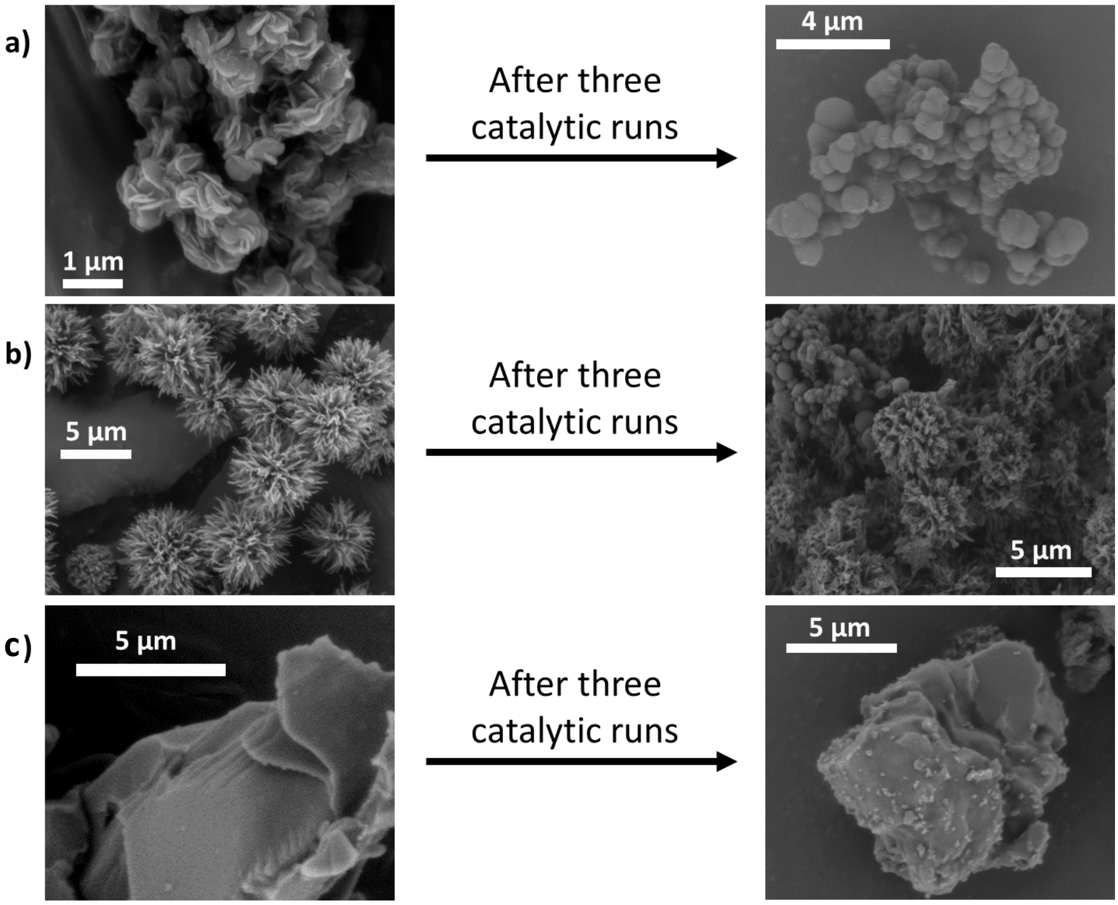

Figure S7. SEM images of imine-based material **7** (a), hydrazone-based material **8** (b) and CTF **9** (c) before the photocatalytic run (left) and after three photocatalytic runs (right).

8. Calculation of chemical erosion

Based in a published project for our research group,[10] some experiments with methoxyaniline or (4-methoxyphenyl)methanamine were carried out in order to check if these reagents react with the material and to recognize possible byproducts.

An oven-dried 10 mL vial equipped with a magnetic stir bar was charged with 5 mg of the photocatalyst material (imine-based material **7**, hydrazone-based material **8** or CTF **9**) and methoxyaniline **12** (12.3 mg, 0.1 mmol) or (4-methoxyphenyl)methanamine **10b** (13 µL, 0.1 mmol) without solvent. The vial was closed with a PTFE / rubber septum and the reaction was stirred for 1 h at 60 ˚C. Then, $CDCl_3$ was directly added to the reaction mixture and the solution was analyzed by ^{1}H-NMR.

To calculate the amount of deteriorated material is necessary to consider the amount of imine functional groups per 5 mg of material:

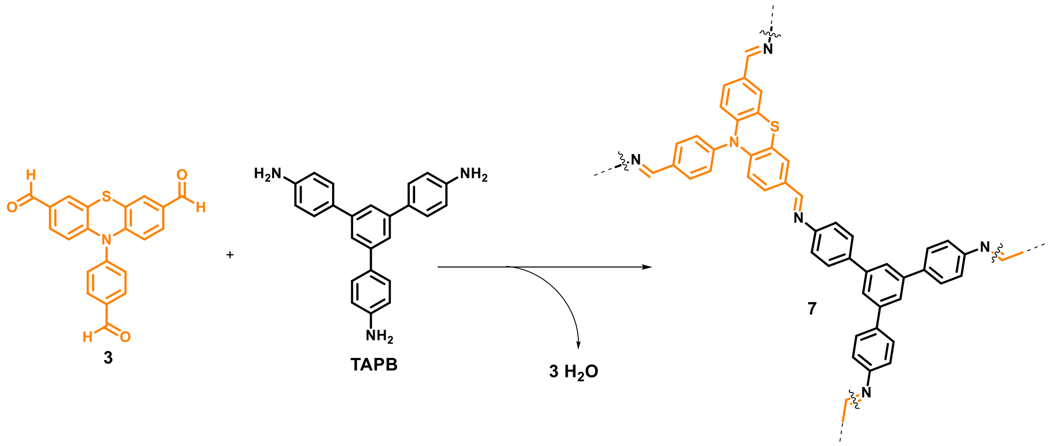

5 mg / 656.79 g mol^{-1} = 0.0076 mmol

There are three imine functional groups in this subunit:

0.0076 mmol · 3 = 0.0228 mmol of imine functional groups in 5 mg of material.

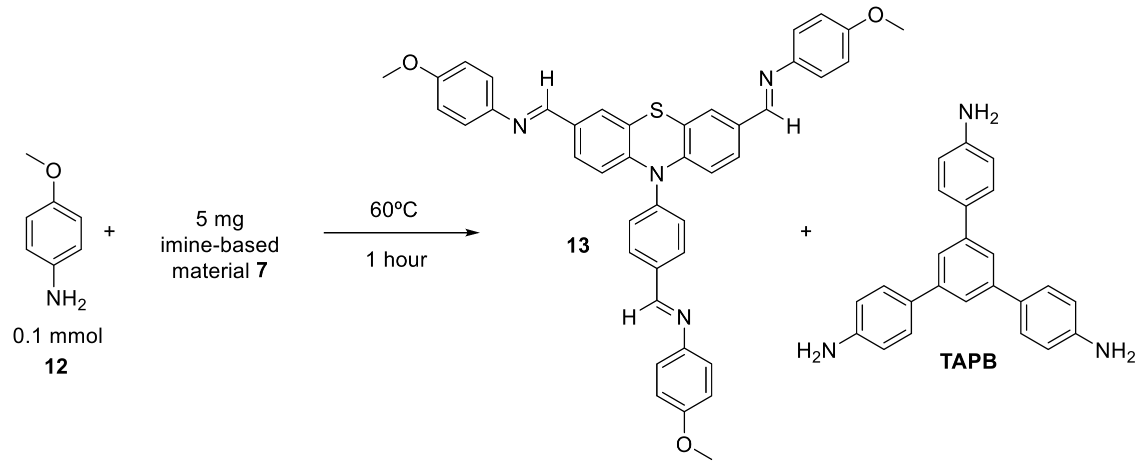

Only material **7** was chemically erosioned after 1 h under these conditions.

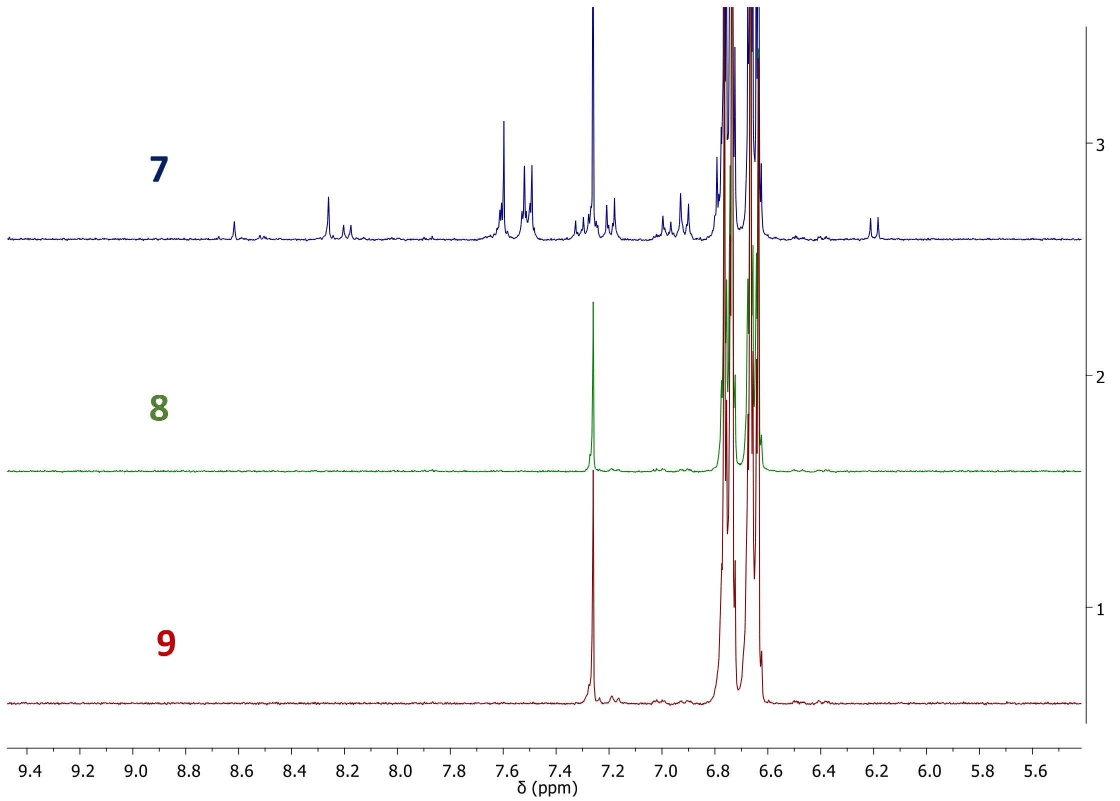

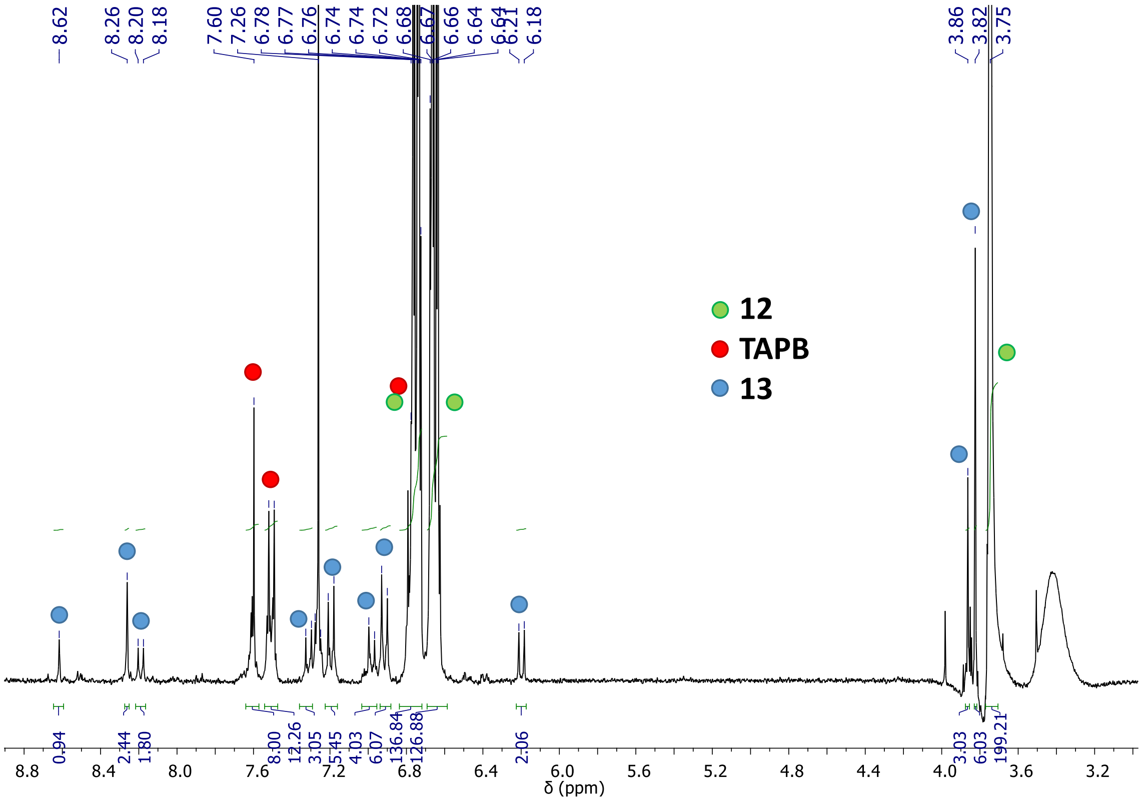

To calculate the ratio (byproduct **13** : methoxyaniline **12**) we use the signal of 3.86 ppm for byproduct **13** and the signal at 6.65 ppm for methoxyaniline **12**. The 3.86 ppm signal integrates for three protons, and the 6.65 ppm signal integrates for two protons, so the molar ratio is (1.01 : 63.44).

Considering the stoichiometry, three molecules of methoxyaniline **12** are necessary for making one molecule of byproduct **13**, so the ratio of (reacted methoxyaniline: methoxyaniline left) is (3.03 : 63.44). In other words, 4.6 % of the initial methoxyaniline has reacted.

In the reaction, there was 0.1 mmol of initial methoxyaniline **12**. Applying this percentage, 0.0046 mmol of the methoxyaniline has reacted with 0.0046 mmol of the imines functional groups of the material. Therefore, the percentage of deteriorated material is:

Chemical erosion material = (0.0046 mmol reacted imines / 0.0228 mmol total imines) · 100 = **20 %**

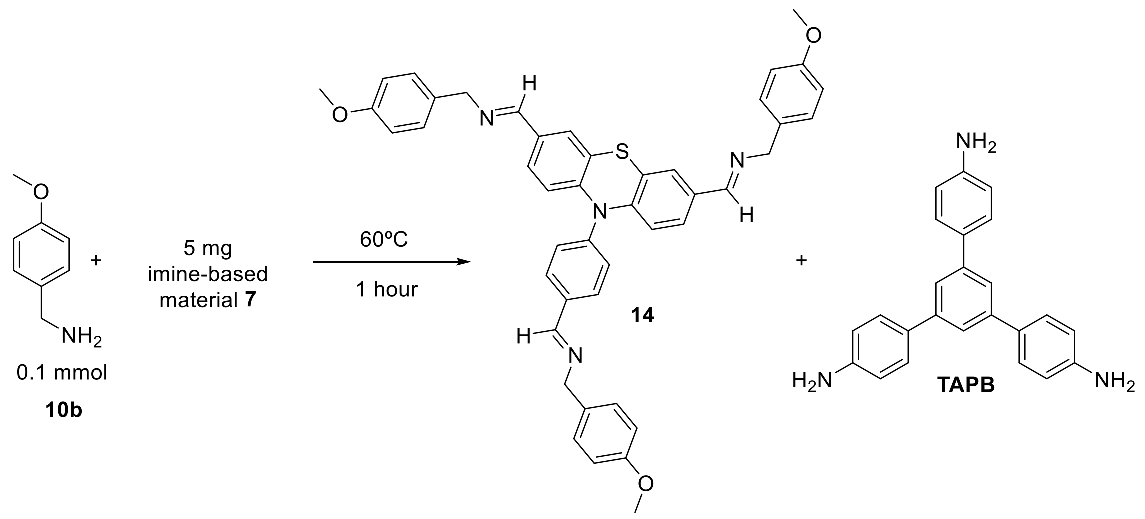

Only material **7** was chemically erosioned after 1 h under these conditions.

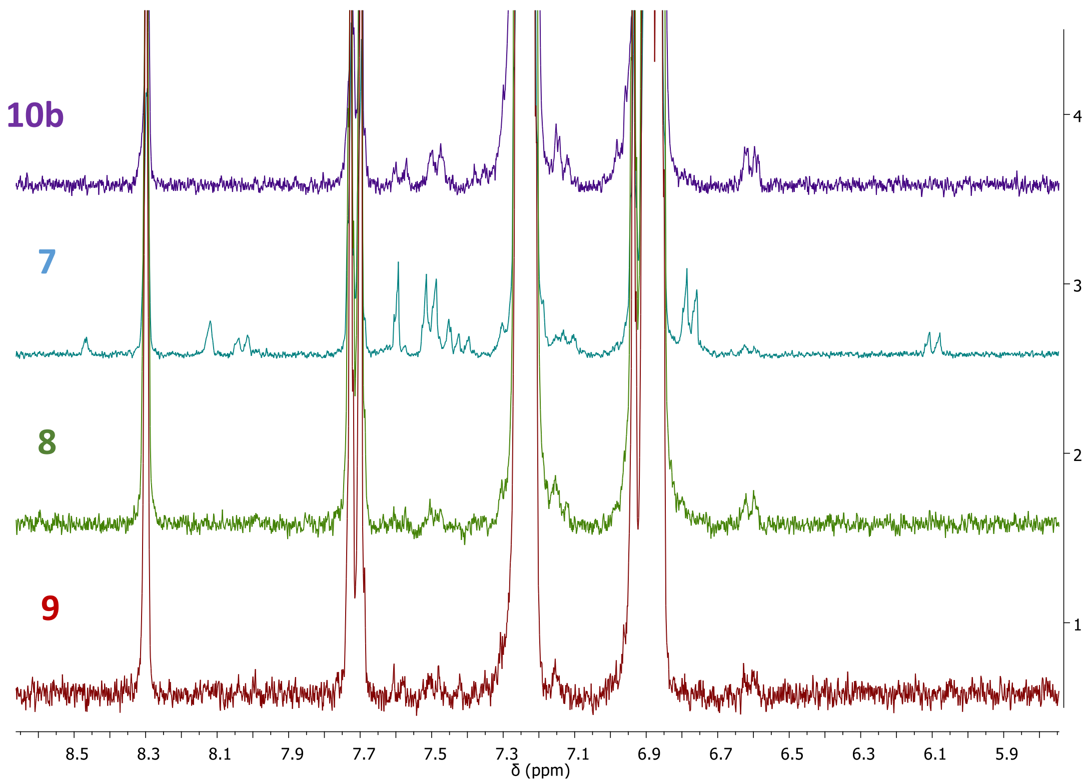

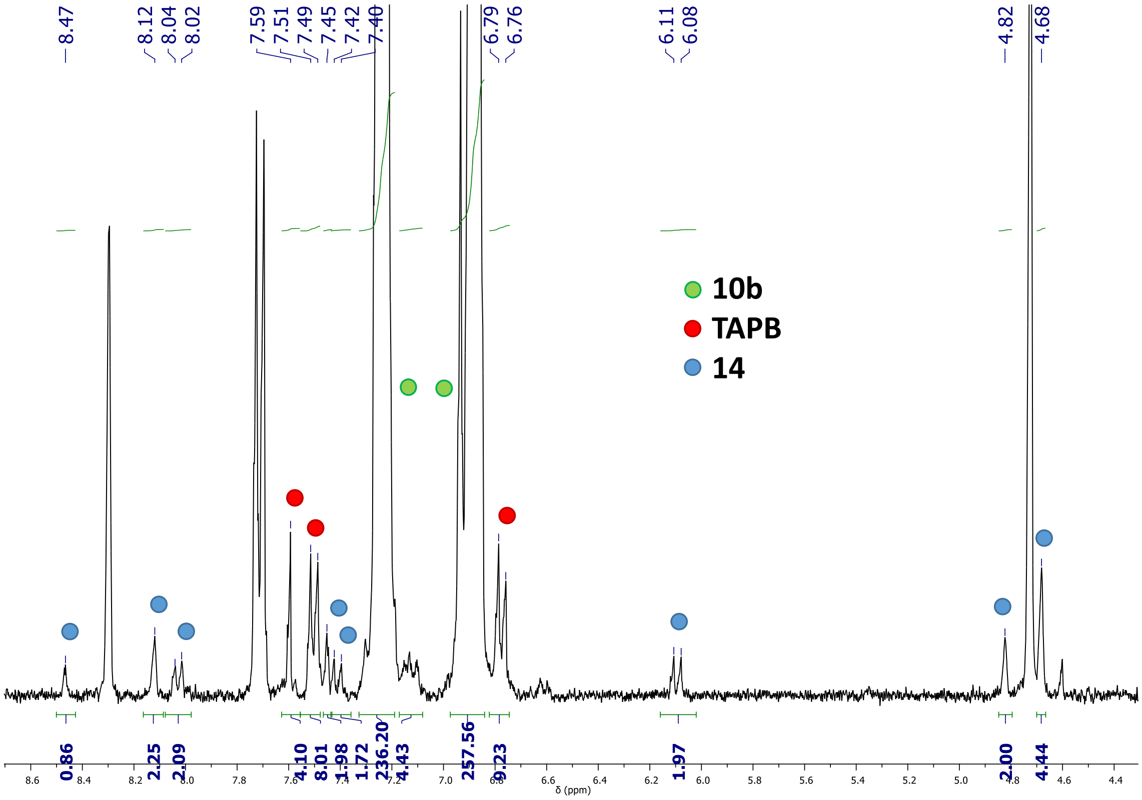

To calculate the ratio (byproduct **14** : (4-methoxyphenyl)methanamine **10b**) we use the signal of 4.82 ppm for byproduct **14** and the signal at 7.24 ppm for (4-methoxyphenyl)methanamine **10b**. The 4.82 ppm signal integrates for two protons, and the 7.24 ppm signal integrates for two protons, so the molar ratio is (1.00 : 118.10).

Considering the stoichiometry, three molecules of (4-methoxyphenyl)methanamine **10b** are necessary for making one molecule of byproduct **14**, so the ratio of (reacted (4-methoxyphenyl)methanamine: (4-methoxyphenyl)methanamine left) is (1.00 : 39.37). In other words, 2.5 % of the initial (4-methoxyphenyl)methanamine has reacted.

In the reaction, there was 0.1 mmol of initial (4-methoxyphenyl)methanamine **10b**. Applying this percentage, 0.0025 mmol of the (4-methoxyphenyl)methanamine has reacted with 0.0025 mmol of the imines functional groups of the material. Therefore, the percentage of deteriorated material is:

Chemical erosion material = (0.0025 mmol reacted imines / 0.0228 mmol total imines) · 100 = **11 %**

Additionally, we quantified the S content on the supernatant after 24 h of digestion with amine 10b at 60 ^{o} C. The results are presented in Table S6.

Table S6. Quantification of chemical erosion by ICP-OES after digestion treatment with amine **10b**.

Entry	Material	% S in the material[a]	10b	Total weight of reaction	Calculated ppm of S before the reaction	Measured ppm of S in supernatant[b]	% Erosion[c]
1	**7** (5 mg)	4.67	18.7 mg	23.7 mg	9848	2895	30
2	**8** (5 mg)	3.91	18.7 mg	23.7 mg	8245	815	10
3	**9** (5 mg)	5.13	18.7 mg	23.7 mg	10818	106	1

[a] Determined by elemental analysis. [b] Determined by ICP-OES after the reaction. [c] The % of erosion was calculated by dividing ppm of S in supernatant by ppm of S before the reaction.

9. FT-IR Spectroscopy: Attenuated Total Reflectance (ATR)

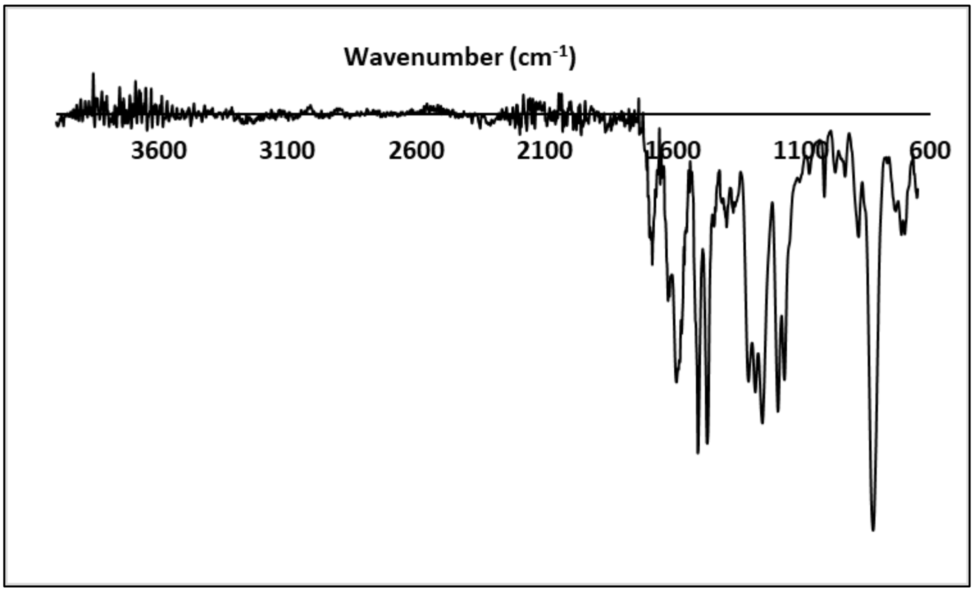

Peak (cm^{-1})	Assignment
3006 (vw)	Aromatic C-H stretching
1682	Imine C=N stretching
1587, 1505, 1466	Aromatic ring stretching

Figure S8. FT-IR spectra of material **7.**

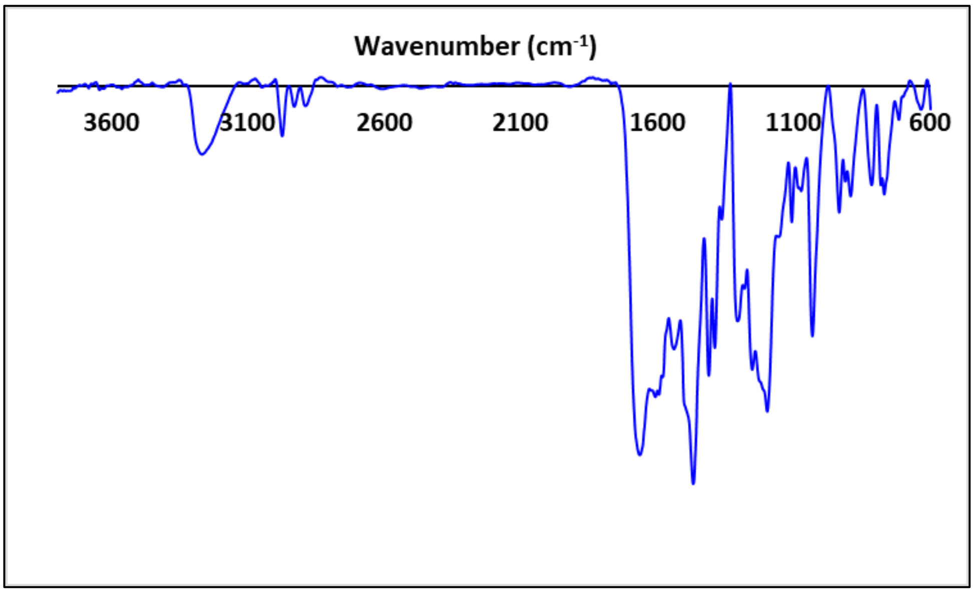

Peak (cm^{-1})	Assignment
3264	Amine N-H stretching
2976, 2892, 2888	Aliphatic C-H stretching (CH_2, CH_3)
3024 (vw)	Aromatic C-H stretching
1664	Imine C=N stretching
1595	Carbonyl C=O stretching
1595, 1539, 1469	Aromatic ring stretching
1469, 1412, 1390	Aliphatic C-H stretching (CH_2, CH_3)

Figure S9. FT-IR spectra of material **8.**

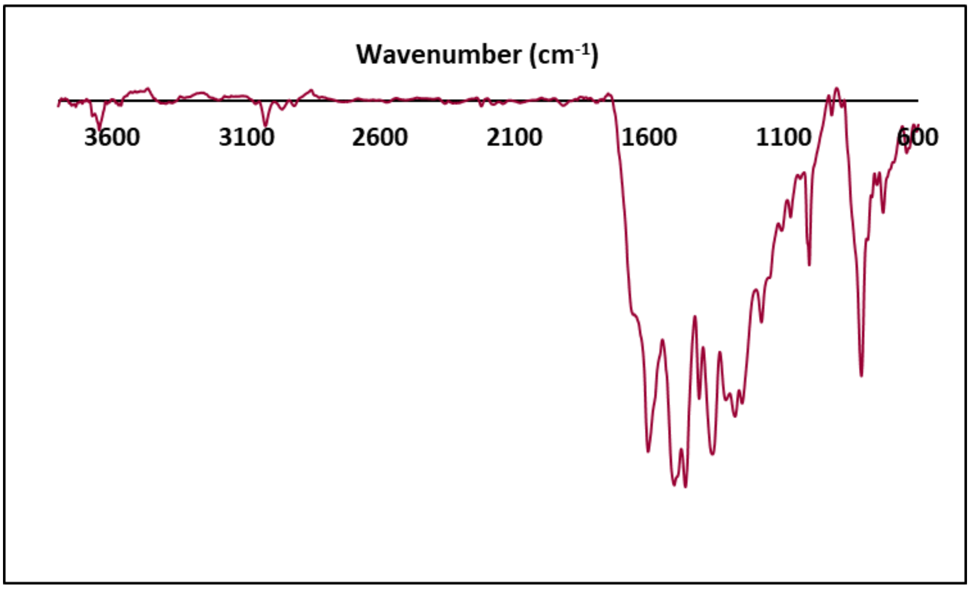

Peak (cm^{-1})	Assignment
3028, 3006	Aromatic C-H stretching
1605	Triazine C=N stretching
1508, 1467	Aromatic ring stretching

Figure S10. FT-IR spectra of CTF **9.**

10. Diffuse Reflectance Spectroscopy and Fluorescence

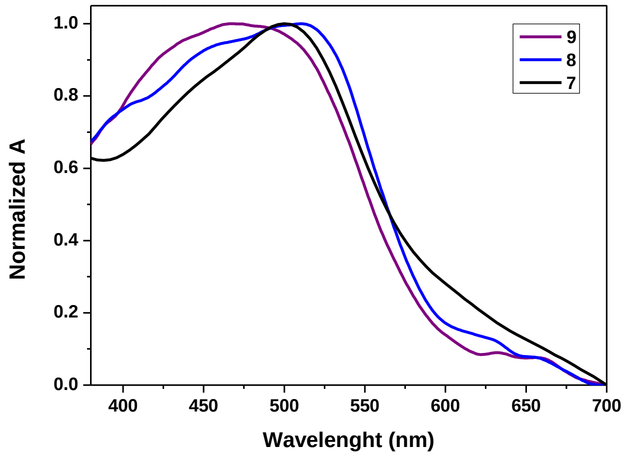

Figure S11. UV-visible diffuse reflectance of materials **7** and **8** and CTF **9**.

Imine-based material **7**: 501 nm
Hydrazone-based material **8**: 512 nm
CTF **9**: 469 nm

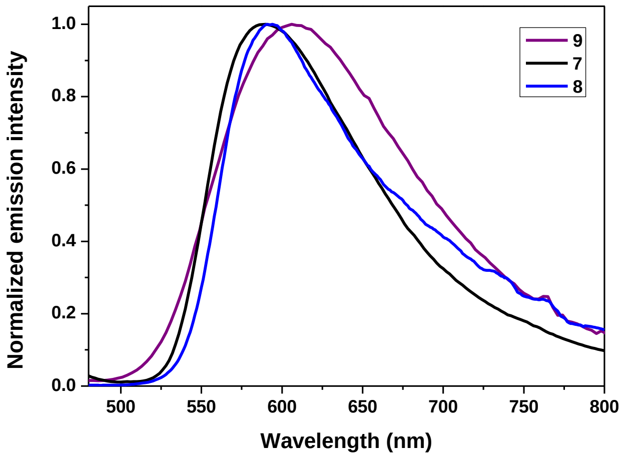

Figure S12. Emission spectra (λ_{exc} = 450 nm) of materials **7** and **8** and CTF **9**.

Imine-based material **7**: 588 nm
Hydrazone-based material **8**: 592 nm
CTF **9**: 607 nm

Kubelka-Munk Plots

Direct Band Gap Semiconductor

Imine-based material **7** = 2.26 eV (548 nm)

Hydrazone-based material **8** = 2.24 eV (553 nm)

CTF **9** = 2.30 eV (540 nm)

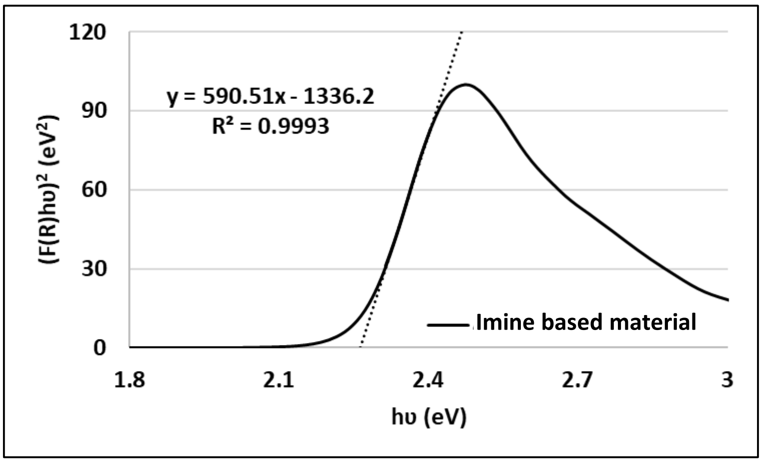

Figure S13. Direct Kubelka-Munk plot for imine-based material **7**.

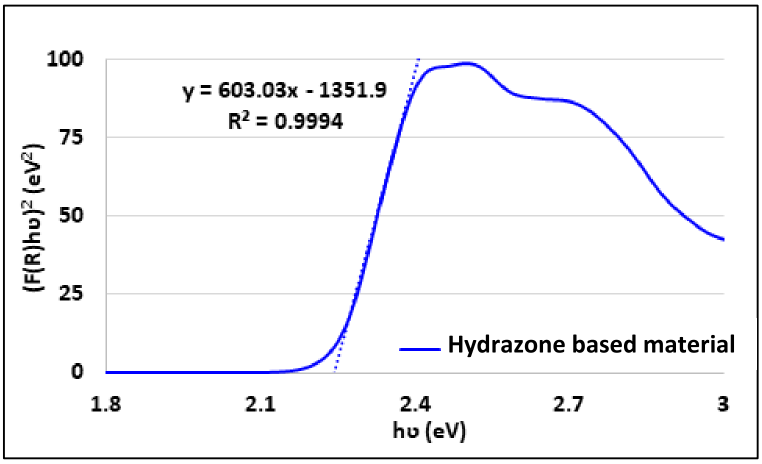

Figure S14. Direct Kubelka-Munk plot for hydrazone-based material **8**.

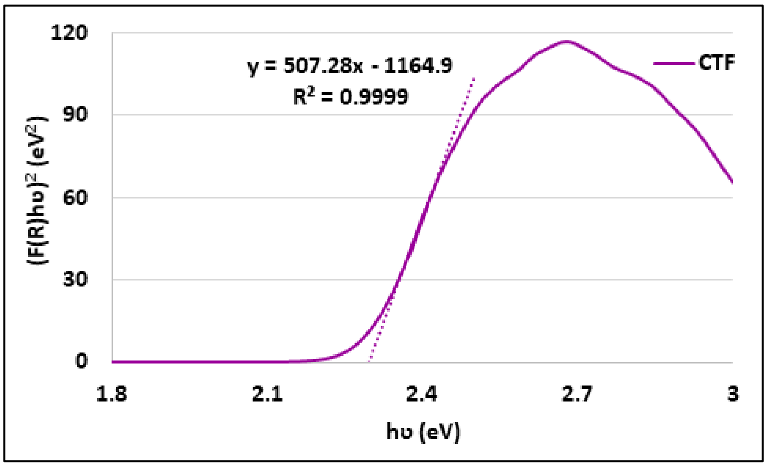

Figure S15. Direct Kubelka-Munk plot for CTF **9**.

11. ^{13}C Nuclear Magnetic Resonance of Solids

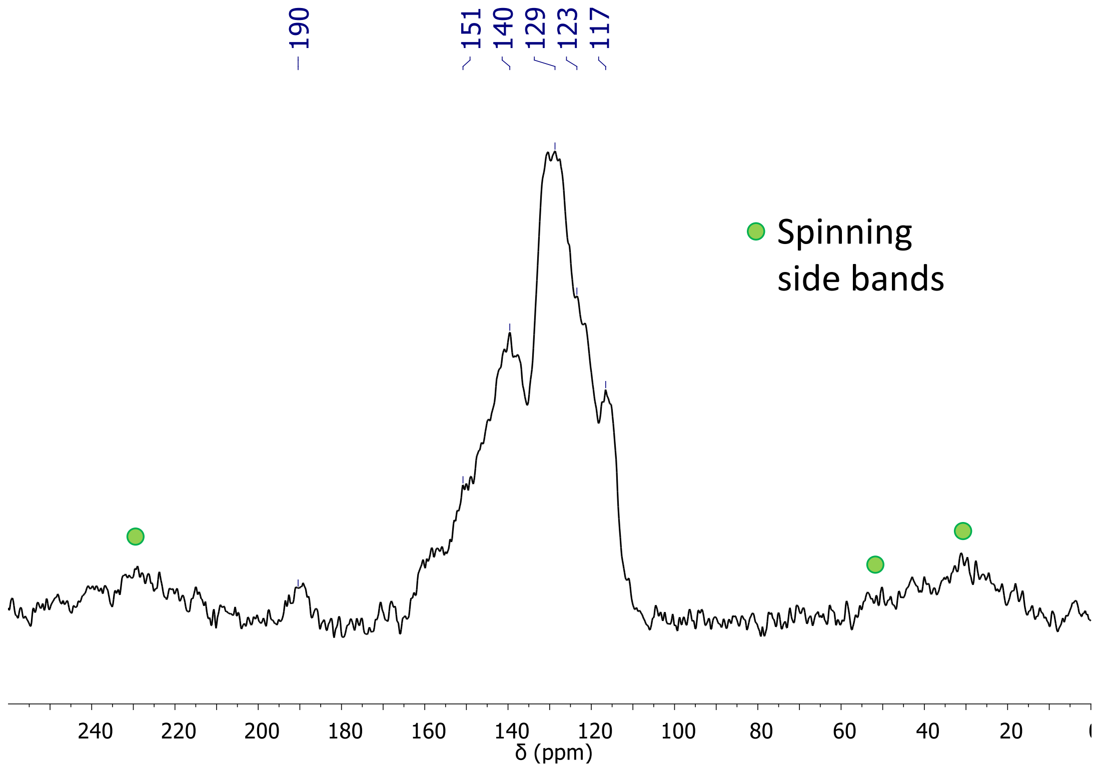

Signal (ppm)	Assignment
190	Aldehyde carbon (from the unreacted aldehydes)
151	Imine carbon
140, 129, 123, 117	Aromatic carbon

Figure S16. CP-MAS-^{13}C-NMR spectra of material **7.**

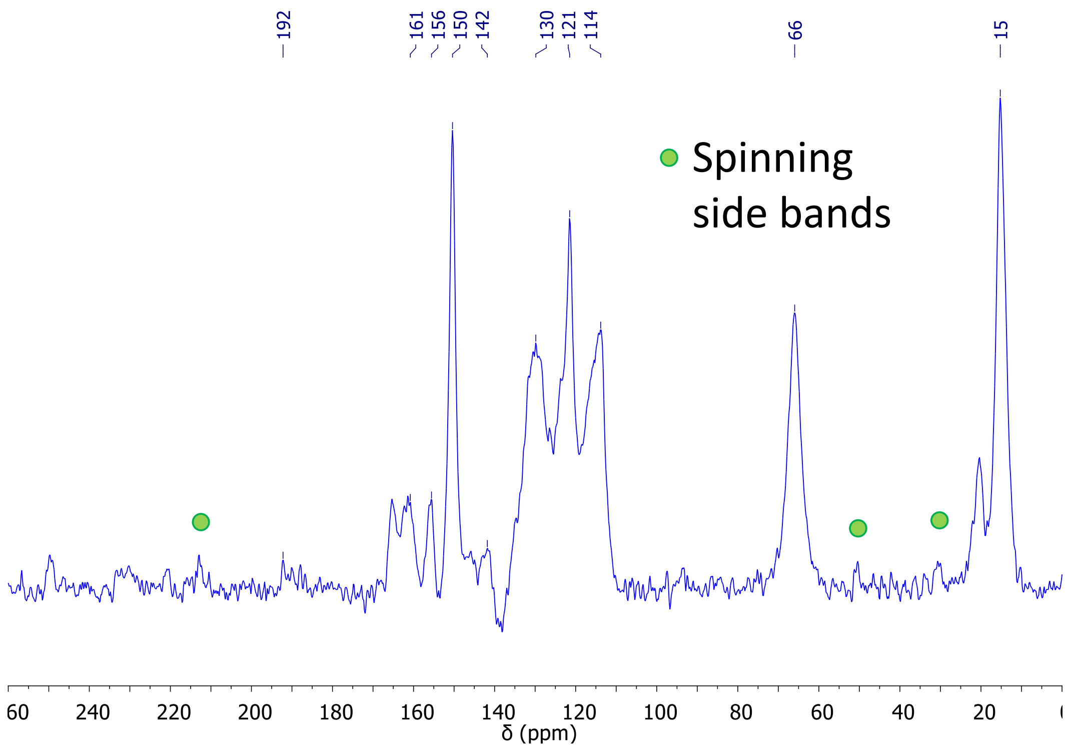

Signal (ppm)	Assignment
192	Aldehyde carbon (from the unreacted aldehydes)
161	Carbonyl carbon
150	Imine carbon
156, 142, 130, 121, 114	Aromatic carbon
66	O-CH_2
15	C-CH_3

Figure S17. CP-MAS-^{13}C-NMR spectra of material **8.**

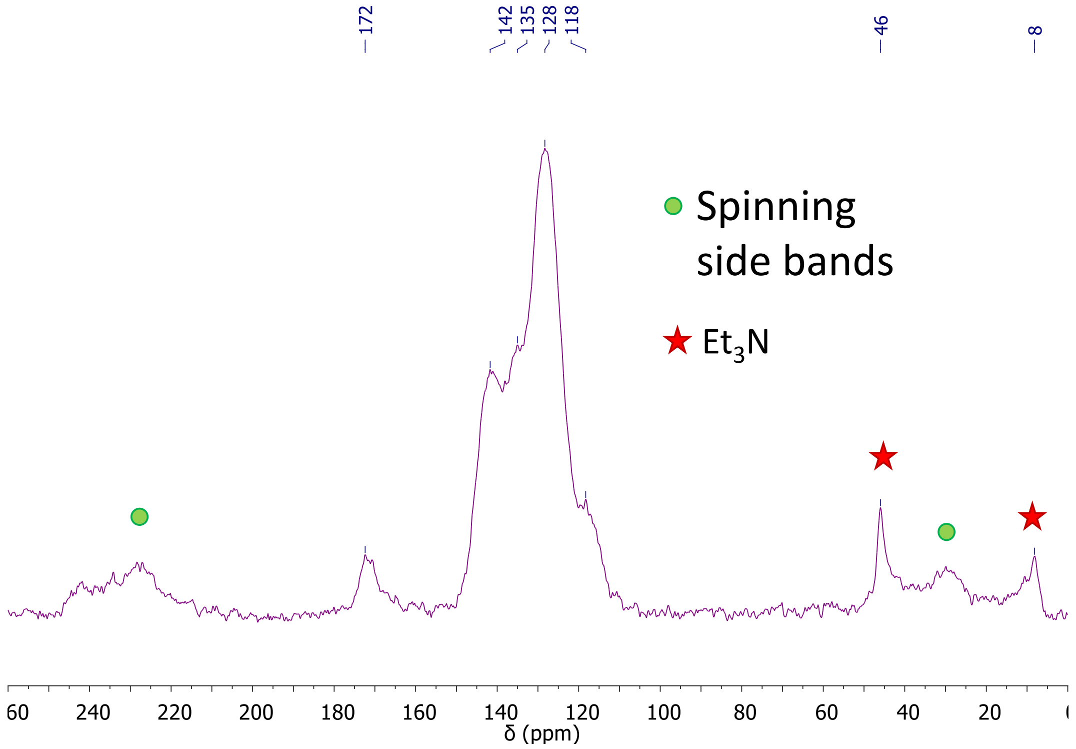

Signal (ppm)	Assignment
172	Triazine carbon
135	Nitrile carbon
142, 128, 118	Aromatic carbon

Figure S18. CP-MAS-^{13}C-NMR spectra of CTF **9.**

12. BET Isotherms

BET Surface Area: **12 m^2 g^{-1}**

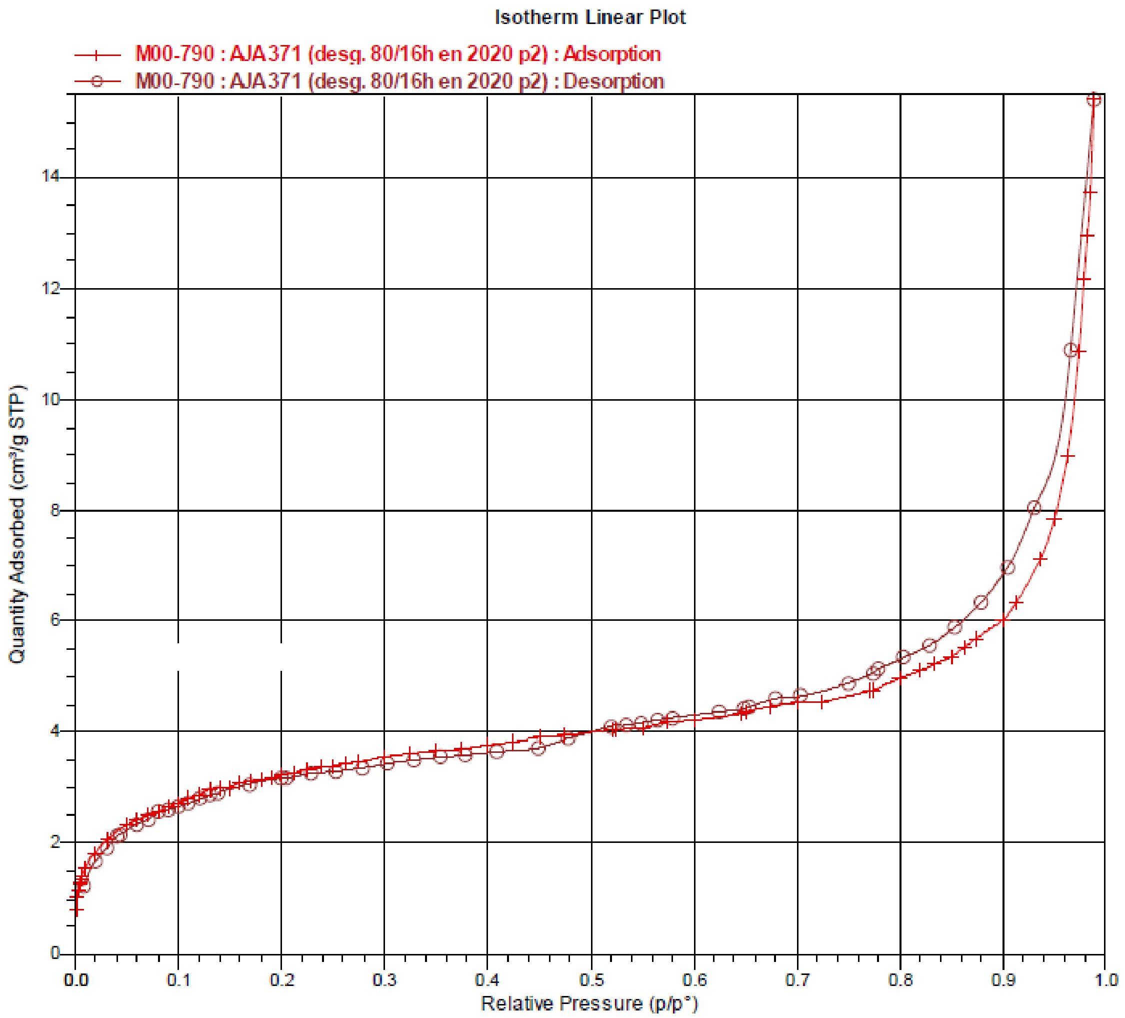

Figure S19. Adsorption isotherm of material **7.**

BET Surface Area: **45 m^2 g^{-1}**

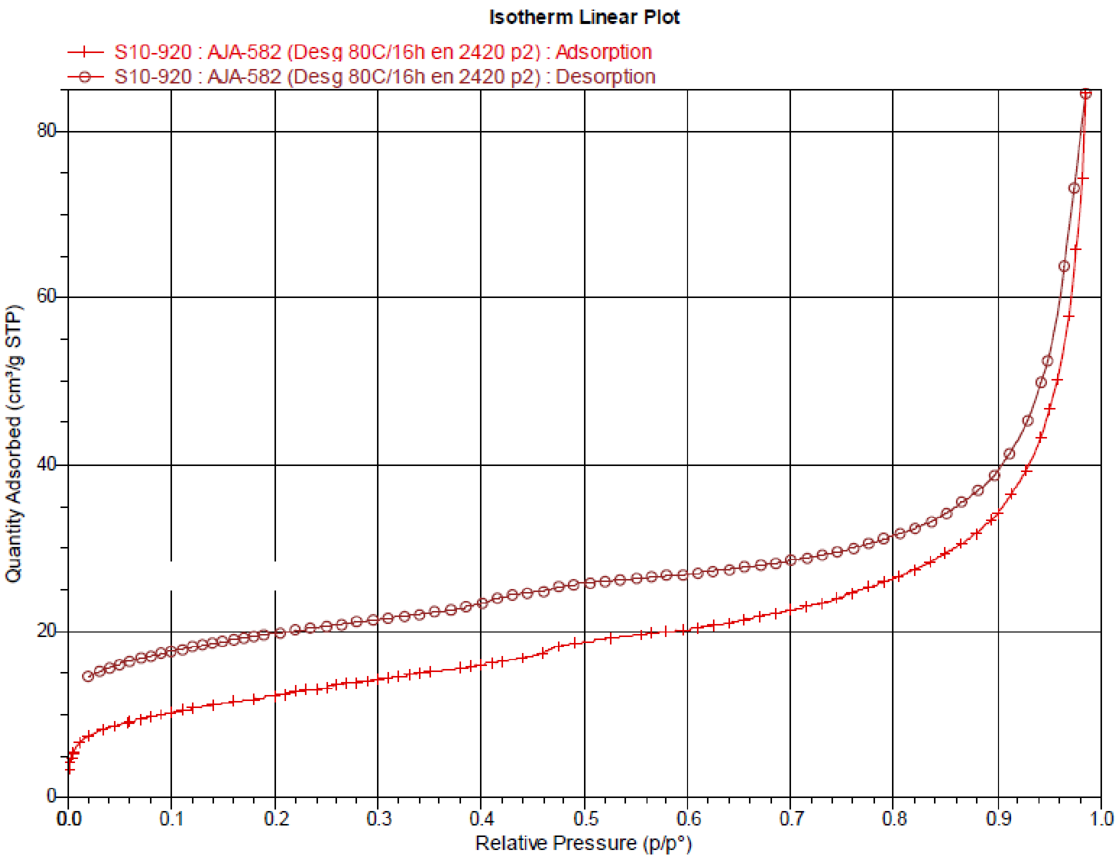

Figure S20. Adsorption isotherm of material **8.**

BET Surface Area: **68 m^2 g^{-1}**

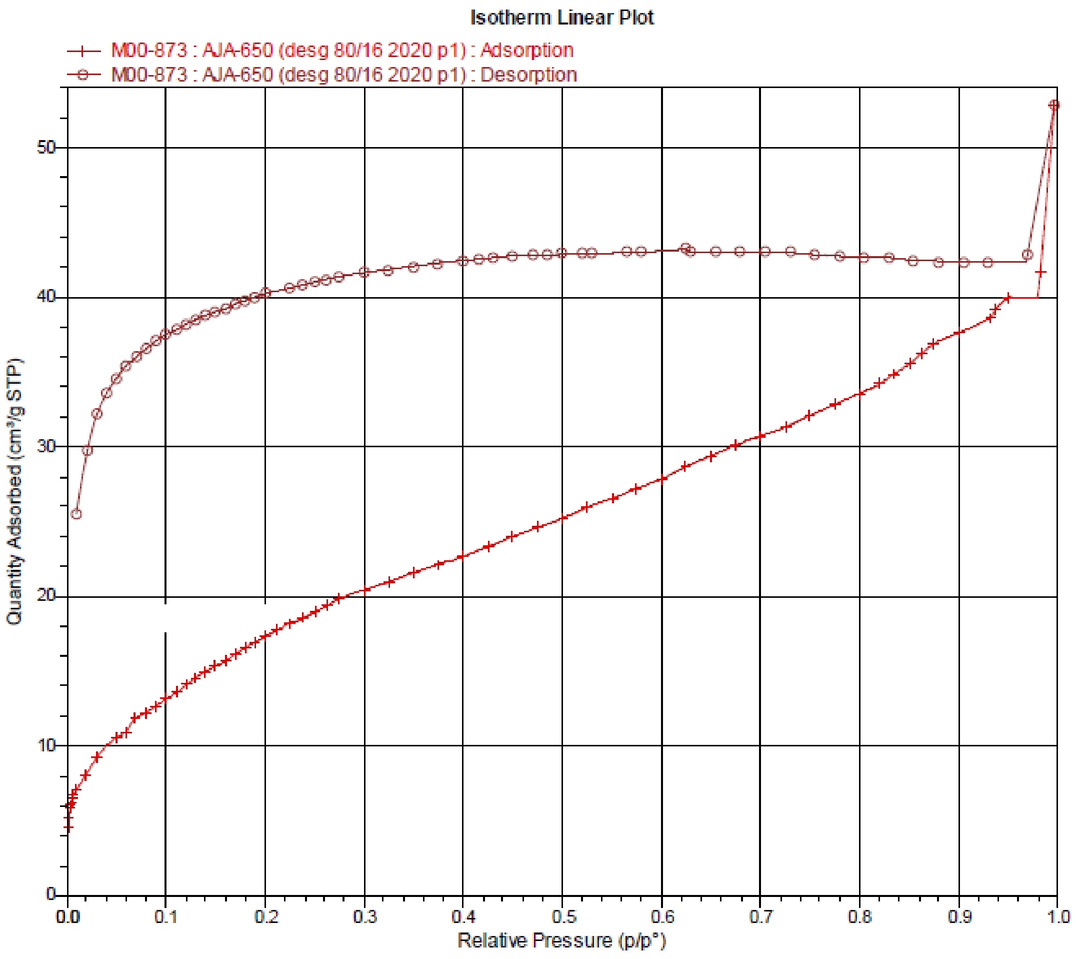

Figure S21. Adsorption isotherm of CTF **9.**

13. Scanning Electron Microscopy

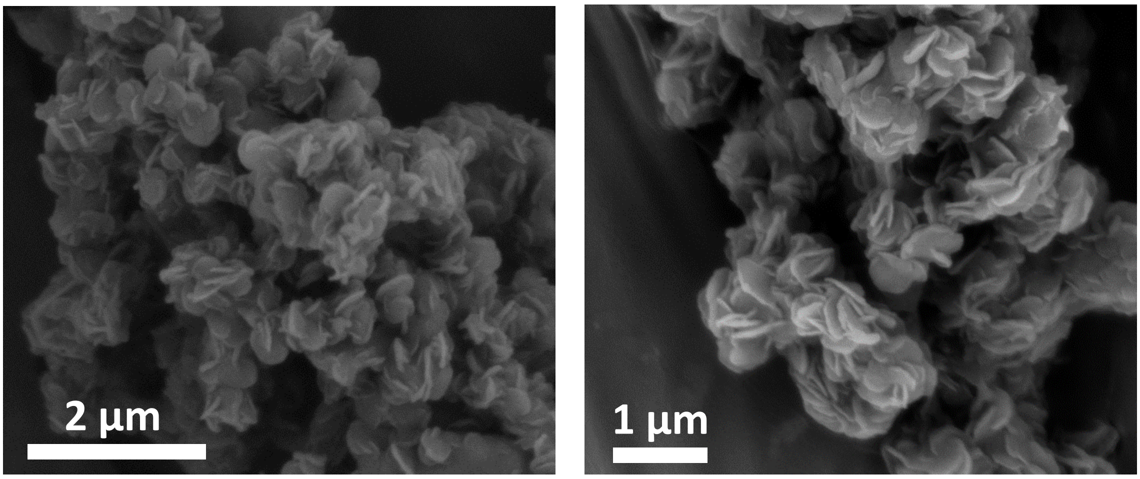

Figure S22. SEM images of material **7.**

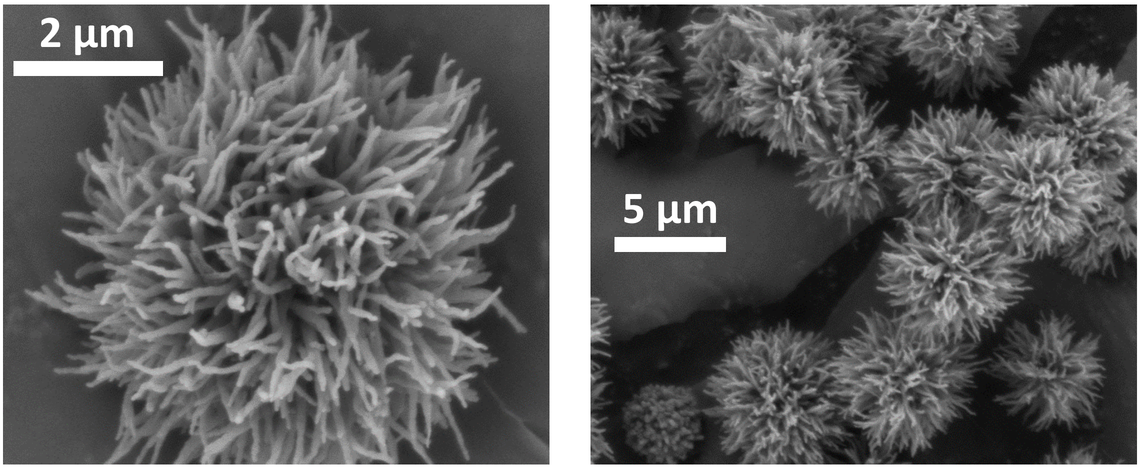

Figure S23. SEM images of material **8.**

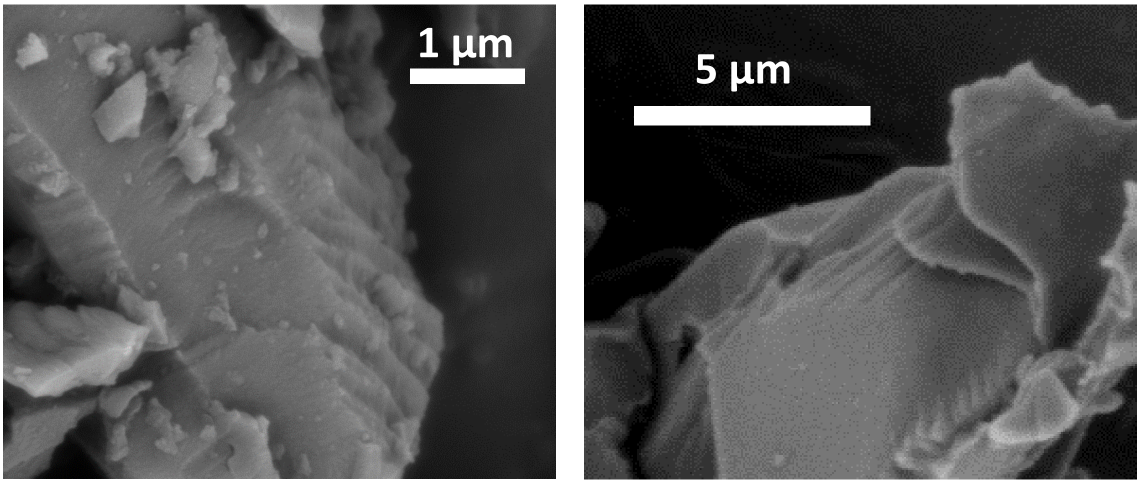

Figure S24. SEM images of CTF **9.**

14. Thermogravimetric analysis

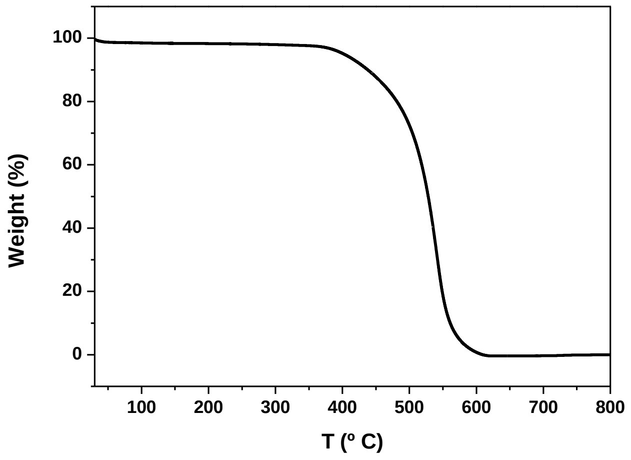

Figure S25. Thermogravimetric analysis of material 7.

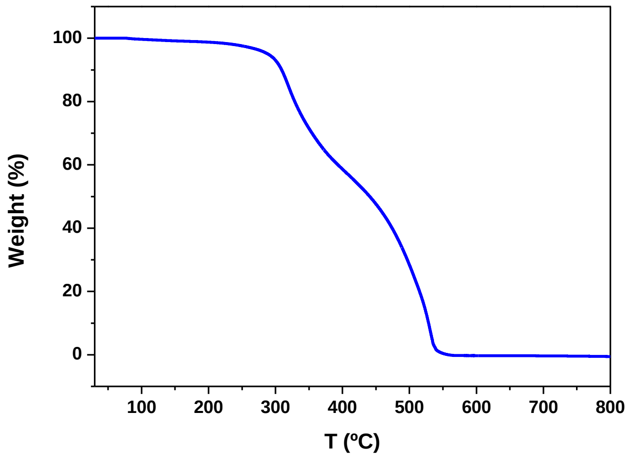

Figure S26. Thermogravimetric analysis of material 8.

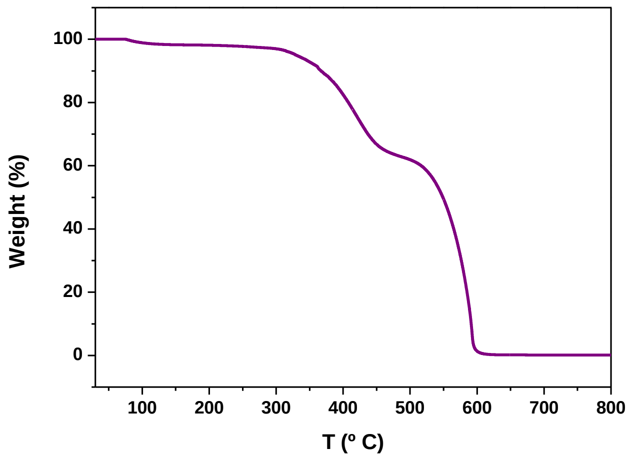

Figure S27. Thermogravimetric analysis of material 9.

15. Electrochemistry

Voltammetry experiments were performed under argon atmosphere at room temperature, using 0.1 M tetrabutylammonium hexafluorophosphate solution in CH_3CN as electrolyte. Measurements were carried out by using an Ivium CompaqStat potentiostat interfaced with a computer. A standard three-electrode electrochemical cell was used. Potentials were referred to an Ag/AgCl, Et_4NBr 0.4 M reference electrode in ethylene glycol, and measured potentials were calibrated using an internal Fc/Fc+ standard. The working electrode consists of a hollow electrode filled with a hybrid paste. The hybrid was fabricated by mixing 30 mg of high purity carbon (>99.9%) with 20 mg of the material and 1 drop of mineral oil (Nujol). As control experiment, a blank carbon paste containing only high purity carbon and Nujol was obtained and measured under the same conditions.

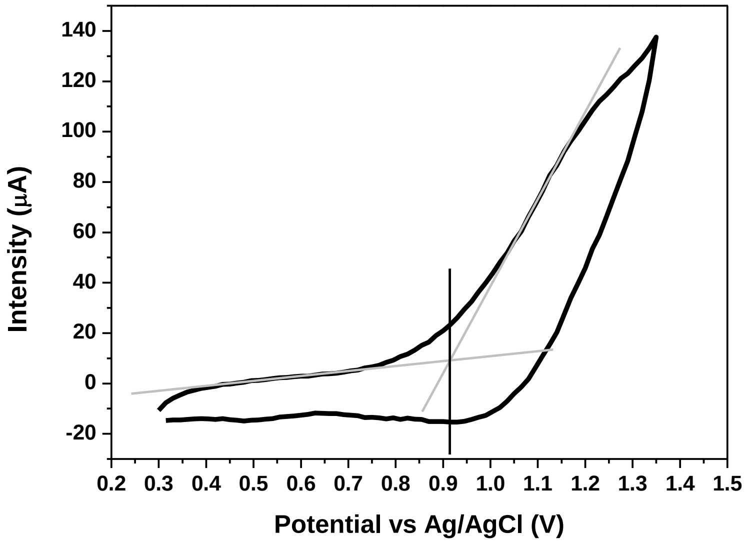

Figure S28: Cyclic voltammetry of material **7**.

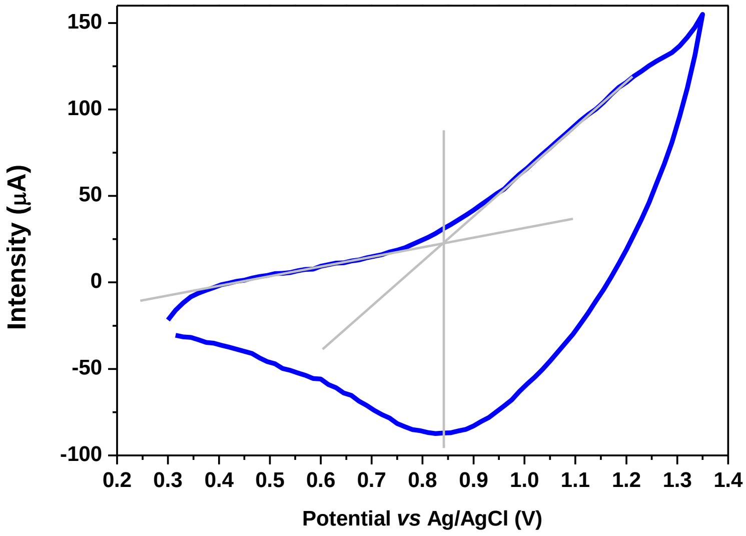

Figure S29: Cyclic voltammetry of material **8**.

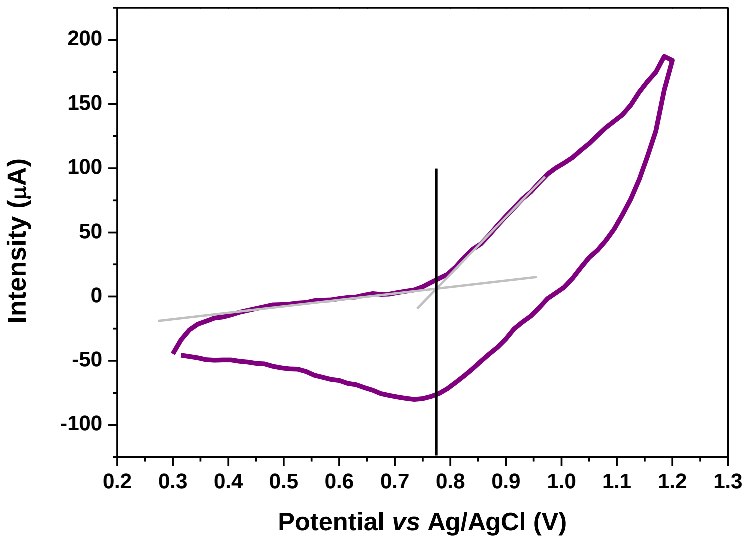

Figure S30: Cyclic voltammetry of CTF **9**.

16. Determination of Energy Levels

The value of the Valence Band (VB) was estimated by applying this equation:

$$E_{VB} = -4.44\ eV - (E_{ox} + E_{ref})$$

The value of the conduction band was estimated by applying this equation:

$$E_{CB} = E_{VB} + \text{Band Gap}$$

Table S7: Determination of Energy Levels of materials 7, 8 and 9.

	E_{ox} (V)	$E_{ox} + E_{ref}$ (V)	E_{VB} (eV)	Band Gap (eV)	E_{CB} (eV)
Material **7**	0,915	1,128	-5,57	2,26	-3,31
Material **8**	0,840	1,053	-5,49	2,24	-3,25
CTF **9**	0,770	0,983	-5,42	2,30	-3,12

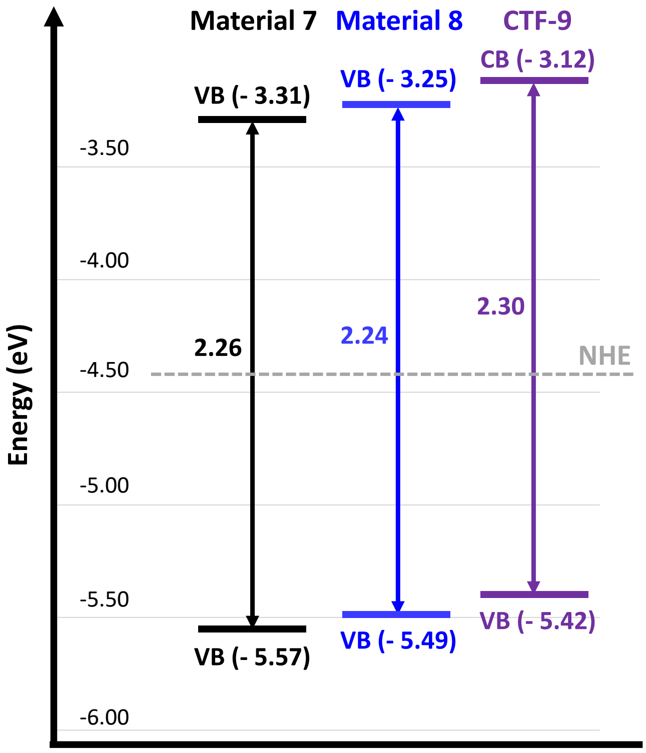

Figure S31: Energy Levels of 7, 8 and 9 calculated from the combination between DRS and electrochemical measurements.

17. Benchmarking of catalytic results

Table S8. Benchmarking of catalytic results.

Entry	Catalyst	Scale (mmol)	Catalyst loading (mg)	Time	Atm	Light source	Recyclability	REF
1	CTF 9	0.2	2	14 h	O2	Blue LEDs	7 cycles	This work
2	Cyanovinylene COF	0.2	5	12 h	Air	Green LEDs	4 cycles	[3]
3	Hydrazone COF	0.2	5.6	24 h	Air	Blue LEDs	6 cycles	[11]
4	Imine COF	0.2	10	70 min	Air	White LEDs	No	[12]
5	Cyanovinylene COF + TEMPO	0.5	Not indicated	20 min	Air	Red LEDs	No	[13]
6	β-ketoenamine COF	0.9	10	8 h	O2	Blue LEDs	5 cycles	[14]
7	CTF	0.33	17	12 h	O2	No light 60 ° C	No	[15]
8	CTF	1.0	60	10 h	O2	No light 120 ° C	No	[16]

18. References

(1) Jiménez-Almarza, A.; López-Magano, A.; Cano, R.; Ortín-Rubio, B.; Díaz-García, D.; Gomez-Ruiz, S.; Imaz, I.; Maspoch, D.; Mas-Ballesté, R.; Alemán, J. Engineering Covalent Organic Frameworks in the Modulation of Photocatalytic Degradation of Pollutants under Visible Light Conditions. *Mater. Today Chem.* **2021**, *22*, 100548. https://doi.org/https://doi.org/10.1016/j.mtchem.2021.100548.

(2) Bunck, D. N.; Dichtel, W. R. Bulk Synthesis of Exfoliated Two-Dimensional Polymers Using Hydrazone-Linked Covalent Organic Frameworks. *J. Am. Chem. Soc.* **2013**, *135* (40), 14952–14955. https://doi.org/10.1021/ja408243n.

(3) Li, S.; Li, L.; Li, Y.; Dai, L.; Liu, C.; Liu, Y.; Li, J.; Lv, J.; Li, P.; Wang, B. Fully Conjugated Donor–Acceptor Covalent Organic Frameworks for Photocatalytic Oxidative Amine Coupling and Thioamide Cyclization. *ACS Catal.* **2020**, *10* (15), 8717–8726. https://doi.org/10.1021/acscatal.0c01242.

(4) Girish, Y. R.; Biswas, R.; De, M. Mixed-Phase 2D-MoS2 as an Effective Photocatalyst for Selective Aerobic Oxidative Coupling of Amines under Visible-Light Irradiation. *Chem. – A Eur. J.* **2018**, *24* (52), 13871–13878. https://doi.org/https://doi.org/10.1002/chem.201802468.

(5) Wang, K.; Jiang, P.; Yang, M.; Ma, P.; Qin, J.; Huang, X.; Ma, L.; Li, R. Metal-Free Nitrogen -Doped Carbon Nanosheets: A Catalyst for the Direct Synthesis of Imines under Mild Conditions. *Green Chem.* **2019**, *21* (9), 2448–2461. https://doi.org/10.1039/C9GC00908F.

(6) Wendlandt, A. E.; Stahl, S. S. Chemoselective Organocatalytic Aerobic Oxidation of Primary Amines to Secondary Imines. *Org. Lett.* **2012**, *14* (11), 2850–2853. https://doi.org/10.1021/ol301095j.

(7) Pérez, J. F.; Llanos, J.; Sáez, C.; López, C.; Cañizares, P.; Rodrigo, M. A. Electrochemical Jet-Cell for the in-Situ Generation of Hydrogen Peroxide. *Electrochem. commun.* **2016**, *71*, 65–68. https://doi.org/https://doi.org/10.1016/j.elecom.2016.08.007.

(8) Eisenberg, G. Colorimetric Determination of Hydrogen Peroxide. *Ind. Eng. Chem. Anal. Ed.* **1943**, *15* (5), 327–328. https://doi.org/10.1021/i560117a011.

(9) Miyamoto, S.; Martinez, G. R.; Martins, A. P. B.; Medeiros, M. H. G.; Di Mascio, P. Direct Evidence of Singlet Molecular Oxygen [O2 (1Δg)] Production in the Reaction of Linoleic Acid Hydroperoxide with Peroxynitrite. *J. Am. Chem. Soc.* **2003**, *125* (15), 4510–4517. https://doi.org/10.1021/ja029262m.

(10) Luis-Barrerra, J.; Cano, R.; Imani-Shakibaei, G.; Heras-Domingo, J.; Pérez-Carvajal, J.; Imaz, I.; Maspoch, D.; Solans-Monfort, X.; Alemán, J.; Mas-Ballesté, R. Switching Acidic and Basic Catalysis through Supramolecular Functionalization in a Porous 3D Covalent Imine-Based Material. *Catal. Sci. Technol.* **2019**, *9* (21), 6007–6014. https://doi.org/10.1039/C9CY01527B.

(11) Liu, Z.; Su, Q.; Ju, P.; Li, X.; Li, G.; Wu, Q.; Yang, B. A Hydrophilic Covalent Organic Framework for Photocatalytic Oxidation of Benzylamine in Water. *Chem. Commun.* **2020**, *56* (5), 766–769. https://doi.org/10.1039/C9CC07661A.

(12) He, H.; Fang, X.; Zhai, D.; Zhou, W.; Li, Y.; Zhao, W.; Liu, C.; Li, Z.; Deng, W. A Porphyrin-Based Covalent Organic Framework for Metal-Free Photocatalytic Aerobic Oxidative

Coupling of Amines. *Chem. - A Eur. J.* **2021**, *266237*, 14390–14395. https://doi.org/10.1002/chem.202102239.

(13) Shi, J.-L.; Chen, R.; Hao, H.; Wang, C.; Lang, X. 2D Sp2 Carbon–Conjugated Porphyrin Covalent Organic Framework for Cooperative Photocatalysis with TEMPO. *Angew. Chemie Int. Ed.* **2020**, *n/a* (n/a). https://doi.org/10.1002/anie.202000723.

(14) Wu, Z.; Huang, X.; Li, X.; Hai, G.; Li, B.; Wang, G. Covalent-Organic Frameworks with Keto-Enol Tautomerism for Efficient Photocatalytic Oxidative Coupling of Amines to Imines under Visible Light. *Sci. China Chem.* **2021**, *64* (12), 2169–2179. https://doi.org/10.1007/s11426-021-1088-2.

(15) Abednatanzi, S.; Derakhshandeh, P. G.; Leus, K.; Vrielinck, H.; Callens, F.; Schmidt, J.; Savateev, A.; van der Voort, P. Metal-Free Activation of Molecular Oxygen by Covalent Triazine Frameworks for Selective Aerobic Oxidation. *Sci. Adv.* **2020**, *6* (14), 1–11. https://doi.org/10.1126/sciadv.aaz2310.

(16) Zheng, H.; Shi, S.; Wang, X.; Zhao, L.; Zhu, G.; Liu, M.; Gao, J.; Xu, J. Covalent Triazine Frameworks as Metal Free Catalysts for the Oxidative Coupling of Amines to Imines. *ChemistrySelect* **2019**, *4* (17), 5073–5080. https://doi.org/10.1002/slct.201901272.
